# AAV9 Gene Therapy in GM1 Gangliosidosis Type II: A Phase 1/2 Trial

**DOI:** 10.1101/2025.07.28.25332074

**Published:** 2025-07-29

**Authors:** Connor J. Lewis, Precilla D’Souza, Jean M. Johnston, Maria T. Acosta, Cristan Farmer, Eva H. Baker, Anna Crowell, Yoliann Mojica, Sumaiya Rahman, Lisa Joseph, Adam Hartman, Gilbert Vézina, Zenaide Quezado, Muhammad H. Yousef, Amelia Luckett, Zeynep Vardar, Mohammad Salman Shazeeb, Manuela Corti, Meghan Blackwood, Kirsten Coleman, Audrey Thurm, Erika De Boever, William A. Gahl, Barry J. Byrne, Terence R. Flotte, Xuntian Jiang, Amanda L. Gross, Allison M. Keeler, Heather Gray-Edwards, Douglas R. Martin, Miguel Sena-Esteves, Cynthia J. Tifft

**Affiliations:** 1 Office of the Clinical Director, National Human Genome Research Institute, Bethesda, MD, USA.; 2 Medical Genetics Branch, National Human Genome Research Institute, Bethesda, MD, USA.; 3 Neurodevelopmental and Behavioral Phenotyping Service, National Institute of Mental Health, Bethesda, MD, USA.; 4 Department of Diagnostic Radiology, National Institutes of Health Clinical Center, Bethesda, MD, USA.; 5 Division of Clinical Research, National Institute of Neurological Disorders and Stroke, Rockville, MD, USA.; 6 Division of Diagnostic Imaging and Radiology, Children’s National Hospital, Washington DC, USA.; 7 Department of Perioperative Medicine, National Institutes of Health Clinical Center, Bethesda, MD, USA.; 8 Department of Radiology, University of Massachusetts Chan Medical School, Worcester, MA, USA.; 9 Department of Pediatrics, University of Florida, Gainesville, FL, USA.; 10 Powell Gene Therapy Center, University of Florida, Gainesville, FL, USA.; 11 Horae Gene Therapy Center and The Li Weibo Institute for Rare Diseases Research, University of Massachusetts Chan Medical School, Worcester, MA, USA.; 12 EDB Consulting, Haverford, PA, USA.; 13 Department of Cellular and Genetic Medicine, University of Massachusetts Chan Medical School, Worcester, MA, USA.; 14 Department of Medicine, Washington University School of Medicine, St. Louis, MO, USA.; 15 Scott-Ritchey Research Center, Department of Anatomy, Physiology and Pharmacology, College of Veterinary Medicine, Auburn University, Auburn, AL, USA.; 16 Department of Neurology, University of Massachusetts Chan Medical School, Worcester, MA, USA.

## Abstract

**Background:**

GM1 gangliosidosis, caused by biallelic variants in *GLB1*, results from deficiency of lysosomal β-galactosidase, the enzyme primarily responsible for degradation of GM1 ganglioside. This progressive neurodegenerative disease is uniformly fatal with no approved therapies, but preclinical studies utilizing gene therapy have shown promising results.

**Methods:**

This phase 1–2 open label dose escalation study utilized a single intravenous administration of adeno-associated virus serotype 9 (AAV9) encoding β-galactosidase in the first nine enrolled Type II GM1 gangliosidosis participants. The primary endpoint was safety after 3 years; secondary and exploratory efficacy outcomes included cerebrospinal fluid (CSF) GM1 ganglioside and β-galactosidase, clinical assessments, and neuroimaging changes.

**Results:**

One serious adverse event was attributed to the vector, i.e., vomiting requiring rehospitalization for IV hydration. Serum aspartate and alanine aminotransferase levels increased following gene transfer but returned to baseline by 18 months. Per protocol analysis found stability in Vineland Expressive Communication and Gross Motor and significant declines in Fine Motor and Receptive Communication. Median CGI-Improvement scores at 2 and 3 years after gene transfer were “minimally improved” or “no change”. CSF β-galactosidase increased and GM1 ganglioside decreased in all participants. MRI showed improved myelination by differential tractography and declines in cerebral atrophy. MRS showed reduced loss of *N*-acetylaspartate+*N*-acetylaspartyl glutamate (NAA) compared with historical controls.

**Conclusions:**

A single IV infusion of AAV9 encoding β-galactosidase was well-tolerated among the first nine Type II GM1 gangliosidosis participants. Secondary and exploratory outcomes suggested improvements in biochemical markers and neuroimaging and stabilized or reduced rates of developmental deterioration (NCT03952637).

## INTRODUCTION

GM1 gangliosidosis (GM1) is a rare, progressive neurodegenerative lysosomal storage disorder caused by biallelic variants in *GLB1* encoding β-galactosidase, an enzyme that catabolizes GM1 ganglioside.^1^ Because neurons have a high content of GM1 ganglioside, the clinical manifestations of GM1 largely involve the central nervous system (CNS). Type I GM1 is the most severe subtype and Type III is the mildest. Type II GM1 patients present with developmental delays, ataxia, dystonia, dysarthria, low bone mineral density, and progressive brain atrophy.^2–5^ Two Type II GM1 subtypes, late-infantile and juvenile, have different clinical trajectories, but all patients experience a progressive, uniformly fatal course.^6,7^

There are no approved therapies for GM1, making gene replacement attractive, especially since preclinical studies of intravenous adeno-associated virus serotype 9 (AAV9) gene therapy in a feline GM1 model resembling Type II disease demonstrated increased survival, repletion of cerebrospinal fluid (CSF) enzyme levels, and correction of magnetic resonance imaging (MRI) and magnetic resonance spectroscopy (MRS) abnormalities.^8,9^ We performed a clinical trial of *GLB1* delivery utilizing AAV9 in 12 children with Type II GM1 (NCT03952637).

## METHODS

### Study Design

This 3-year phase 1–2 open label dose escalation study had 2 years of additional follow-up. Eligible children were age 6 months - 12 years with biallelic *GLB1* variants, β-galactosidase deficiency, a Type II GM1 gangliosidosis phenotype, and a Vineland-3 Adaptative Behavior Composite standard score ≥ 40 (Appendix A). Individuals were excluded if their anti-AAV9 titer was > 1:50. All participants received AAV9-GLB1 (1.5×10^13^ or 4.5×10^13^ vector genomes/kg) intravenously 1 mL/min (Appendix B). Immune suppression was achieved with rapamycin and Rituximab 3 weeks before gene transfer, methylprednisolone 1–2 hours before gene transfer, and oral prednisone following gene therapy (Appendix C). Blood draws, lumbar punctures, Clinical Global Impression Improvement (CGI-I) scores, Vineland-3 Adaptive Behavior Scales, and MRI and MRS images were obtained periodically (Appendicies D, E, and F).

### End Points

The primary end point was safety at 3 years post-infusion, i.e., adverse events (AE) or serious adverse events (SAE). Key secondary outcomes included the CGI-Improvement (CGI-I) rating and Vineland Adaptive Behavior Scales (Appendix E) at 2 years post-infusion, changes in brain MRI (volumetric analysis, diffusion tensor imaging [DTI]), and MRS (Appendix F). Exploratory endpoints included CSF GM1 ganglioside, CSF and serum β-galactosidase activity, and H3N2b (a unique oligosaccharide in GM1) in CSF, plasma, and urine (Appendix D).^10^

### Statistical Analysis

For this rare condition, the planned sample size ceiling was based on feasibility; no a priori power calculation was performed. The statistical analysis plan dictated an intent-to-treat mixed model for repeated measures controlling for baseline age to evaluate change from baseline on the Vineland GSV at Year 2; the Year 3 timepoint was also included (Appendix E). Alpha was set at 5%, uncorrected for multiplicity. Longitudinal biochemical and neuroimaging data were descriptive. Post-hoc exploratory analysis of safety and biochemical outcomes was performed by dosage group and analysis of developmental and neuroimaging by subtype due to known differential clinical trajectories.^5,6,11,12^

## Results

### Study Population; Baseline Values (Table 1)

Nine children (5 males) were enrolled between August 19, 2019 and October 24, 2022; at the cutoff date for this interim analysis (June 1, 2025), GT17 had not reached 3 years of follow-up. Five participants (four late-infantile) received low dose and four (all juvenile) received high dose in order of enrollment. Individual Vineland and CGI scores reflected marked GM1 disease at baseline in most participants (Table 1). The mean CSF β-galactosidase activity ranged from 0–10% of normal and the mean CSF GM1 ganglioside levels were 2–5-fold normal. Baseline AST values ranged from 39–146 U/L (normal, ≤41), while ALT and GGT levels were normal. All participants had an AAV9 titer ≤1:50.

### Safety

One SAE (GT17) involved vomiting requiring hospitalization for IV fluids on day 3; this was definitely related to the treatment. Four other SAEs (Supplement Table G1) were considered unrelated to AAV9-GLB1, with two attributable to GM1 disease progression, one to the immunosuppression regimen, and one to protocol-related procedures.

There were 113 other AEs (Supplement Table G2); 72 were Grade 1, 28 Grade 2, and 13 Grade 3. Thirty AEs were considered possibly-, probably-, or definitely-related to the vector (Table 2). There were 8 gastrointestinal events potentially related to the vector, including vomiting (5), retching (1), and reduced appetite (2). One instance of tachycardia was possibly related to the vector.

**Figure 1.**
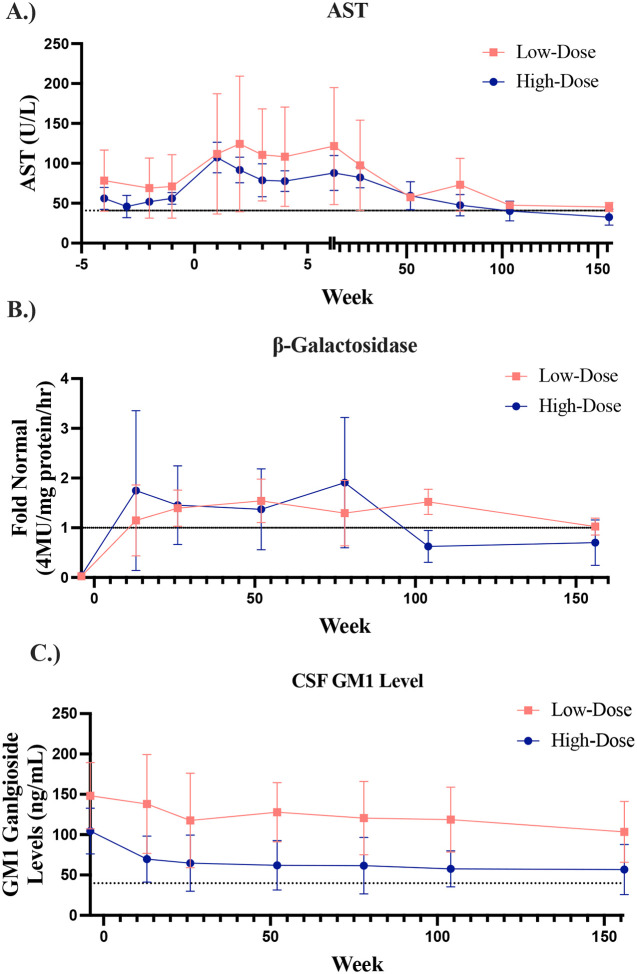
Biochemical Outcomes **A.** AST levels in participants after receiving low dose or high dose gene transfer (mean +/− SD). **B.** CSF β-galactosidase levels after low dose or high dose gene transfer. **C.** CSF GM1 levels after gene transfer. The dotted line represents the upper limit of normal for AST (41 U/L) and GM1 ganglioside levels in CSF (39.9 ng/mL at 1 standard deviation above normal),^8^ and represents the normal activity from pediatric controls for β-galactosidase (*n*=9 up to Week 78, *n*=8 up to Week 104 (Year 2), *n*=7 at Week 156 (Year 3).

There were 21 laboratory abnormalities related to the vector, including one elevated Elispot that resolved. Other AEs were elevated aspartate aminotransferase (AST, 9), D-dimer (5), ferritin (3), alanine aminotransferase (ALT, 2), or C-reactive protein (1). All 9 participants had elevated AST levels at baseline (Table 1), typical for the underlying disease. Mean AST levels were further elevated following gene transfer; elevations were similar between the low and high dose participants (Figure 1A). All AST levels fell to baseline 18 months after treatment (Supplementary Figure H1). ALT levels also increased post AAV9-GLB1 infusion but were generally resolved between week 52 and 78 following gene transfer (Supplement Figure H2). Gamma glutamyl transferase (GGT) levels were well controlled except in GT03, whose central catheter became infected on day 18 (Supplement Figure H3). Viral genomes were detected in saliva, urine, and feces following AAV9-GLB1 administration; all returned to low levels by day 30 (Supplement Figures I1–I3).

### Immune Responses to AAV9-GLB1

Complement (C3 and C4) was not depleted post gene transfer (Supplement Figures H4 and H5). Platelets declined slightly but remained above 100,000/mcL and returned to normal (Supplement Figure H6). There were transient increases in D-dimer (Supplement Figure H7). Anti-vector IgG typically peaked at 6–9 months (Supplemental Figure J1).

Anti-AAV9 IgM generally peaked 2–10 weeks post gene transfer (Supplemental Figure J2) and returned to baseline. Although Rituximab depleted circulating B-cells for at least 6 months, all participants developed anti-AAV9 neutralizing antibodies within 2 weeks after gene transfer, with an increase at 6 months and then stabilization for years (Supplement Figure J3).

Capsid-specific IFN-γ producing cells peaked in the first 10 weeks post treatment (Supplement Figure J4), corresponding to ALT and AST increases in most participants. Secondary capsid-specific IFN-γ immune responses occurred in GT06, GT08, GT10, and GT17 at 6 months to 1 year post AAV delivery and did not correspond to increases in ALT and AST. Increases in capsid-specific IFN-γ in GT10 and GT17 corresponded to loss of enzymatic activity in the serum but not in the CSF (Supplement Figure K3). Transgene-specific immune responses were not observed in any participant at any timepoint (Supplement Figure J5).

### Efficacy

#### Clinical Outcome Assessments

##### Vineland.

**Figure 2.**
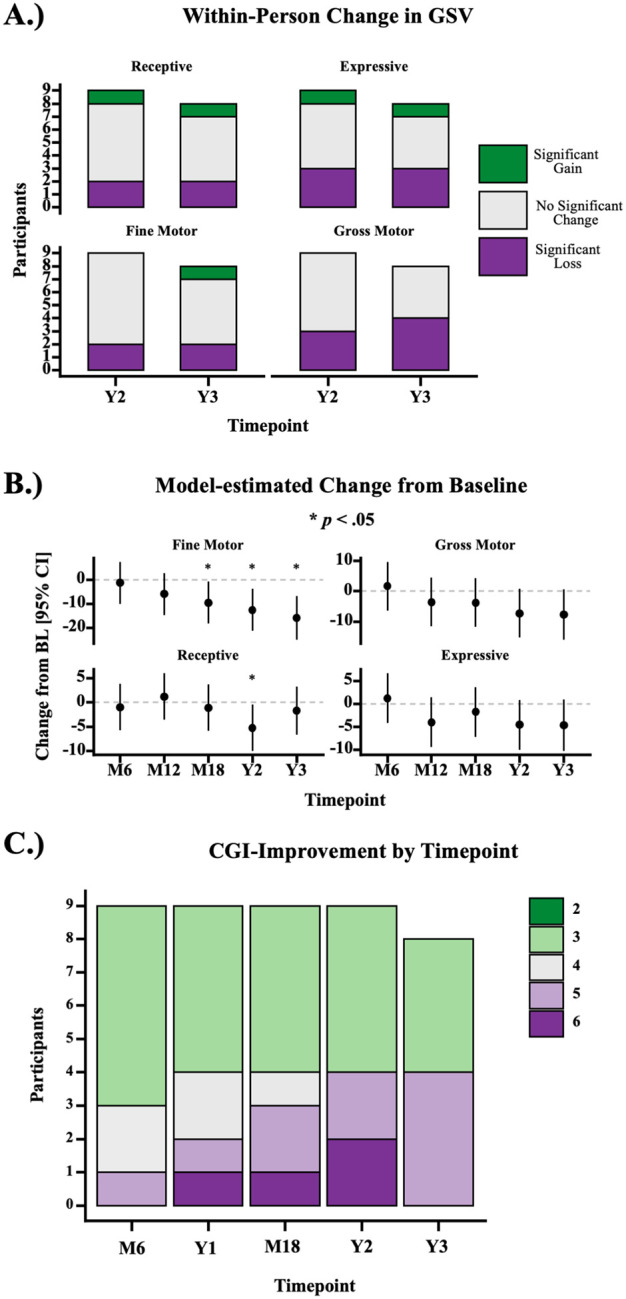
Clinical Outcome Assessments. **A.** N-of-1 analysis of change in Vineland-3 GSVs. The number of participants with significant (*p*<0.05) increases, decreases, or no significant change in GSV, based on the conditional standard error of measurement is shown by timepoint. **B.** Estimated marginal mean change from baseline at each timepoint, from the per protocol mixed model for repeated measures. Asterisks indicate statistically significant (*p*<0.05) change. **C.** The number of participants with each CGI-Improvement rating is shown by timepoint (*n*=9 up to Year 2, *n*=8 at Year 3).

Vineland scores of untreated GM1 participants decline with age.^11^ At years 2 and 3 (*n*=8), most gene transfer-treated participants’ Vineland-3 GSVs were not statistically different from their baseline scores (Figure 2A). However, the per protocol group-level analysis indicated statistically significant mean loss of skills in Fine Motor (Year 2: *t*(39) = −2.81, *p* = 0.008; Year 3: *t*(39) = −3.43, *p* = 0.001) and Receptive Communication (Year 2: *t*(39) = −2.15, *p* = 0.038; Year 3: n.s.) (Figure 2B). No significant changes in Expressive Communication and Gross Motor were observed. Individual Vineland Adaptive Behavior Growth Scale Values are shown in Supplementary Figures L1–L4.

##### CGI-Improvement.

In untreated GM1, the mean annualized change in CGI-I scores of late-infantile participants was + 0.61 (SE=0.07) and of juvenile GM1 participants + 0.17 (SE=0.11),^12^ indicating worsening function. In the 9 treated participants, the median CGI-I at Year 2 was 3 (“Minimally Improved”) [IQR = 3, 5] and at Year 3 (*n*=8) was 4 (“No Change”) [IQR = 3, 5] (Figure 2C). Individual participant scores are shown in Supplementary Figure L3.

#### Enzyme repletion

Mean CSF β-galactosidase levels increased from ~zero at baseline to approximately normal values for participants receiving either low or high dose gene transfer (Figure 1B). Mean CSF GM1 fell below baseline in both groups (Figure 1C).

Within 13–26 weeks of receiving low-dose AAV9-GLB1, all 4 late-infantile subjects showed normal CSF β-galactosidase levels; these remained above normal at the efficacy end point or the most recent evaluation (Supplement Figure K1). CSF GM1 ganglioside levels fell below baseline in all 4 participants at both one and two years post gene transfer (Supplement Figure K1). Serum β-galactosidase activity increased, peaking at 13-weeks following gene transfer in all 4 late-infantile participants (Supplement Figure K2). CSF, serum, and urine H3N2b levels decreased in all 4 late-infantile participants following gene transfer (Supplement Figure K3–K5).

CSF β-galactosidase activity increased in all five juvenile participants (*n*=4 high dose), reaching ≥normal levels in at 13 weeks and declining 2 years post gene transfer (Supplement Figure K1). CSF GM1 decreased in all 5 juvenile participants and typically reached a minimum between 6 months and 1 year following gene transfer (Supplement Figure K1). Serum β-galactosidase activity increased in all 5 juvenile participants and was higher in participants receiving the higher dose (Supplement Figure K2). H3N2b levels in CSF, serum, and urine decreased in all 5 juvenile participants (Supplement Figure K3–K5).

#### Neuroimaging; Differential Tractography

**Figure 3.**
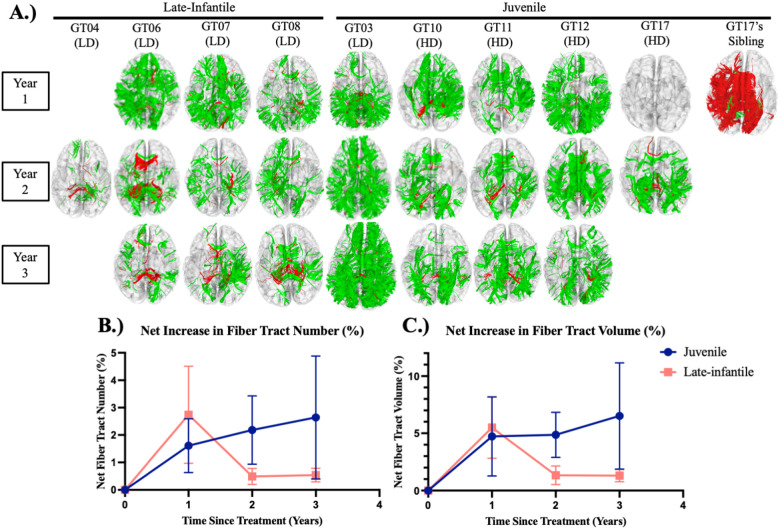
Neuroimaging Results. A.) Differential fiber tractography assessed fiber tract gains (green) and fiber tract losses (red) B.) Net fiber tract number in both cohorts. C.) Net fiber tract volume in both cohorts. LD = Low-Dose, HD = High-Dose (*n*=9 up to Year 2, *n*=8 at Year 3).

Differential tractography has documented consistent declines in neuronal tract numbers and volume in untreated GM1.^13^ One example is provided by the image of the untreated older sibling of GT17 over a one year period (Figure 3A; year 1), showing widespread decrements (red).

In contrast, all four late-infantile (low dose) GM1 participants showed gains in neuronal tracts 2 years post gene transfer, and GT06, GT07, and GT08 also had improvements at years 1 and 3 (Figure 3A). The greatest neuronal growth appeared in the first year after gene transfer. For the late-infantile subtype, differential tractography showed a mean net gain in fiber tract number of 2.7 ± 1.8 percent in the first year after gene transfer, falling to 0.5 ± 0.3 percent and 0.5 ± 0.2 percent by years 2 and 3 (Figure 3B). The mean net gain in fiber tract volume was 5.5 ± 2.7 percent, 1.3 ± 0.8 percent, and 1.3 ± 0.5 percent in years 1, 2, and 3 (Figure 3C). Neuroimaging of GT06, GT07, and GT08 showed reductions in the rate of global brain atrophy compared to the natural history (Section M of the Supplementary Appendix). All 4 late-infantile participants showed improvements in the rate of ventricle enlargement and thalamic atrophy compared to the natural history. GT07 and GT08 also showed reductions in the loss of NAA compared to historical controls, assessed by MRS (Supplement Figure M5).

Similar differential tractography results were obtained for the juvenile subtype (*n*=5; *n=*4 high dose) (Figure 3A). GT03, in particular, showed marked growth of neuronal tracts at years 1, 2, and 3. For the 5 GM1 juvenile subtype participants, differential tractography showed a mean net gain of 1.6 ± 1.0 percent, 2.2 ± 1.2 percent, and 2.6 ± 2.2 percent in fiber tract number at 1, 2, and 3 years after gene transfer (Figure 3B. The mean net gains in fiber tract volume were 4.7 ± 3.5 percent, 4.9 ± 2.0 percent, and 6.5 ± 4.6 percent in years 1, 2, and 3 (Figure 3C). GT03 and GT12 showed improvements in the rate of global brain atrophy compared to the natural history (Section M of the Supplementary Appendix). GT03, GT10, GT12, and GT17 showed improvements in the rate of ventricle enlargement compared to historical controls. GT03 and GT12 showed improvements in the rate of thalamic atrophy compared to the natural history. GT03, GT10, and GT12 also showed improvements in the loss of NAA compared to historical controls (Supplement Figure M5).

## DISCUSSION

GM1 gangliosidosis, a rare fatal monogenic disease with a well characterized natural history, represents a promising disorder for gene therapy.^11^ This phase 1–2 study provides evidence that a single intravenous infusion of AAV9 gene therapy carrying β-galactosidase is well-tolerated, and suggests that it increases β-galactosidase activity, decreases GM1 storage, slows brain atrophy, and allows for neuronal growth.

No substantial safety concerns arose from this clinical trial. Most adverse events appeared unrelated to AAV9-GLB1 administration and were moderate in severity. Only one serious adverse event was considered related to the vector. All participants were alive at the cutoff date. There was no evidence of thrombotic microangiopathy (TMA), hemophagocytic lymphohistiocytosis (HLH), or acute respiratory distress syndrome (ARDS), which have occurred during AAV therapeutics involving more than twice the viral load our high dose cohort received.^14–19^

AAV based gene therapies invoke immune responses,^20–24^ and all 9 participants in this report developed persistent neutralizing antibodies to the vector. Since Type II GM1 affects children, assessment of long-term transgene expression and innovative immunosuppression approaches are warranted for potential re-administration of the vector in the developing brain.^21,25^

Biochemical parameters provided preliminary evidence for efficacy. In the first nine participants, serum and CSF β-galactosidase levels increased above baseline levels and those of historical controls,^11^ reflecting *GLB1* transduction. Furthermore, reductions in the pentasaccharide biomarker, H3N2b, in urine, serum, and CSF, along with reductions in the cytotoxic substrate (GM1 ganglioside), indicated systemic increases in enzyme activity.^10^

Regarding development, the *a priori* hypothesis of improvement in Vineland-3 GSV was not supported. However, at Year 3, three of the four subdomains of interest remained not significantly different from baseline. The natural history of GM1 involves eventual declines among all affected individuals,^7,11^ so no change in 3 years may be a clinically meaningful outcome. Indeed, despite the statistically significant declines in some subdomains of the Vineland-3, the median CGI-I score was “minimally improved,” so some loss of skills may represent a clinically meaningful positive result.

Improvement in secondary radiologic outcomes corroborated the biochemical and clinical findings. Progressive brain atrophy with enlargement of the lateral ventricles is a neuroimaging hallmark of GM1 gangliosidosis;^5,11^ our gene therapy participants exhibited reduced rates of this atrophy, indicating structural CNS improvements. Differential tractography, reflecting myelination, supported this result, showing white matter trajectorial improvements in the AAV9-GLB1-treated individuals compared to historical controls.^13^

Baseline AAV9 antibody was higher in GT17 (1:50) than in other participants (< 1:25), and GT17 had a less beneficial response to the vector. In addition, GT04 was the youngest, most severely affected participant, with the highest GM1 ganglioside level, and was losing skills rapidly, as evidenced by baseline clinical outcome assessments. His poor response resembled that observed in metachromatic leukodystrophy where participants entering phases of rapid decline did not experience therapeutic benefit from gene therapy.^26^ In contrast, GT03, appeared pre-symptomatically in some areas due to the diagnosis in an older sibling, was the least severe at baseline and showed marked improvements in clinical outcomes and neuroimaging.

The apparent dose-related response to AAV9-GLB1 in terms of serum β-galactosidase activity was not observed in CSF enzyme activity, clinical outcome assessments, or neuroimaging. One possible explanation involves saturation of the blood brain barrier’s active transport of AAV9,^27^ while expression of *GLB1* from the liver could determine the dose-response in serum. The serum/CSF difference was not observed in our preclinical murine or feline models,^28,29^ and there are no human studies mentioning this phenomenon. Future investigations could involve the use of novel approaches for delivering AAV to the CNS, including administration into the cisterna magna, lateral ventricles, subarachnoid space, striatum, and the thalamus.^22,30,31^ Even more promising may be a new technology to target AAV capsids to the human transferrin receptor, greatly facilitating crossing of the blood brain barrier.^32^

Study limitations include its open-label design and clinical outcome assessment by unblinded clinicians. The small sample size prevented formal comparison of results between doses and/or subtype groups; further, subtype and dose groups were confounded. While the largest available natural history study^11^ is an important source of comparison, especially for CGI-I ratings,^12^ that study had older and more severely impacted participants than those in the current trial. Finally, 5 of the 9 participants were from 2 families (Table 1), perhaps limiting generalizibility.

This study provides preliminary evidence that administration of a single dose of AAV9-GLB1 is safe and well tolerated and suggests that it may lead to biochemical and neuroimaging improvements in Type II GM1 gangliosidosis. Future studies are needed to evaluate clinical benefits of earlier gene therapy and longer follow-up with respect to development as well as non-neurological manifestations of GM1, including its ophthalmologic, musculoskeletal, and hepatic symptoms.^11^ In addition, it will be important to determine the appropriate baseline anti-AAV antibody and clinical severity cutoff for AAV therapeutics in GM1 participants.

## Supplementary Material

1

## Figures and Tables

**Table 1.** Baseline Patient Characteristics and Vector Dosage. Specific ages were redacted per MedArXiv requirements.

Participant	GT04	GT06	GT07	GT08	GT03	GT10	GT11	GT12	GT17
Sex	M	M	F	F	M	F	F	M	M
Age (yr)	0–5	6–10	0–5	0–5	0–5	6–10	0–5	6–10	6–10
Weight (kg)	17.9	17.0	14.9	14.4	18.3	22.9	21.7	13.0	20.1
Disease Subtype	Late-Infantile	Late-Infantile	Late-Infantile	Late-Infantile	Juvenile	Juvenile	Juvenile	Juvenile	Juvenile
Attained Running*	No	No	No	No	Yes	Yes	Yes	Yes	Yes
Attained 3 Word Sentence^τ^	No^ω^	No	No	No	Yes	Yes	Yes	Yes	Yes
Baseline Vineland^†^	52	63	73	76	81	76	80	79	73
Baseline CGI-S^∥^	5	4	4	4	2	3	3	3	3
β-galactosidase activity (fold normal)^Ω^	0.00	0.01	0.00	0.04	0.07	0.06	0.01	0.00	0.10
GM1 ganglioside levels (ng/mL)^Ω^	175	75.8	163	172	155	128	67.4	97.6	125
AST (U/L)^ψ^	146	63	55	57	71	39	51	70	64
ALT (U/L)^ψ^	25	11	9	10	19	13	19	12	18
GGT (U/L)^ψ^	14	8	10	8	14	14	14	13	14
Baseline AAV9 Titer	1:50	< 1:25	< 1:25	< 1:25	< 1:25	< 1:25	< 1:25	< 1:25	1:50
IVGT Dose^¶^	Low	Low	Low	Low	Low	High	High	High	High

* Ability to run assessed at baseline assessment. Neurotypical children begin running between 18- and 24-months.

τ Ability to form a 3-word sentence assessed at baseline assessment. Neurotypical children formulating 3-word sentences between 24- and 36-months.

ω GT04 was nonverbal at his baseline evaluation, indicating he had already missed the developmental milestone.

† Vineland scored between 36 and 7 days prior to AAV9-GLB1 administration.

∥ CGI-S scored between 28 and 21 days prior to AAV9-GLB1 administration.

Ω Baseline GM1 and β-galactosidase were taken from CSF between 30 and 15 days prior to AAV9-GLB1 administration. Normal β-galactosidase activity was calculated from 10 pediatric controls as described in Section C of the Supplementary Appendix.

ψ Baseline AST (upper limit of normal is 41 U/L), ALT (upper limit of normal is 30), and GGT (upper limit of normal is 16) were taken at 28 days prior to treatment.

¶ The Low Dose Cohort received 1.5×10^13^ vg/kg and the high dose cohort received 4.5×10^13^ vg/kg.

**Table 2:** Adverse Events during treatment possibly, probably, or definitely related to the gene therapy treatment.

Body System^a^ Adverse Event^b^	Low-Dose (*N*)	High-Dose (*N*)	Total (N)	Relatedness to gene therapy^c^		
				Possibly (N)	Probably (N)	Definitely (N)
**Cardiac disorders**			**1**			
Tachycardia		1	**1**	1		
**Gastrointestinal disorders**			**8**			
Vomiting		5*	**5**		3	2
Retching		1	**1**		1	
Decreased appetite		2	**2**		2	
**Laboratory abnormalities**			**21**			
Aspartate aminotransferase increased	4	5	**9**	5	3	1
Fibrin D Dimer increased	2	3	**5**		4	1
Serum ferritin increased		3	**3**		2	1
C-reactive protein increased		1	**1**			1
Alanine aminotransferase increased		2	**2**	1	1	
Positive Elispot		1	**1**			1

a Preferred Term

b Common Terminology Criteria for Adverse Events (CTCAE, v5.0)

c Based on investigator assessment

* Serious Adverse Event

## Data Availability

The data from this study is available from the corresponding author (CJT) at reasonable request upon completion of the study.
