# Supplementary material for: AAV9 Gene Therapy in GM1 Gangliosidosis Type II: A Phase 1/2 Trial
Source: medRxiv. 2025 Jul 29:2025.07.28.25332074. Preprint. [Version 1] doi: 10.1101/2025.07.28.25332074 (PMC12324653; doi:10.1101/2025.07.28.25332074)
Supplement: 1 [file NIHPP2025.07.28.25332074V1-supplement-1.pdf]

medRxiv preprint doi: https://doi.org/10.1101/2025.07.28.25332074; this version posted July 29, 2025. The copyright holder for this preprint (which was not certified by peer review) is the author/funder, who has granted medRxiv a license to display the preprint in perpetuity. This article is a US Government work. It is not subject to copyright under 17 USC 105 and is also made available for use under a CC0 license.

AAV9 Gene Therapy in GM1 Gangliosidosis Type II: A Phase 1/2 Trial

Supplementary Appendices

Table of Contents

List of Investigators……………………………………………………..………………20

Supplementary Methods……………………………………………..………………20

Supplement A: Study Design and Inclusion/Exclusion Criteria………..…………...…..21

Supplement B: Vector Design and Preparation; Plasmid Sequence……………..……..23

Supplement C: Immunosuppression Treatment Regimen……………….……………...27

Supplement D: Biochemical Monitoring……..…………………………………..…....29

Supplement E: Clinical Outcome Assessments ……………..……………....…………42

Supplement F: MRI/DTI/MRS Acquisition and Analysis……………………....………44

Supplementary Results…………..…………………………………………………...50

Supplement G: Full List of Adverse Events………..……..……………..…………..……..50

Supplement H: Clinical Laboratory Studies…..…………………....……….…….……..58

Supplement I: Viral Shedding………………….…….…………………………..……..……..65

Supplement J: Immune Response to Vector...………..…………..……………………68

Supplement K: Individualized β-galactosidase, GM1 Ganglioside, and H3N2B Levels ...73

Supplement L: Clinical Outcome Assessments……………………………...……………..78

Supplement M: Neuroimaging Results……………………..………..………...………83

Supplementary Appendences References…………….……………………………..……...89

medRxiv preprint doi: https://doi.org/10.1101/2025.07.28.25332074; this version posted July 29, 2025. The copyright holder for this preprint (which was not certified by peer review) is the author/funder, who has granted medRxiv a license to display the preprint in perpetuity. This article is a US Government work. It is not subject to copyright under 17 USC 105 and is also made available for use under a CC0 license.

List of Investigators

1 Audrey Thurm, Ph.D., Neurodevelopmental and Behavioral Phenotyping Service, National Institute of Mental Health, Bethesda, MD, USA

2 Barry J Byrne, M.D., Ph.D., Powell Gene Therapy Center, University of Florida

3 Terrence R. Flotte M.D. Ph.D., Department of Pediatrics, University of Massachusetts Chan Medical School

4 Xuntian Jiang, Ph.D., Department of Medicine, Washington University School of Medicine

5 Douglas R. Martin, Ph.D., Department of Anatomy, Physiology, & Pharmacology, Auburn, University College of Veterinary Medicine

6 Miguel Sena-Esteves Ph.D., Department of Neurology, University of Massachusetts Chan Medical School

7 Cynthia J. Tifft, M.D. Ph.D., Office of the Clinical Director and Medical Genetics Branch, National Human Genome Research Institute, National Institutes of Health

medRxiv preprint doi: https://doi.org/10.1101/2025.07.28.25332074; this version posted July 29, 2025. The copyright holder for this preprint (which was not certified by peer review) is the author/funder, who has granted medRxiv a license to display the preprint in perpetuity.
This article is a US Government work. It is not subject to copyright under 17 USC 105 and is also made available for use under a CC0 license.

Supplementary Methods

Supplement A: Study Design and Inclusion/Exclusion Criteria

The study protocol (19-HG-0101; Included with Submission) was approved by the National Institutes of Health (NIH) institutional review board and U.S. Food and Drug Administration. Written informed consent was provided by the parents or legal guardian of each patient, none of whom was deemed capable of providing informed assent. The full inclusion/exclusion criteria are included below. Cohort one (low dose) received $1.5x10^{13}$ vg/kg, and cohort two (high dose) received 4.5×10^{13} vg/kg. Twelve pediatric participants were enrolled in the dose-ranging phase of this study (six in each cohort). However, only the results of the 9 children with at least 2 years of follow-up are included in this report; 3 participants were recently enrolled and have not completed the study.

For some analyses, Type II GM1 participants were further sub-typed into late-infantile (earlier onset of symptoms; never achieved running) and juvenile (able to run by age 2).[1-3]

Inclusion Criteria

- Vineland-3 Adaptive Behavior composite standard score greater than or equal to 40
- Male or female subjects ≥ 1 day old and < 12 years old at time of full ICF signing
- Biallelic variants in *GLB1*
- Documented deficiency of β-galactosidase enzyme by clinical laboratory testing
- Phenotype consistent with a diagnosis of Type II GM1 gangliosidosis, with symptom onset after the first year of life
- AAV9 antibody titers ≤1:50
- Agree to reside within 50 miles of the study site for at least 1 month following treatment

Exclusion Criteria

- AAV9 antibody titers >1:50
- Contraindications to concomitant medications
- Serious illness that would not allow travel to the study site
- Unwilling to undergo study interventions as outlined in the Schedule of Events
- Subjects receiving other unapproved, off-label or experimental therapies for GM1 gangliosidosis (e.g., miglustat, *N*-acetyl-leucine) within the last 60 days
- Any prior participation in a study in which a gene therapy vector or stem cell transplantation was administered
- Pregnant or lactating subjects
- Immunizations of any kind in the month prior to screening
- Evidence of cardiomyopathy on history, exam, or additional testing (echocardiogram or electrocardiogram) or other cardiac disease that in the opinion of the investigator would deem the subject unsafe to participate in the trial

medRxiv preprint doi: https://doi.org/10.1101/2025.07.28.25332074; this version posted July 29, 2025. The copyright holder for this preprint (which was not certified by peer review) is the author/funder, who has granted medRxiv a license to display the preprint in perpetuity. This article is a US Government work. It is not subject to copyright under 17 USC 105 and is also made available for use under a CC0 license.

- Indwelling ferromagnetic devices that would preclude MRI/fMRI/MRS imaging
- Ongoing medical condition that is deemed by the Principal Investigator to interfere with the conduct or assessments of the study
- History of infection with human immunodeficiency virus (HIV), hepatitis A, B, or C, or tuberculosis.
- History of or current chemotherapy, radiotherapy or other immunosuppressive therapy within the past 30 days. Corticosteroid treatment may be permitted at the discretion of the PI.
- Abnormal laboratory values considered clinically significant per the investigator
- Failure to thrive, defined as falling 20 percentiles (20/100) in body weight in the 3 months preceding Screening/Baseline
- Underlying defect in immune function
- History of multiple and severe life-threatening infections

medRxiv preprint doi: https://doi.org/10.1101/2025.07.28.25332074; this version posted July 29, 2025. The copyright holder for this preprint (which was not certified by peer review) is the author/funder, who has granted medRxiv a license to display the preprint in perpetuity. This article is a US Government work. It is not subject to copyright under 17 USC 105 and is also made available for use under a CC0 license.

Supplement B: Vector Design and Preparation; Plasmid Sequence

Vector Design

As described in the protocol (attached), the AAV9-GLB1 therapy utilized in this study is a single stranded recombinant adeno-associated virus (AAV) vector encoding human lysosomal acid beta-galactosidase (Figure B1). The AAV9-GLB1 vector carries the human wild type *GLB1* open reading frame (Genbank: M34423.1) under control of the CAG promoter (CMV enhancer/chicken beta-actin promoter) followed by a polyadenylation signal derived from the simian 40 virus (SV40). The full plasmid sequence is provided below.

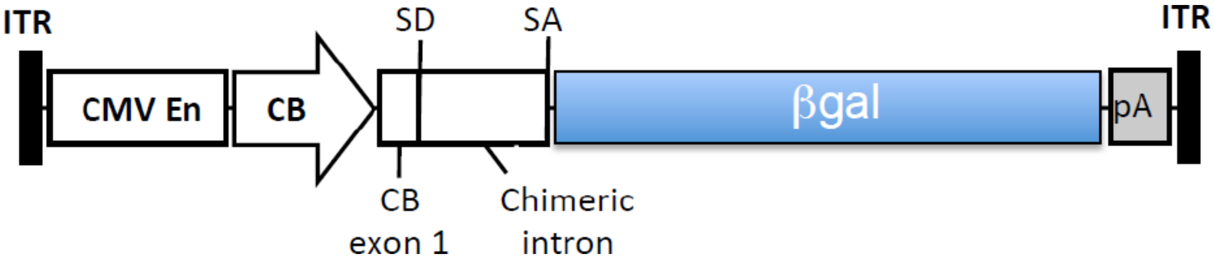

Figure B1. Schematic genome of AAV9-GLB1 vector

Vector Manufacturing

The AAV9-GLB1 vector was produced in the Clinical Manufacturing Facility at the Research Institute at Nationwide Children's Hospital according to current good manufacturing practice (cGMP). The vector was produced by triple plasmid transient transfection of adherent HEK293 cells and purified by iodixanol gradient ultracentrifugation followed by ion-exchange column chromatography. The vector was formulated in 20 mM Tris (pH 8) 1 mM $MgCl_2$, 200 mM NaCl containing 0.001% (w/v) poloxamer 188 with a final physical titer of $1.81x10^{13}$ vg/mL (digital droplet PCR), and an infectious unit titer of $3.6x10^{9}$ IU/mL ($TCID_{50}$ assay). The full:empty capsid ratio was 26:1 (96.7% full) determined by analytical ultracentrifugation. The total protein content was 212.5 µg/mL with a total host cell protein content < 8 ng/mL and residual BSA < 20 ng/mL. AAV9-GLB1 vector was stored in 2.0 mL vials at < -60°C until use.

Vector Preparation on Infusion Day

The full details of the vector preparation are described in the pharmacy manual (included in this submission). After the work surface was prepped and appropriate protective gowning and protective measures donned, the study agent was thawed at room temperature and maintained at 2-8°C until delivered to the investigator. Then using a 30- or 50-mL syringe (depending on the total vector needed to administer the patient-specific total vg) and an 18-gauge needle, the necessary volume of vector was withdrawn. The syringe was then flipped to mix the contents, the needle removed and a 0.22 µm syringe filter attached to the syringe. The filter was then connected to a sterile line attached at the other end another equally sized syringe. The contents of the first syringe were transferred to the second syringe, filtering the solution. A small-bore extension set was then attached and primed. Finally, the syringe was labeled, placed on ice, and delivered to the investigator or study team.

medRxiv preprint doi: https://doi.org/10.1101/2025.07.28.25332074; this version posted July 29, 2025. The copyright holder for this preprint (which was not certified by peer review) is the author/funder, who has granted medRxiv a license to display the preprint in perpetuity.
This article is a US Government work. It is not subject to copyright under 17 USC 105 and is also made available for use under a CC0 license.

Vector Delivery

As described in the protocol, participants received AAV9-GLB1 intravenously through a peripherally inserted central catheter (PICC) line, similar central line, or peripheral IV. The vector was administered at a rate of 1 mL/min using a standard syringe pump. The line was flushed after the vector was administered with 5 or 10 mL of saline at a rate of 1 mL/min.

Plasmid Sequence

```
LOCUS       pAAV(LysL4)-CB-hBgal (6.4) Clone 2945.4
FEATURES             Location/Qualifiers
     misc_feature    1..141
                     /note="Geneious type: LTR"
                     /standard_name="R-ITR"
     regulatory      154..646
                     /note="Geneious type: enhancer"
                     /standard_name="CMV enhancer"
     regulatory      645..946
                     /note="Geneious type: promoter"
                     /standard_name="CB promoter"
     exon            950..1042
                     /standard_name="Chicken beta-actin exon 1"
     intron          1043..1965
                     /standard_name="intron"
     exon            1966..2020
                     /standard_name="exon 2"
     CDS             2045..4078
                     /standard_name="Human GLB1 ORF"
     misc_feature    4108..4329
                     /note="Geneious type: polyA_signal"
                     /standard_name="SV40 polyA"
     misc_feature    complement(4345..4474)
                     /note="Geneious type: LTR"
                     /standard_name="L-ITR"
     misc_feature    complement(5295..6155)
                     /note="Geneious type: CDS"
                     /standard_name="Amp R"
ORIGIN
        1 cctgcaggca gctgcgcgct cgctcgctca ctgaggccgc ccgggcaaag cccgggcgtc
       61 gggcgacctt tggtcgcccg gcctcagtga gcgagcgagc gcgcagagag ggagtggcca
      121 actccatcac taggggttcc tgcggccaga tcttcaatat tggccattag ccatattatt
      181 cattggttat atagcataaa tcaatattgg ctattggcca ttgcatacgt tgtatctata
      241 tcataatatg tacatttata ttggctcatg tccaatatga ccgccatgtt ggcattgatt
      301 attgactagt tattaatagt aatcaattac ggggtcatta gttcatagcc catatatgga
      361 gttccgcgtt acataactta cggtaaatgg cccgcctggc tgaccgccca acgacccccg
      421 cccattgacg tcaataatga cgtatgttcc catagtaacg ccaataggga ctttccattg
      481 acgtcaatgg gtggagtatt tacggtaaac tgcccacttg gcagtacatc aagtgtatca
      541 tatgccaagt ccgcccccta ttgacgtcaa tgacggtaaa tggcccgcct ggcattatgc
      601 ccagtacatg accttacggg actttcctac ttggcagtac atctacgtat tagtcatcgc
      661 tattaccatg gtcgaggtga gccccacgtt ctgcttcact ctccccatct cccccccctc
      721 cccaccccca attttgtatt tatttatttt ttaattattt tgtgcagcga tgggggcggg
      781 gggggggggg gggcgcgcgc caggcggggc ggggcggggc gaggggcggg gcggggcgag
      841 gcggagaggt gcggcggcag ccaatcagag cggcgcgctc cgaaagtttc cttttatggc
      901 gaggcggcgg cggcggcggc cctataaaaa gcgaagcgcg cggcgggcgg gagtcgctgc
      961 gcgctgcctt cgccccgtgc cccgctccgc cgccgcctcg cgccgcccgc cccggctctg
     1021 actgaccgcg ttactcccac aggtgagcgg gcgggacggc ccttctcctc cgggctgtaa
```

medRxiv preprint doi: https://doi.org/10.1101/2025.07.28.25332074; this version posted July 29, 2025. The copyright holder for this preprint (which was not certified by peer review) is the author/funder, who has granted medRxiv a license to display the preprint in perpetuity. This article is a US Government work. It is not subject to copyright under 17 USC 105 and is also made available for use under a CC0 license.

```
1081 ttagcgcttg gtttaatgac ggcttgtttc ttttctgtgg ctgcgtgaaa gccttgaggg
1141 gctccgggag ggccctttgt gcggggggag cggctcgggg ggtgcgtgcg tgtgtgtgtg
1201 cgtggggagc gccgcgtgcg gctccgcgct gcccggcggc tgtgagcgct gcgggcgcgg
1261 cgcggggctt tgtgcgctcc gcagtgtgcg cgaggggagc gcggccgggg gcggtgcccc
1321 gcggtgcggg gggggctgcg aggggaacaa aggctgcgtg cggggtgtgt gcgtgggggg
1381 gtgagcaggg ggtgtgggcg cgtcggtcgg gctgcaaccc cccctgcacc cccctccccg
1441 agttgctgag cacggcccgg cttcgggtgc ggggctccgt acggggcgtg gcgcggggct
1501 cgccgtgccg ggcggggggt ggcggcaggt gggggtgccg ggcggggcgg ggccgcctcg
1561 ggccggggag ggctcggggg aggggcgcgg cggcccccgg agcgccggcg gctgtcgagg
1621 cgcggcgagc cgcagccatt gccttttatg gtaatcgtgc gagagggcgc agggacttcc
1681 tttgtcccaa atctgtgcgg agccgaaatc tgggaggcgc cgccgcaccc cctctagcgg
1741 gcgcggggcg aagcggtgcg gcgccggcag gaaggaaatg ggcggggagg gccttcgtgc
1801 gtcgccgcgc cgccgtcccc ttctccctct ccagcctcgg ggctgtccgc ggggggacgg
1861 ctgccttcgg gggggacggg gcagggcggg gttcggcttc tggcgtgtga ccggcggctc
1921 tagagcctct gctaaccatg ttcatgcctt cttctttttc ctacagctcc tgggcaacgt
1981 gctggttatt gtgctgtctc atcattttgg caaagaattc gatatcaagc ttgctagcgc
2041 caccatgccg gggttcctgg ttcgcatcct ccttctgctg ctggttctgc tgcttctggg
2101 ccctacgcgc ggcttgcgca atgccaccca gaggatgttt gaaattgact atagccggga
2161 ctccttcctc aaggatggcc agccatttcg ctacatctca ggaagcattc actactcccg
2221 tgtgccccgc ttctactgga aggaccggct gctgaagatg aagatggctg ggctgaacgc
2281 catccagacg tatgtgccct ggaactttca tgagccctgg ccaggacagt accagttttc
2341 tgaggaccat gatgtggaat attttcttcg gctggctcat gagctgggac tgctggttat
2401 cctgaggccc gggccctaca tctgtgcaga gtgggaaatg ggaggattac ctgcttggct
2461 gctagagaaa gagtctattc ttctccgctc ctccgaccca gattacctgg cagctgtgga
2521 caagtggttg ggagtccttc tgcccaagat gaagcctctc ctctatcaga atggagggcc
2581 agttataaca gtgcaggttg aaaatgaata tggcagctac tttgcctgtg attttgacta
2641 cctgcgcttc ctgcagaagc gctttcgcca ccatctgggg gatgatgtgg ttctgtttac
2701 cactgatgga gcacataaaa cattcctgaa atgtggggcc ctgcagggcc tctacaccac
2761 ggtggacttt ggaacaggca gcaacatcac agatgctttc ctaagccaga ggaagtgtga
2821 gcccaaagga cccttgatca attctgaatt ctatactggc tggctagatc actggggcca
2881 acctcactcc acaatcaaga ccgaagcagt ggcttcctcc ctctatgata tacttgcccg
2941 tggggcgagt gtgaacttgt acatgtttat aggtgggacc aattttgcct attggaatgg
3001 ggccaactca ccctatgcag cacagcccac cagctacgac tatgatgccc cactgagtga
3061 ggctggggac ctcactgaga agtattttgc tctgcgaaac atcatccaga agtttgaaaa
3121 agtaccagaa ggtcctatcc ctccatctac accaaagttt gcatatggaa aggtcacttt
3181 ggaaaagtta aagacagtgg gagcagctct ggacattctg tgtccctctg ggcccatcaa
3241 aagcctttat cccttgacat ttatccaggt gaaacagcat tatgggtttg tgctgtaccg
3301 gacaacactt cctcaagatt gcagcaaccc agcacctctc tcttcacccc tcaatggagt
3361 ccacgatcga gcatatgttg ctgtggatgg gatcccccag ggagtccttg agcgaaacaa
3421 tgtgatcact ctgaacataa cagggaaagc tggagccact ctggaccttc tggtagagaa
3481 catgggacgt gtgaactatg gtgcatatat caacgatttt aagggtttgg tttctaacct
3541 gactctcagt tccaatatcc tcacggactg gacgatcttt ccactggaca ctgaggatgc
3601 agtgcgcagc cacctggggg gctggggaca ccgtgacagt ggccaccatg atgaagcctg
3661 ggcccacaac tcatccaact acacgctccc ggccttttat atggggaact tctccattcc
3721 cagtgggatc ccagacttgc cccaggacac ctttatccag tttcctggat ggaccaaggg
3781 ccaggtctgg attaatggct ttaaccttgg ccgctattgg ccagcccggg gccctcagtt
3841 gaccttgttt gtgccccagc acatcctgat gacctcggcc ccaaacacca tcaccgtgct
3901 ggaactggag tgggcaccct gcagcagtga tgatccagaa ctatgtgctg tgacgttcgt
3961 ggacaggcca gttattggct catctgtgac ctacgatcat ccctccaaac ctgttgaaaa
4021 aagactcatg cccccacccc cgcaaaaaaa caaagattca tggctggacc atgtatgact
4081 cgagtttttt tttgcggccg cttcgagcag acatgataag atacattgat gagtttggac
4141 aaaccacaac tagaatgcag tgaaaaaaat gctttatttg tgaaatttgt gatgctattg
4201 ctttatttgt aaccattata agctgcaata aacaagttaa caacaacaat tgcattcatt
4261 ttatgtttca ggttcagggg gagatgtggg aggtttttta aagcaagtaa aacctctaca
4321 aatgtggtaa aatcgatagg ccgcaggaac ccctagtgat ggagttggcc actccctctc
4381 tgcgcgctcg ctcgctcact gaggccgggc gaccaaaggt cgcccgacgc ccgggcggcc
4441 tcagtgagcg agcgagcgcg cagctgcctg caggacatgt gagcaaaagg ccagcaaaag
```

medRxiv preprint doi: https://doi.org/10.1101/2025.07.28.25332074; this version posted July 29, 2025. The copyright holder for this preprint (which was not certified by peer review) is the author/funder, who has granted medRxiv a license to display the preprint in perpetuity.
This article is a US Government work. It is not subject to copyright under 17 USC 105 and is also made available for use under a CC0 license.

```
4501 gccaggaacc gtaaaaaggc cgcgttgctg gcgtttttcc ataggctccg cccccctgac
4561 gagcatcaca aaaatcgacg ctcaagtcag aggtggcgaa acccgacagg actataaaga
4621 taccaggcgt ttccccctgg aagctccctc gtgcgctctc ctgttccgac cctgccgctt
4681 accggatacc tgtccgcctt tctcccttcg ggaagcgtgg cgctttctca tagctcacgc
4741 tgtaggtatc tcagttcggt gtaggtcgtt cgctccaagc tgggctgtgt gcacgaaccc
4801 cccgttcagc ccgaccgctg cgccttatcc ggtaactatc gtcttgagtc caacccggta
4861 agacacgact tatcgccact ggcagcagcc actggtaaca ggattagcag agcgaggtat
4921 gtaggcggtg ctacagagtt cttgaagtgg tggcctaact acggctacac tagaagaaca
4981 gtatttggta tctgcgctct gctgaagcca gttaccttcg gaaaaagagt tggtagctct
5041 tgatccggca aacaaaccac cgctggtagc ggtggttttt ttgtttgcaa gcagcagatt
5101 acgcgcagaa aaaaaggatc tcaagaagat cctttgatct tttctacggg gtctgacgct
5161 cagtggaacg aaaactcacg ttaagggatt ttggtcatga gattatcaaa aaggatcttc
5221 acctagatcc ttttaaatta aaaatgaagt tttaaatcaa tctaaagtat atatgagtaa
5281 acttggtctg acagttacca atgcttaatc agtgaggcac ctatctcagc gatctgtcta
5341 tttcgttcat ccatagttgc ctgactcccc gtcgtgtaga taactacgat acgggagggc
5401 ttaccatctg gccccagtgc tgcaatgata ccgcgagacc cacgctcacc ggctccagat
5461 ttatcagcaa taaaccagcc agccggaagg gccgagcgca gaagtggtcc tgcaacttta
5521 tccgcctcca tccagtctat taattgttgc cgggaagcta gagtaagtag ttcgccagtt
5581 aatagtttgc gcaacgttgt tgccattgct acaggcatcg tggtgtcacg ctcgtcgttt
5641 ggtatggctt cattcagctc cggttcccaa cgatcaaggc gagttacatg atcccccatg
5701 ttgtgcaaaa aagcggttag ctccttcggt cctccgatcg ttgtcagaag taagttggcc
5761 gcagtgttat cactcatggt tatggcagca ctgcataatt ctcttactgt catgccatcc
5821 gtaagatgct tttctgtgac tggtgagtac tcaaccaagt cattctgaga atagtgtatg
5881 cggcgaccga gttgctcttg cccggcgtca atacgggata ataccgcgcc acatagcaga
5941 actttaaaag tgctcatcat tggaaaacgt tcttcggggc gaaaactctc aaggatctta
6001 ccgctgttga gatccagttc gatgtaaccc actcgtgcac ccaactgatc ttcagcatct
6061 tttactttca ccagcgtttc tgggtgagca aaaacaggaa ggcaaaatgc cgcaaaaaag
6121 ggaataaggg cgacacggaa atgttgaata ctcatactct tcctttttca atattattga
6181 agcatttatc agggttattg tctcatgagc ggatacatat ttgaatgtat ttagaaaaat
6241 aaacaaatag gggttccgcg cacatttccc cgaaaagtgc cacctgacgt ctaagaaacc
6301 attattatca tgacattaac ctataaaaat aggcgtatca cgaggccctt tcgtctcgcg
6361 cgtttcggtg atgacggtga aaacctctga cacatgcagc tcccggagac ggtcacagct
6421 tgtctgtaag cggatgccgg gagcagacaa gcccgtcagg gcgcgtcagc gggtgttggc
6481 gggtgtcggg gctggcttaa ctatgcggca tcagagcaga ttgtactgag agtgcaccat
6541 aaaattgtaa acgttaatat tttgttaaaa ttcgcgttaa atttttgtta aatcagctca
6601 ttttttaacc aatagaccga aatcggcaaa atcccttata aatcaaaaga atagcccgag
6661 atagagttga gtgttgttcc agtttggaac aagagtccac tattaaagaa cgtggactcc
6721 aacgtcaaag ggcgaaaaac cgtctatcag ggcgatggcc cactacgtga accatcaccc
6781 aaatcaagtt ttttggggtc gaggtgccgt aaagcactaa atcggaaccc taaagggagc
6841 ccccgattta gagcttgacg gggaaagccg gcgaacgtgg cgagaaagga agggaagaaa
6901 gcgaaaggag cgggcgctaa ggcgctggca agtgtagcgg tcacgctgcg cgtaaccacc
6961 acacccgccg cgcttaatgc gccgctacag ggcgcgtact atggttgctt tgacgtatgc
7021 ggtgtgaaat accgcacaga tgcgtaagga gaaaataccg catcaggcgc c
```

medRxiv preprint doi: https://doi.org/10.1101/2025.07.28.25332074; this version posted July 29, 2025. The copyright holder for this preprint (which was not certified by peer review) is the author/funder, who has granted medRxiv a license to display the preprint in perpetuity. This article is a US Government work. It is not subject to copyright under 17 USC 105 and is also made available for use under a CC0 license.

Supplement C: Immunosuppression Treatment Regimen

The immunosuppression treatment schedule for NCT03952637 is displayed in Figure C1. Participants received daily rapamycin (0.5-1 mg/m^2) beginning 3 weeks prior to AAV9-GLB1 administration until 6 months after administration; the dosage was adjusted to maintain a serum trough level of 7-12 ng/mL. Participants also received 3-4 doses (375 mg/m^2) weekly of Rituximab starting 3 weeks prior to AAV9-GLB1 administration to deplete CD20$^+$ B-cells. Participants received intravenous methylprednisolone 1 mg/kg 1-2 h prior to administration of AAV9-GLB1 gene transfer and began daily oral prednisolone 1 mg/kg starting on the first day after treatment and ending at day 4.

There were deviations in the immunosuppression treatment regimen since GT17 did not receive one of the four doses of rituximab (day -14, Table C1) following an infusion associated reaction to the first dose. In addition, following a positive Elispot, GT17 received additional daily oral prednisolone from day 14 to day 90 compared to the rest of the cohort. Lastly, five of the participants received intravenous immunoglobulin (IVIG) injections at their 6-month evaluation as clinically indicated.

AAV9-GLB1 Treatment and Monitoring Schedule

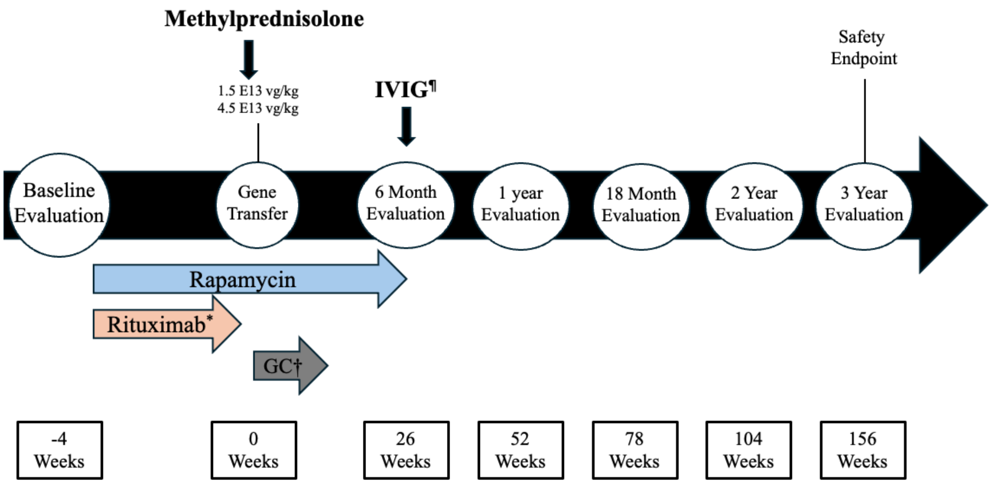

Figure C1. Immunosuppression treatment regimen and monitoring schedule. *4 Rituximab injections were given on days -21, -14, -7, and -1. GT17 did not receive a Rituximab dose on day -14 following his infusion associated reaction from the day -21 injection. ¶ Intravenous Immunoglobulin (IVIG) Injections were given for GT06, GT07, GT08, GT10, and GT11 as clinically indicated. † Oral glucocorticoids (GC) in the form of oral prednisolone were given daily starting on day one and ending after day four. GT17 received additional daily oral prednisolone from day 14 to day 90 due to a positive Elispot.

medRxiv preprint doi: https://doi.org/10.1101/2025.07.28.25332074; this version posted July 29, 2025. The copyright holder for this preprint (which was not certified by peer review) is the author/funder, who has granted medRxiv a license to display the preprint in perpetuity.
This article is a US Government work. It is not subject to copyright under 17 USC 105 and is also made available for use under a CC0 license.

Table C1. Immunosuppression Regimen

Study ID	Rituximab infusion dates (Day)	Sirolimus start date (Day)	Methylprednisolone IV	Prednisone PO	Gamunex C – Immune Globulin Inj 10% (Visit timepoint)
GT03	-21, -14, -7, -1	-21	Day 0 – One time dose	Day +1 – given daily for three days	n/a
GT04	-21, -14, -7, -1	-21	Day 0 – One time dose	Day +1 – given daily for three days	n/a
GT06	-21, -14, -7, -1	-21	Day 0 – One time dose	Day +1 – given daily for three days	Month 6 visit
GT07	-21, -14, -7, -1	-21	Day 0 – One time dose	Day +1 – given daily for three days	Month 6 visit
GT08	-21, -14, -7, -1	-21	Day 0 – One time dose	Day +1 – given daily for three days	Month 6 visit
GT10	-21, -14, -7, -1	-21	Day 0 – One time dose	Day +1 – given daily for three days	Month 6 visit
GT11	-21, -14, -7, -1	-21	Day 0 – One time dose	Day +1 – given daily for three days	Month 6 visit
GT12	-21, -14, -7, -1	-21	Day 0 – One time dose	Day +1 – given daily for three days	n/a
GT17	-21, -7, -1	-21	Day 0 – One time dose	Day +1 – given daily for three days<br><br>Then again from Day 14 to Day 90	n/a

medRxiv preprint doi: https://doi.org/10.1101/2025.07.28.25332074; this version posted July 29, 2025. The copyright holder for this preprint (which was not certified by peer review) is the author/funder, who has granted medRxiv a license to display the preprint in perpetuity. This article is a US Government work. It is not subject to copyright under 17 USC 105 and is also made available for use under a CC0 license.

Supplement D: Biochemical Monitoring

Clinical laboratory assessments were primarily performed at the National Institutes of Health (NIH) Clinical Center, and specialized laboratory tests were performed at academic and contract research organizations (see below). As described in the protocol, all participants had extensive biochemical monitoring including blood draws, lumbar punctures, and saliva, feces, and urine collections. Blood draws were completed at days -30, -21, -14, -7, -1, 0 (before gene transfer), 1, 2-3, 4-6, 7, 14, 21, 30, 45, 60, 75, 90, 6 months, 1 year, 18 months, 2 years, and 3 years (after gene transfer). Lumbar punctures were performed on participants at baseline, 90 days, 6 months, 1 year, 18 months, 2 years, and 3 years post gene transfer. Data analysis and graph generation were performed in GraphPad Prism software for macOS, version 10.1.0 (GraphPad Software).

<u>Local Clinical Laboratory Measures - NIH</u>

Serum samples were obtained at the NIH Clinical Center at days -30, -21, -14, -7, -1, 0 (before gene transfer), 1, 2-3, 4-6, 7, 14, 21, 30, 90, 6 months, 1 year, 18 months, 2 years, and 3 years. Serum samples were analyzed at local laboratories for days 45, 60, and 75. Serum was analyzed for safety including aspartate aminotransferase (AST), alanine aminotransferase levels (ALT), and Gamma glutamyl transferase (GGT), and platelet counts. Per the protocol schedule of events, safety labs were analyzed on day -30 to -15, day -14, day -7, day -1, day 2-3, day 7-8, day 14, day 21, day 30, day 45, day 60, day 75, day 90, day 180, year 1, 18 motnhs, 2 years, and 3 years relative to gene transfer. D-dimer levels were analyzed on day -30 to -15, day 1, and day 2-3 relative to gene transfer. Complement C3 and C4 were analyzed on day -30 to -15 and day 7-8. Data collection and missing data are described for each participant in Tables D1-D9.

<u>Baseline Anti-AAV9 Antibodies - ATHENA</u>

Detection of baseline Anti-AAV9 antibodies in serum was performed by ATHENA DIAGNOSTICS INC (Worcester, MA) utilizing a CLIA certified (CLIA# 22D0069726) Enzyme Linked Immunosorbent Assay (ELISA). GT04's baseline AAV9 antibody titer was performed by the University of Florida (BJB)

<u>β-galactosidase activity – Auburn University</u>

The activities of β-galactosidase in serum and cerebrospinal fluid (CSF) were measured by Auburn University (ALG) by incubating samples with 100 µL of 0.5 mM 4-methylumbelliferyl (4MU) β-D-galactoside, pH 3.8 (Sigma) in citrate-phosphate buffer (0.05 M citric acid monohydrate, 0.05 M disodium phosphate heptahydrate, 0.1 M sodium chloride) at 37°C for 1 h. Enzyme activity in the serum was determined using 10 µL of sample and activity in CSF was determined using 30 µL of sample. Enzyme activity was quenched with 3 mL of cold glycine carbonate buffer, pH 10.0 (0.17 M glycine, 0.17 M sodium carbonate, anhydrous) and fluorescence was measured with an excitation of 360 nm and emission of 450 nm on a BioTek Synergy H1 plate reader. Specific activity was expressed as nmol 4MU cleaved/mg protein/h after normalization to protein concentration as determined by the Lowry method. The controls utilized to calculate normal β-galactosidase activity consisted of 10 non-neurological participants (7 females) aged 6.35 ± 2.97 years who were enrolled in an NIH clinical protocol that allowed for use of research specimens. CSF was analyzed for β-galactosidase activity at baseline, day 90, day 180, year 1, 18 months, 2 years, and 3 years relative to gene transfer. Serum was analyzed for β-galactosidase activity at baseline, day 90, day 180, year 1, 2 years, and 3 years relative to gene transfer. GT04 did not have β-galactosidase activity analyzed for year 2 in CSF. GT17 did

medRxiv preprint doi: https://doi.org/10.1101/2025.07.28.25332074; this version posted July 29, 2025. The copyright holder for this preprint (which was not certified by peer review) is the author/funder, who has granted medRxiv a license to display the preprint in perpetuity. This article is a US Government work. It is not subject to copyright under 17 USC 105 and is also made available for use under a CC0 license.

not have β-galactosidase activity analyzed for year 2 in serum and both serum and CSF in year 3 post gene transfer.

ELISPOT/Neutralizing Antibodies - University of Massachusetts Chan Medical School

Blood samples were sent to the University of Massachusetts Chan Medical School (ALK) for ELISPOT and analysis of Neutralizing Antibodies (NAb). Serum was screened for neutralizing antibodies as previously described.[4] Luminescence was measured using a BioTek Synergy HTX reader. NAb titers are expressed as the highest dilution that inhibits β-galactosidase expression by at least 50% when compared to a negative mouse serum control. INF-y ELISpot assays were run as previously published.[4] Spot forming unit (SFU) number was determined using Mabtech IRIS reader. Responses were considered positive when the number of SFU per 1e6 cells were >50 and at least 3-fold higher than the unstimulated control condition.

GM1 Ganglioside Levels - Pharmaron

CSF samples were sent to Pharmaron Lab Services LLC (Exton, PA) for analysis of GM1 ganglioside levels. A validated liquid chromatography-tandem mass spectrometry (LC-MS/MS) method was used to determine GM1 in human CSF samples. The standard calibration curve was composed of eight standards ranging from 5 to 500 ng/mL for GM1, and the quality control (QC) samples were prepared at the Low QC (15.0 ng/mL), Mid QC (100 ng/mL), and High QC (400 ng/mL) concentrations for GM1. For each sample analysis batch, 2 replicates at each QC concentration were analyzed with two sets of standard curves. One accuracy and precision batch with four levels of QCs, lower limit of quantification QC (5.00 ng/mL), Low QC (15.0 ng/mL), Mid QC (100 ng/mL), and High QC (400 ng/mL) concentrations for GM1 was performed before initiating sample analysis to qualify the analyst and instrument used. 7 paticipants (GT04, GT06, GT07, GT08, GT03, GT10, GT11, and GT12) had CSF analyzed for GM1 ganglioside levels at baseline, day 90, day 180, year 1, 18 months, year 2, and year 3 relative to gene transfer. GT04 did not have GM1 ganglioside levels analyzed in CSF for year 3 post gene transfer. GT17, did not have CSF analyzed for GM1 ganglioside levels analyzed at 2 and 3 years post gene transfer.

Viral Shedding - Pharmaron

Sample Collection

Viral shedding sample collection in all media began with GT06. Stored urine and serum were available for GT03 and GT04 for a retrospective analysis of viral shedding. Saliva, stool, and urine samples for viral shedding analysis were collected at baseline and after intravenous gene transfer at various timepoints, such as day 0, 7, 14, 21, 30, 45, 90, 180, 365, and 18 months for most participants. Saliva and urine were collected in a sterile specimen container, and stool was collected either in a sterile specimen container or a Norgen stool nucleic acid preservation tube. All specimens were kept at optimally cold temperatures, on wet ice or kept refrigerated throughout aliquoting into storage tubes, which were all stored in a -80°C freezer. All samples were transported on dry ice to Pharmaron in insulated dry ice shipping boxes and all were received in good, frozen condition for viral shedding analysis.

Sample Analysis

The qPCR reaction (rxn) contained PrimeTime® Gene Expression Master Mix (IDT), 400 nM forward and reverse primers, 200 nM probe, and nuclease free water (IDT). Primers and probe sequences and plasmid are proprietary. 10 µL of purified matrix was assayed in a 50 µL qPCR

medRxiv preprint doi: https://doi.org/10.1101/2025.07.28.25332074; this version posted July 29, 2025. The copyright holder for this preprint (which was not certified by peer review) is the author/funder, who has granted medRxiv a license to display the preprint in perpetuity. This article is a US Government work. It is not subject to copyright under 17 USC 105 and is also made available for use under a CC0 license.

reaction. The qPCR reactions were assayed on an ABI QuantStudio 7 Pro Real-Time PCR System (ABI). The cycling parameters were as follows: 95°C for 10 min followed by 40 cycles of 95°C for 15 s and 64°C for 30 s. After cycling was complete, the plate was kept at 4°C. A standard curve made from linearized pDNA containing the target sequence (range: 25 to 1×10^7 copies/rxn) was used to quantify the amount of target sequence in the sample (copies/rxn). The number of copies/rxn quantified in the sample was multiplied by 2 to calculate viral genomes (vg)/rxn. This corrects for the quantification of the single-stranded DNA (ssDNA) viral genomes relative to the double stranded DNA (dsDNA) standard curve. The qPCR assay was qualified with an LLOQ of 50 copies/rxn (100 vg/rxn) and a ULOQ of 1×10^6 copies/rxn (2×10^6 vg/rxn).

Urine, feces, and saliva were stored at -80°C. Nucleic acids were extracted from urine, saliva, and feces using the QIAamp® 96 Virus QIAcube® HT Kit (Qiagen) and the QIAcube HT (Qiagen) automated, high-throughput nucleic acid purification system following manufacturer's instructions. The isolated nucleic acids were eluted with 130 µL of AVE buffer (Qiagen) and the final eluate was approximately 100 µL. This eluate is referred to as purified matrix. The purified matrix was stored at -80°C.

Prior to extraction, urine and feces were pretreated following the manufacture's protocol (QIAamp 96 Virus QIAcube HT Handbook, Qiagen). Saliva had no pretreatment. Urine was pretreated by combining 200 µL of urine with 15 µL of Proteinase K and 60 µL of ATL buffer (Qiagen). 200 µL of this 275 µL pretreated urine was extracted. Feces was pretreated by combining 100 µL (100 mg) of feces with 900 µL of 0.90% NaCl. The suspension was vortexed vigorously followed by centrifugation at 20K ×g for 1 min. 200 µL of the supernatant was extracted.

The conversion of copies/rxn to vg/mL of original matrix used the following equations, which account for the dilution of the sample by pretreatment and the volume (100 µL) of eluate.

Saliva: (Copies/rxn × 2 (vg correction) / 20.0 µL) x 1,000 µL/mL = vg/mL matrix. 20.0 µL of original matrix used: (200 µL saliva extracted / 100 µL of eluate) × 10 µL used in PCR.

Urine: (Detected copies × 2 (vg correction) / 14.5 µL) x 1,000 µL/mL = vg/mL matrix. 14.5 µL of original matrix used: (200 µL urine / 275 µL pretreated urine) × 200 µL pretreated urine extracted / 100 µL of eluate × 10 µL used in PCR.

Feces: (Detected copies × 2 (vg correction) / 2.00 µL) x 1,000 µL/mL = vg/mL matrix. (100 µL(mg) feces / 1000 µL pretreated feces) × 200 µL pretreated feces extracted / 100 µL of eluate × 10 µL used in PCR.

DNA Extraction, Quantification, and Quantitative PCR

DNA was extracted from serum samples collected at various time points post-dosing using a DNeasy Blood and Tissue kit (Qiagen, Valencia, CA) according to manufacturer's and in-house protocols. DNA was quantified using the NanoDrop One Microvolume UV-Vis Spectrophotometer (Thermo Fisher) according to manufacturer's and in-house protocols. Quantitative PCR (qPCR) was performed on a QuantStudio 3 (Applied Biosystems) using the QuantStudio Design and Analysis Software v1.4.1 (Applied Biosystems) according to

medRxiv preprint doi: https://doi.org/10.1101/2025.07.28.25332074; this version posted July 29, 2025. The copyright holder for this preprint (which was not certified by peer review) is the author/funder, who has granted medRxiv a license to display the preprint in perpetuity. This article is a US Government work. It is not subject to copyright under 17 USC 105 and is also made available for use under a CC0 license.

manufacturer's instructions and in-house protocols. A primer and probe set were designed to the chicken beta actin (CB) promoter that is found in the target plasmid, pAAV-CB-hβgal. Briefly, an 8-log standard curve covering a range of 1E+8 through 1E+0 copies was generated from a stock plasmid and Ct values for each standard were plotted against the log concentration of copies per sample. The linear regression of the resultant plot was utilized to calculate the copies within each sample. Each 20 µL reaction contained 13 µL of TaqPath™ ProAmp™ Master Mix (ThermoFisher Scientific), up to 0.01 µg of sample DNA, 700 nM of the forward and reverse primers, and 100 nM of FAM labeled probe. Each sample was tested in triplicate, with one replicate containing 10 copies of the target plasmid spiked in to test for inhibition of the reaction. Cycling followed manufacturer recommended guidelines, 5 min at 95 °C for initial denaturation and enzyme activation, followed by 40 cycles altering between a 15 s period at 95 °C for denaturing and a 60 s period at 60 °C for annealing and extension. Results were averaged across two replicates and normalized to vector genome copies microgram of DNA.
CB Forward:
5'-CATCTACGTATTAGTCATCGCTATTACCA-3'
CB Reverse:
5'- CCCATCGCTGCACAAAATAATTA -3'
CB Probe:
6FAM-CCACGTTCTGCTTCACTCTCCCCATC-TAMRA

H3N2B Levels – Washington University
CSF, serum, and urine collections were sent to Washington University St. Louis (XJ) to analyze H3N2b levels. As described in Pell et al.[5] liquid chromatography-tandem mass spectrometry (LC-MS/MS) was performed on all samples to determine the concentration of H3N2b utilizing validated methodology. Urine H3N2b levels were normalized to creatine levels as described in Leonard et al.[6] CSF was analyzed for H3N2b levels at baseline, day 90, day 180, year 1, 18 months, 2 years, and 3 years relative to gene transfer. Serum and urine were analyzed for H3N2b levels at baseline, day 30, day 90, day 180, year 1, 18 months, 2 years, and 3 years relative to gene transfer. 7 paticipants (GT04, GT06, GT07, GT08, GT03, GT10, GT11, and GT12) had complete H3N2b studies. GT04 did not have serum, urine, or CSF H3N2b levels analyzed in serum, CSF, or urine for year 3 following gene transfer. GT17 did not have year 2 and year 3 CSF H3N2b levels analyzed, year 3 urine H3N2b levels, or year 3 serum H3N2b levels analyzed.

IgG and IgM – University of Florida
Blood samples were sent to the University of Florida Powell Gene Therapy Center (MC, BJB), for analysis of serum IgG and IgM. The first 7 participants (GT04, GT06, GT07, GT08, GT03, GT10, and GT11) had serum analyzed for IgG and IGM analyzed at baseline (day -30), day 7, day 14, day 21, day 30, day 45, day 60, day 75, day 90, day 180, and day 365. One participant (GT12), had serum analyzed for IgG and IGM analyzed at baseline (day -30), day 7, day 14, day 21, day 30, day 45, day 60, day 75, day 90, and day 180. GT17 did not have serum analyzed for IgG or IgM.

Antibody Assay
Total anti-AAV9 IgG and IgM levels in serum were evaluated by enzyme-linked immunosorbent assays (ELISA). Serum samples from the subjects were assayed for total (neutralizing and non-neutralizing) antibodies to AAV9 capsid as previously described.[7] Briefly, 96-well plates were

medRxiv preprint doi: https://doi.org/10.1101/2025.07.28.25332074; this version posted July 29, 2025. The copyright holder for this preprint (which was not certified by peer review) is the author/funder, who has granted medRxiv a license to display the preprint in perpetuity. This article is a US Government work. It is not subject to copyright under 17 USC 105 and is also made available for use under a CC0 license.

coated with 1 x 10^9 AAV9 particles per well in sodium bicarbonate buffer, pH 8.4, overnight at 4 °C. Subsequently, the plates were washed with a solution containing phosphate-buffered saline (PBS) and 0.05% Tween-20 (PBS-T) and then blocked with 10% fetal bovine serum (FBS; Cellgro) for 2 h at 37 °C. After being washed with PBS T, the samples were serially diluted from 1:10 to 1:10,240 with a known positive human standard and allowed to bind overnight at 4 °C. The plates were washed again, followed by addition of a secondary antibody (goat anti-human IgG or IgM conjugated with horseradish peroxidase [HRP]; Invitrogen) at a dilution of 1:20,000 for 2 h at 37 °C. Finally, the plates were washed and incubated with 3,3′,5,5′-tetramethylbenzidine (TMB) peroxidase substrate (Seracare Life Sciences) in the dark. Reactions were stopped with 0.1M phosphoric acid. The reaction product was measured by spectrophotometric absorbance at 450 nm using Gen5 Microplate Reader and Imager Software (BioTek Instruments). Sample titers were calculated using the mean absorbance of up to three sequential dilutions that were within the linear region of a 4-parameter logistic standard curve generated by a known positive human standard.

Table D1. GT04 Biochemical Analysis Testing

	AST	ALT	GGT	Platelet	D-Dimer	C3	C4
Day -30	X	X	X	X			
Day -21	X	X	X	X			
Day -14	X	X	X	X			
Day -7	X	X	X	X			
Day -1	X	X	X	X			
Day 0	X	X	X		X	X	X
Day 1				X	X		
Day 2-3	X	X	X	X	X	X	X
Day 4-6	X	X	X	X	X	X	X
Day 7	X	X	X	X	X		
Day 14	X	X	X	X	X		
Day 21	X	X	X	X			
Day 30	X	X	X	X	X		
Day 45	X	X	X	X			
Day 60	X	X	X	X			
Day 75	X	X	X	X			
Day 90	X	X	X	X			
Day 180	X	X	X	X		X	X
Year 1		X		X	X		
Week 78	X	X	X	X	X	X	X
Year 2							
Year 3							

X = test completed, Red box indicates test was not completed according to protocol.

medRxiv preprint doi: https://doi.org/10.1101/2025.07.28.25332074; this version posted July 29, 2025. The copyright holder for this preprint (which was not certified by peer review) is the author/funder, who has granted medRxiv a license to display the preprint in perpetuity. This article is a US Government work. It is not subject to copyright under 17 USC 105 and is also made available for use under a CC0 license.

Table D2. GT06 Biochemical Analysis Testing

	AST	ALT	GGT	Platelet	D-Dimer	C3	C4
Day -30	X	X	X	X	X	X	X
Day -21	X	X	X	X		X	X
Day -14	X	X	X	X			
Day -7	X	X	X	X			
Day -1	X	X	X	X			
Day 0					X		
Day 1	X	X	X	X	X	X	X
Day 2-3	X	X	X	X	X	X	X
Day 4-6	X	X	X			X	X
Day 7	X	X	X	X	X	X	X
Day 14	X	X	X	X	X	X	X
Day 21	X	X	X	X	X	X	X
Day 30	X	X	X	X	X	X	X
Day 45	X	X	X	X	X	X	X
Day 60	X	X	X	X		X	X
Day 75	X	X	X	X	X	X	X
Day 90	X	X	X	X			
Day 180	X	X	X	X	X	X	X
Year 1	X	X	X	X	X	X	X
Week 78	X	X	X	X		X	X
Year 2	X	X	X	X	X		
Year 3	X	X	X	X	X	X	X

X = test completed, Red box indicates test was not completed according to protocol.

medRxiv preprint doi: https://doi.org/10.1101/2025.07.28.25332074; this version posted July 29, 2025. The copyright holder for this preprint (which was not certified by peer review) is the author/funder, who has granted medRxiv a license to display the preprint in perpetuity. This article is a US Government work. It is not subject to copyright under 17 USC 105 and is also made available for use under a CC0 license.

Table D3. GT07 Biochemical Analysis Testing

	AST	ALT	GGT	Platelet	D-Dimer	C3	C4
Day -30	X	X	X	X	X	X	X
Day -21	X	X	X	X		X	X
Day -14	X	X	X	X			
Day -7	X	X	X	X			
Day -1	X	X	X	X			
Day 0					X		
Day 1	X	X	X	X	X	X	X
Day 2-3	X	X	X	X	X	X	X
Day 4-6						X	X
Day 7	X	X	X	X	X	X	X
Day 14	X	X	X	X	X	X	X
Day 21	X	X	X	X	X	X	X
Day 30	X	X	X	X	X	X	X
Day 45	X	X	X	X	X	X	X
Day 60	X	X	X	X		X	X
Day 75	X	X	X	X	X	X	X
Day 90	X	X	X	X			
Day 180	X	X	X	X	X	X	X
Year 1	X	X	X	X	X	X	X
Week 78	X	X	X	X		X	X
Year 2	X	X	X	X	X		
Year 3	X	X	X	X	X	X	X

X = test completed, Red box indicates test was not completed according to protocol.

medRxiv preprint doi: https://doi.org/10.1101/2025.07.28.25332074; this version posted July 29, 2025. The copyright holder for this preprint (which was not certified by peer review) is the author/funder, who has granted medRxiv a license to display the preprint in perpetuity. This article is a US Government work. It is not subject to copyright under 17 USC 105 and is also made available for use under a CC0 license.

Table D4. GT08 Biochemical Analysis Testing

	AST	ALT	GGT	Platelet	D-Dimer	C3	C4
Day -30	X	X	X	X	X	X	X
Day -21	X	X	X	X			
Day -14	X	X	X	X			
Day -7	X	X	X	X			
Day -1	X	X	X	X	X		
Day 0				X	X		
Day 1	X	X	X	X	X	X	X
Day 2-3	X	X	X	X	X	X	X
Day 4-6	X	X	X			X	X
Day 7	X	X	X	X	X	X	X
Day 14	X	X	X	X	X	X	X
Day 21	X	X	X	X	X	X	X
Day 30	X	X	X	X	X	X	X
Day 45	X	X	X	X	X	X	X
Day 60	X	X	X	X		X	X
Day 75	X	X	X	X	X	X	X
Day 90	X	X	X	X			
Day 180	X	X	X	X	X	X	X
Year 1	X	X	X	X	X	X	X
Week 78	X	X	X	X		X	X
Year 2	X	X	X	X	X		
Year 3	X	X	X	X	X	X	X

X = test completed, Red box indicates test was not completed according to protocol.

medRxiv preprint doi: https://doi.org/10.1101/2025.07.28.25332074; this version posted July 29, 2025. The copyright holder for this preprint (which was not certified by peer review) is the author/funder, who has granted medRxiv a license to display the preprint in perpetuity. This article is a US Government work. It is not subject to copyright under 17 USC 105 and is also made available for use under a CC0 license.

Table D5. GT03 Biochemical Analysis Testing

	AST	ALT	GGT	Platelet	D-Dimer	C3	C4
Day -30	X	X	X	X	X	X	X
Day -21	X	X	X	X			
Day -14	X	X	X	X			
Day -7	X	X	X	X			
Day -1	X	X	X	X	X	X	X
Day 0				X			
Day 1							
Day 2-3	X	X	X	X	X		
Day 4-6	X	X	X				
Day 7	X	X	X	X	X	X	X
Day 14	X	X	X	X	X	X	X
Day 21	X	X	X	X	X	X	X
Day 30	X	X	X	X	X	X	X
Day 45	X	X	X	X			
Day 60	X	X	X	X			
Day 75	X	X	X	X			
Day 90	X	X	X	X	X	X	X
Day 180	X	X	X	X	X	X	X
Year 1	X	X	X	X	X	X	X
Week 78	X	X	X	X			
Year 2	X	X	X	X		X	X
Year 3	X	X	X	X	X	X	X

X = test completed, Red box indicates test was not completed according to protocol.

medRxiv preprint doi: https://doi.org/10.1101/2025.07.28.25332074; this version posted July 29, 2025. The copyright holder for this preprint (which was not certified by peer review) is the author/funder, who has granted medRxiv a license to display the preprint in perpetuity. This article is a US Government work. It is not subject to copyright under 17 USC 105 and is also made available for use under a CC0 license.

Table D6. GT10 Biochemical Analysis Testing

	AST	ALT	GGT	Platelet	D-Dimer	C3	C4
Day -30	X	X	X	X		X	X
Day -21	X	X	X	X			
Day -14	X	X	X	X			
Day -7	X	X	X	X			
Day -1	X	X	X	X	X	X	X
Day 0							
Day 1	X	X	X	X	X	X	X
Day 2-3	X	X	X	X	X	X	X
Day 4-6	X	X	X	X	X	X	X
Day 7	X	X	X	X	X	X	X
Day 14	X	X	X	X	X	X	X
Day 21	X	X	X	X	X	X	X
Day 30	X	X	X	X	X	X	X
Day 45	X	X	X	X	X	X	X
Day 60	X	X	X	X	X	X	X
Day 75	X	X	X	X	X	X	X
Day 90	X	X	X	X	X	X	X
Day 180	X	X	X	X	X	X	X
Year 1	X	X	X	X	X	X	X
Week 78	X	X	X	X	X		
Year 2	X	X	X	X	X	X	X
Year 3	X	X	X	X	X	X	X

X = test completed, Red box indicates test was not completed according to protocol.

medRxiv preprint doi: https://doi.org/10.1101/2025.07.28.25332074; this version posted July 29, 2025. The copyright holder for this preprint (which was not certified by peer review) is the author/funder, who has granted medRxiv a license to display the preprint in perpetuity.
This article is a US Government work. It is not subject to copyright under 17 USC 105 and is also made available for use under a CC0 license.

Table D7. GT11 Biochemical Analysis Testing

	AST	ALT	GGT	Platelet	D-Dimer	C3	C4
Day -30	X	X	X	X	X	X	X
Day -21				X			
Day -14	X	X	X	X			
Day -7	X	X	X	X		X	X
Day -1	X	X	X	X	X	X	X
Day 0				X			
Day 1	X	X	X	X	X	X	X
Day 2-3	X	X	X	X	X	X	X
Day 4-6	X	X	X	X	X	X	X
Day 7	X	X	X	X	X	X	X
Day 14	X	X	X	X	X	X	X
Day 21	X	X	X	X	X	X	X
Day 30	X	X	X	X	X	X	X
Day 45	X	X	X	X			
Day 60	X	X	X	X			
Day 75	X	X	X	X			
Day 90	X	X		X	X	X	X
Day 180	X	X	X	X	X	X	X
Year 1	X	X	X	X	X	X	X
Week 78	X	X	X	X	X		
Year 2	X	X	X	X	X	X	X
Year 3	X	X	X	X	X	X	X

X = test completed, Red box indicates test was not completed according to protocol.

medRxiv preprint doi: https://doi.org/10.1101/2025.07.28.25332074; this version posted July 29, 2025. The copyright holder for this preprint (which was not certified by peer review) is the author/funder, who has granted medRxiv a license to display the preprint in perpetuity. This article is a US Government work. It is not subject to copyright under 17 USC 105 and is also made available for use under a CC0 license.

Table D8. GT12 Biochemical Analysis Testing

	AST	ALT	GGT	Platelet	D-Dimer	C3	C4
Day -30	X	X	X	X			
Day -21				X			
Day -14	X	X	X	X			
Day -7	X	X	X	X			
Day -1							
Day 0	X	X	X	X	X	X	X
Day 1							
Day 2-3	X	X	X	X	X	X	X
Day 4-6	X	X	X	X		X	X
Day 7	X	X	X	X	X	X	X
Day 14	X	X	X	X		X	X
Day 21	X	X	X	X		X	X
Day 30	X	X	X	X	X	X	X
Day 45	X	X	X	X			
Day 60	X	X	X	X			
Day 75	X	X	X	X			
Day 90	X	X	X	X		X	X
Day 180	X	X	X	X	X	X	X
Year 1	X	X	X	X	X		
Week 78	X	X	X	X	X	X	X
Year 2	X	X	X	X	X	X	X
Year 3	X	X	X	X	X		

X = test completed, Red box indicates test was not completed according to protocol.

medRxiv preprint doi: https://doi.org/10.1101/2025.07.28.25332074; this version posted July 29, 2025. The copyright holder for this preprint (which was not certified by peer review) is the author/funder, who has granted medRxiv a license to display the preprint in perpetuity.
This article is a US Government work. It is not subject to copyright under 17 USC 105 and is also made available for use under a CC0 license.

Table D9. GT17 Biochemical Analysis Testing

	AST	ALT	GGT	Platelet	D-Dimer	C3	C4
Day -30	X	X	X	X	X	X	X
Day -21	X	X	X	X	X	X	X
Day -14							
Day -7	X	X	X	X			
Day -1	X	X	X	X	X	X	X
Day 0	X	X	X	X	X	X	X
Day 1	X	X	X	X	X	X	X
Day 2-3	X	X	X	X	X	X	X
Day 4-6	X	X	X	X	X	X	X
Day 7	X	X	X	X	X	X	X
Day 14	X	X	X	X	X	X	X
Day 21	X	X	X	X	X	X	X
Day 30	X	X	X	X	X	X	X
Day 45							
Day 60							
Day 75						X	X
Day 90	X	X	X	X	X	X	X
Day 180	X	X	X	X	X	X	X
Year 1	X	X	X	X	X	X	X
Week 78	X	X	X	X	X		
Year 2	X	X	X	X	X	X	X
Year 3							

X = test completed, Red box indicates test was not completed according to protocol.

medRxiv preprint doi: https://doi.org/10.1101/2025.07.28.25332074; this version posted July 29, 2025. The copyright holder for this preprint (which was not certified by peer review) is the author/funder, who has granted medRxiv a license to display the preprint in perpetuity. This article is a US Government work. It is not subject to copyright under 17 USC 105 and is also made available for use under a CC0 license.

Supplement E: Clinical Outcome Assessments

Vineland Adaptive Behavior Composite (ABC)

Administration of the Vineland Adaptive Behavior Scale was completed as described in D'Souza et al.[1] as a semi-structured interview with the participant's parent/caregiver. All AAV9-GLB1 participants received only the third edition of the Vineland (Vineland-3).[8] The Vineland outputs both a composite score and domain level scores (including Communication, Socialization, Daily Living, and Motor). The Vineland subdomains of interest were Receptive Communication, Expressive Communication, Fine Motor, and Gross Motor[1]. Performance was operationalized using the growth scale value (GSV), a version of the raw score that has been transformed to an interval-level scale. GSVs have conditional standard errors of measurement, allowing for the statistical evaluation of individual change,[9] and a monotonic relationship with change in the underlying construct.[10] Too few natural history data in the age range of the trial participants were available to support external comparison.

Six administrations of the Vineland were given during the main portion of the study, i.e.,: (1) between 36 and 7 days prior to AAV9-GLB1 administration; (2) at approximately 6 months post AAV9-GLB1 administration; (3) at approximately 1 year post AAV9-GLB1 administration; (4) at approximately 18 months post AAV9-GLB1 administration; (5) at approximately 2 years post AAV9-GLB1 administration; and (6) at approximately 3 years post AAV9-GLB1 administration. Here we report Vineland-3 GSV for fine motor skills, gross motor skills, receptive communication skills, and expressive communication skills.

The statistical analysis plan dictated a mixed model for repeated measures for the evaluation of change from baseline on the Vineland GSV at Year 2; the Year 3 timepoint was also included as an exploratory analysis. Fixed effects were study visit (Baseline, Month 6, Month 12, Month 18, Year 2, Year 3) and the *a priori* covariate chronological age at baseline. A random subject-level intercept and an unstructured variance-covariance matrix were used. This was an intent-to-treat analysis, so the participant with no Year 3 data was included in the analysis. The a priori hypothesis was that a significant increase from baseline in GSV score would be observed at Year 2, and alpha was set to 0.05. The mixed models were evaluated using lme4[11] and uncorrected *p*-values with Satterthwaite's approximation for degrees of freedom) were calculated using lmerTest.[12]

Clinical Global Impression (CGI)

Prospective evaluations of the Clinical Global Impression (CGI) scales were performed for each patient. The CGI scales are a brief assessment of a patient's global clinical presentation as determined from the clinician's view.[13,14] The CGI Severity (CGI-S) scale, completed at baseline only, is scored from 1 (normal) to 7 (among the most extremely ill) and assesses the patient's global severity (Table E1). The CGI Improvement (CGI-I) scale, completed at all post-infusion assessments, evaluates the participants' change in clinical presentation from the baseline evaluation and is scored from 1 (very much improved) to 7 (very much worse) with 4 corresponding to "no change" in the patient's clinical presentation (Table E2). As described in Lewis et al.,[15] CGI scores were based on a consensus reached among three researchers (PD, MTA, and CJT in this study) and were obtained within two weeks of the patient's weeklong evaluation at the NIH.

medRxiv preprint doi: https://doi.org/10.1101/2025.07.28.25332074; this version posted July 29, 2025. The copyright holder for this preprint (which was not certified by peer review) is the author/funder, who has granted medRxiv a license to display the preprint in perpetuity.
This article is a US Government work. It is not subject to copyright under 17 USC 105 and is also made available for use under a CC0 license.

CGI-S were evaluated only at the baseline evaluation for each patient. 7 evaluations of CGI-I were conducted, i.e.,: (1) at 1 month post AAV9-GLB1 administration; (2) at 3 months post AAV9-GLB1 administration; (3) at 6 month post AAV9-GLB1 administration; (4) at 1 year post AAV9-GLB1 administration; (5) at 18 months post AAV9-GLB1 administration; (6) at 2 years post AAV9-GLB1 administration, and (7) at 3 years post AAV9-GLB1 administration.

Table E1. Clinical Global Impression Severity (CGI-S)

**CGI-S Score**	**Baseline Severity**
1	Normal
2	Borderline ill
3	Midly ill
4	Moderately ill
5	Markedly ill
6	Severely ill
7	Among the most extremely ill

Table E2. Clinical Global Impression Change (CGI-I)

**CGI-I Score**	**Change Severity**
1	Very much improved
2	Much improved
3	Minimally improved
4	Normal
5	Minimally worse
6	Much worse
7	Very much worse

medRxiv preprint doi: https://doi.org/10.1101/2025.07.28.25332074; this version posted July 29, 2025. The copyright holder for this preprint (which was not certified by peer review) is the author/funder, who has granted medRxiv a license to display the preprint in perpetuity. This article is a US Government work. It is not subject to copyright under 17 USC 105 and is also made available for use under a CC0 license.

Supplement F: MRI/DTI/MRS Acquisition and Analysis

Gene therapy treated GM1 gangliosidosis participants underwent MRI studies at baseline (day -30), 1 year, 2 years, and 3 years relative to gene transfer. MRI studies included T1-weighted scans, diffusion tensor imaging (DTI), and magnetic resonance spectroscopy (MRS) studies in the same session. Changes in brain magnetic resonance imaging were assessed in relation to historical GM1 controls (see below). Volumetric analysis was performed on T1-weighted scans, differential fiber tractography was performed on DTI scans to assess changes in myelination, and MRS was analyzed for changes in *N*-acetylaspartate+*N*-acetylaspartyl glutamate (NAA), a marker of neuronal health. MRI imaging acquisition and processing are described below. Longitudinal neuroimaging data were descriptive in nature. When appropriate, data are reported as means (± SD). Data analysis and graph generation were performed in GraphPad Prism software for macOS, version 10.1.0 (GraphPad Software).

<u>NHGRI Natural History Study (NCT00029965)</u>[16]

The NIH clinical protocol, "Natural History of Glycosphingolipid Storage Disorders and Glycoprotein Disorders", encompasses a wider patient population including those with GM1 gangliosidosis, GM2 gangliosidosis, sialidosis, and galactosialidosis. Data included in this study were a subset of those from the aforementioned study, including only those with a GM1 gangliosidosis diagnosis, and a phenotype consistent with Type II disease. Diagnoses were made by documented enzyme deficiency and/or mutation analysis in a CLIA-approved laboratory.

Type II GM1 gangliosidosis participants were further sub-typed into late-infantile (earlier onset of symptoms; never achieved running) and juvenile (able to run by age 2).[1-3] All Type II GM1 gangliosidosis participants who completed a sedated MRI under the Natural History protocol were included in MRI portion of this study. Participants who were initially enrolled in the Natural History protocol (NCT00029965)[16] and later enrolled in the gene therapy treated protocol (NCT03952637)[17] were excluded from the Natural history MRI and MRS datasets. Cross-sectional evaluations were performed with the most recent MRI study for late-infantile participants with repeated MRI collection to best overlap with age of the gene therapy treated cohort. For juvenile participants, the earliest MRI collection was utilized to best overlap with the gene therapy treated cohort. 11 MRI scans from 11 late-infantile natural history participants were included in this analysis (Table F1) and 18 MRI scans from 18 juvenile natural history participants were included in this analysis (Table F2). 9 MRS scans from 9 late-infantile natural history participants were included in the MRS analysis (Table F3). 17 MRS scans from 17 juvenile natural history participants were included in the MRS analysis (Table F4). MRS scans did not always reach a sufficient signal to noise ratio, leading some scans to be discarded. For participants with multiple MRS scans, an earlier scan was used for late-infantile participants, and a later scan was used for juvenile participants given they met the signal to noise ratio cutoff.

<u>MRI Acquisition</u>

All magnetic resonance imaging, including T1-weighted, Diffusion Tensor Imaging (DTI), and Magnetic Resonance Spectroscopy (MRS), was performed on a 3-Tesla Phillips (Philips Healthcare, Best, The Netherlands) Achieva System with an 8-channel SENSE head coil.[1,18,19]

medRxiv preprint doi: https://doi.org/10.1101/2025.07.28.25332074; this version posted July 29, 2025. The copyright holder for this preprint (which was not certified by peer review) is the author/funder, who has granted medRxiv a license to display the preprint in perpetuity. This article is a US Government work. It is not subject to copyright under 17 USC 105 and is also made available for use under a CC0 license.

All MRI acquisition for both the gene therapy treated GM1 participants and untreated natural history study GM1 participants was performed under anesthesia.

T1-Weighted Acquisition and Analysis

T1- weighted images for both the gene therapy treated and NHGRI natural history participants were acquired using a 3D T1-weighted protocol with a slice thickness of 1 mm, repetition time (TR) of 11 ms, and an echo time (TE) of 7 ms, a flip angle of 6 degrees, field of view of 220 mm, and two signal averages or number of excitations (NEX). Unprocessed digital imaging and communications in medicine (DICOM) images were converted to NIfTI using *dcm2niix*.[20] T1-weighted MRI scans were then sent through volBrain's *vol2Brain* to calculate bilateral volumes of the whole brain, lateral ventricles, and thalamus.[21] As described in Lewis et al.,[22] we have previously shown that *vol2Brain* offers accurate automated volumetric segmentation of T1-weighted MRI data in this cohort. A separate simple linear regression was performed for both late-infantile and juvenile cross-sectional natural history data.

Diffusion Tensor Imaging Acquisition and Analysis

Diffusion tensor imaging (DTI) was acquired during the same session for both cohorts with the following parameters: TR/TE = 6400/100 ms, 15-gradient directions (30-directions for the Natural History protocol), b-values = 0 and 1000 s/mm^2, slice thickness = 2.5 mm, acquisition matrix = 128 × 128, NEX = 1, FOV = 24 cm. One natural history patient, GT17's untreated older sibling's (Figure 3) DTI acquisition was performed utilizing the 15-direction gene therapy protocol.

DTI were analyzed by differential tractography, a subject level analysis technique utilized to compare changes in DTI calculated metrics over time.[19,23] We used differential tractography to compare changes in fractional anisotropy (FA) in gene therapy treated GM1 participants for three years post intravenous administration of AAV9-GLB1. Differential tractography was performed as described in Lewis et al.[19] First, unprocessed DTI digital imaging and communications in medicine (DICOM) images were converted to NIfTI using *dcm2niix*.[20] DTI were then preprocessed using MRtrix3's (MRtrix, v3.0.4)[24] *dwifslpreproc*[25,26] command leveraging MRtrix3's *dwi2mask*[27] function followed by FSL's (FSL, v6.0.5)[28,29] *eddy*[25] function. Preprocessed DTI images were then imported into DSI Studio (DSI Studio, v November 6, 2023) where the DTI images were quality checked for bad slices; a U-Net mask was created to remove non-brain regions and generalized q-sampling imaging (GQI)[30] based reconstruction was performed with a diffusion sampling length ratio of 1.25.

As described in Lewis et al.,[19] a baseline whole brain tractography was performed with an angular threshold of 60 degrees, a step size of one millimeter, one million seeds, a maximum length threshold of 200 millimeters, and a minimum length threshold of 30 millimeters. The fractional anisotropy (FA) map for each patient's baseline scan was exported to a NIfTI file and utilized for differential fiber tractography. Whole brain differential tractography was calculated utilizing the same parameters as the baseline whole brain tractography using a 30 percent FA threshold. Follow-up scans were compared to the original baseline scan where fiber tract gains were determined in fiber tracts where the difference in FA between the follow-up scan and the baseline scan exceeded the FA threshold. Fiber tract losses were determined where the difference between the baseline scan and the follow-up scan exceeded the same threshold. Net fiber tract

medRxiv preprint doi: https://doi.org/10.1101/2025.07.28.25332074; this version posted July 29, 2025. The copyright holder for this preprint (which was not certified by peer review) is the author/funder, who has granted medRxiv a license to display the preprint in perpetuity. This article is a US Government work. It is not subject to copyright under 17 USC 105 and is also made available for use under a CC0 license.

metrics for the number of fiber tracts and the volume of those tracts were determined as the difference between the fiber tract gains and fiber tract losses relative to the baseline whole brain tractography as a percentage change.

Magnetic Resonance Spectroscopy Acquisition and Analysis

As described in D'Souza et al.[1] single voxel magnetic resonance spectroscopy (MRS) was performed on the left centrum semiovale (LCSO, Figure F1) during the same sedated MRI acquisition session. Voxels were graphically prescribed on the 3-D T1 weighted image in three planes. 1H-MRS was acquired with PRESS localization, CHESS water suppression, and the following parameters: TE = 38 ms, TR = 2,000 ms, and NEX = 128. A water spectrum was acquired with TE = 38 ms, TR = 5,000 ms, and NEX = 16 at the LCSO. A heavily T2 weighted image was also acquired at the LCSO with TE = 500 ms, TR = 3,000 ms, ETL = 8 to correct for CSF. A water containing phantom was placed in the field-of-view of the CSF correction image.

Post-processing was performed using LCModel,[31] followed by correcting for estimated water tissue,[32] and T1 of the metabolites in the tissue.[33] No correction was made for T2 decay of the metabolites. Based on the results from the natural history study,[5] *N*-acetylaspartate + *N*-acetylaspartyl glutamate (NAA) was analyzed for all the participants. Post-processing for CSF was performed in accordance with previously described reports.[34-36]

Reference curves were calculated using data from 38 individuals undergoing the same MRS acquisition protocol at the National Institutes of Health Clinical Center for other study protocols who were either asymptomatic or neurologically pre-symptomatic. Reference curves were generated by fitting the model . NAA concentrations for untreated and treated GM1 participants were subtracted from the age-specific reference concentration to quantify the age-appropriate difference from the reference curve. Acquisition and analysis of the MRS data was uniform between the reference data, untreated Natural History GM1 participants, and gene therapy treated GM1 gangliosidosis participants.

medRxiv preprint doi: https://doi.org/10.1101/2025.07.28.25332074; this version posted July 29, 2025. The copyright holder for this preprint (which was not certified by peer review) is the author/funder, who has granted medRxiv a license to display the preprint in perpetuity. This article is a US Government work. It is not subject to copyright under 17 USC 105 and is also made available for use under a CC0 license.

Table F1: NHGRI GM1 Natural History Study Late-Infantile GM1 Gangliosidosis T1-weighted MRI Cohort (*n* = 11).

Participant	Scan Age (Years)	Sex
NHS 13	6-10	F
NHS 24	0-5	F
NHS 35	0-5	F
NHS 63	0-5	M
NHS 72	6-10	M
NHS 73	6-10	M
NHS 84	0-5	F
NHS 85	6-10	F
NHS 22	0-5	M
NHS 23	0-5	F
NHS 30	0-5	F

Table F2: NHGRI GM1 Natural History Study Juvenile GM1 Gangliosidosis T1-weighted MRI Cohort (*n* = 18).

Participant	Scan Age (Years)	Sex
NHS 03	16-20	M
NHS 09	11-15	M
NHS 10	11-15	F
NHS 11	11-15	M
NHS 20	11-15	F
NHS 25	11-15	F
NHS 26	11-15	M
NHS 27	6-10	M
NHS 28	16-20	F
NHS 54	6-10	F
NHS 58	11-15	F
NHS 71	11-15	F
NHS 93	6-10	F
NHS 94	6-10	M
NHS 118	10-15	F
NHS 53	10-15	F
NHS 69	0-5	F
NHS 97	0-5	F

medRxiv preprint doi: https://doi.org/10.1101/2025.07.28.25332074; this version posted July 29, 2025. The copyright holder for this preprint (which was not certified by peer review) is the author/funder, who has granted medRxiv a license to display the preprint in perpetuity. This article is a US Government work. It is not subject to copyright under 17 USC 105 and is also made available for use under a CC0 license.

Table F3: NHGRI GM1 Natural History Study Late-Infantile GM1 Gangliosidosis Magnetic Resonance Spectroscopy Cohort (*n* = 9).

Participant	Scan Age (Years)	Sex
NHS 13	6-10	F
NHS 22	0-5	M
NHS 23	6-10	F
NHS 30	0-5	F
NHS 63	0-5	M
NHS 72	6-10	M
NHS 73	6-10	M
NHS 84	0-5	F
NHS 85	6-10	F

Table F4: NHGRI GM1 Natural History Study juvenile GM1 Gangliosidosis Magnetic Resonance Spectroscopy Cohort (*n* = 17).

Participant	Scan Age (Years)	Sex
NHS 03	16-20	M
NHS 09	11-15	M
NHS 10	11-15	F
NHS 11	11-15	M
NHS 20	11-15	F
NHS 25	11-15	F
NHS 26	11-15	M
NHS 27	6-10	M
NHS 28	16-20	F
NHS 54	6-10	F
NHS 58	11-15	F
NHS 64	11-15	F
NHS 69	6-10	F
NHS 71	11-15	F
NHS 82	16-20	F
NHS 93	6-10	F
NHS 94	6-10	M

medRxiv preprint doi: https://doi.org/10.1101/2025.07.28.25332074; this version posted July 29, 2025. The copyright holder for this preprint (which was not certified by peer review) is the author/funder, who has granted medRxiv a license to display the preprint in perpetuity.
This article is a US Government work. It is not subject to copyright under 17 USC 105 and is also made available for use under a CC0 license.

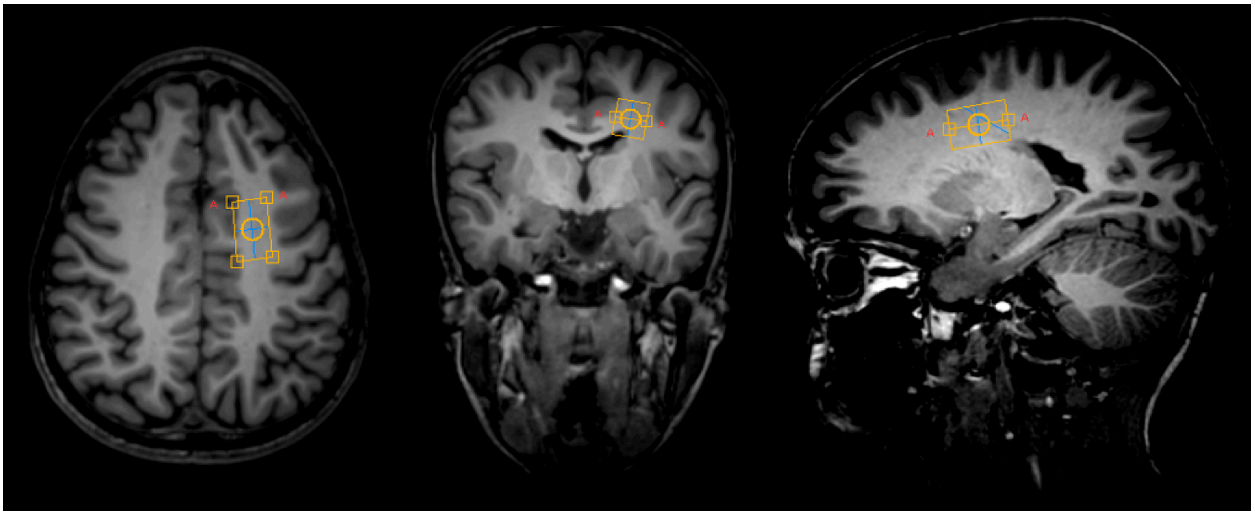

Figure F1. Magnetic resonance spectroscopy left centrum semiovale (LCSO) voxel location in a gene therapy treated GM1 patient.

Supplementary Results

Supplement G: Adverse Events

Table G1: Cumulative Summary of Serious Adverse Events

Participant	Onset	Body System[a]	AE Term[b]	Severity (CTCAE grade)[c]	AE Outcome	Causality to Study Treatment[d]	Causality to Disease Progression[d]	Causality to Immunosuppression regimen[d]	Causality to Protocol-related procedure
GT03	Day 18	Infections and infestations	Bacterial Sepsis	Grade 3 – Severe	Resolved	Not related	Not related	Not related	Definitely Related
GT04	Day 261	Neurologic	Seizures	Grade 3 Severe	Resolved	Not related	Definitely Related	Not related	Not related
GT04	Day 1081	Neurologic	Worsening Seizures	Grade 3 Severe	Not resolved	Not related	Definitely Related	Not related	Not related
GT17	Day -22	Immune system	Anaphylactic reaction	Grade 4 Life threatening	Resolved	Not related	Not related	Definitely Related	Not related
GT17	Day 3	Gastrointestinal disorders	Vomiting	Grade 3 Severe	Resolved	Definitely related[e]	Not related	Not related	Not related

[a] MedDRA Dictionary version 27.0
[b] Preferred Term
[c] Common Terminology Criteria for Adverse Events (CTCAE) version 5.0
[d] Based on Investigator assessment
[e] The event was not unexpected

medRxiv preprint doi: https://doi.org/10.1101/2025.07.28.25332074; this version posted July 29, 2025. The copyright holder for this preprint (which was not certified by peer review) is the author/funder, who has granted medRxiv a license to display the preprint in perpetuity.
This article is a US Government work. It is not subject to copyright under 17 USC 105 and is also made available for use under a CC0 license.

Table G2: Cumulative Summary of AE's in Clinical Study 19-HG-0101

Subject ID	Onset	AE Term[a]	Severity[b]	AE Outcome	Causality to Study Treatment[c]
GT03	Day -30	Skin abrasion	Grade 1 Mild	Resolved	Not related
GT03	Day -21	Elevated triglycerides	Grade 1 Mild	Resolved	Not related
GT03	Day 18	Bacterial sepsis	Grade 3 Severe	Resolved	Not related
GT03	Day 21	Iron deficiency anemia	Grade 1 Mild	Resolved	Not related
GT03	Day 93	Pyrexia	Grade 1 Mild	Resolved	Not related
GT03	Day 186	Skin laceration	Grade 2 Moderate	Resolved	Not related
GT03	Day 363	Amblyopia	Grade 2 Moderate	Not resolved	Not related
GT03	Day 363	Astigmatism	Grade 2 Moderate	Not resolved	Not related
GT03	Day 575	Presumed COVID-19 Infection	Grade 1 Mild	Resolved	Not related
GT03	Day 1095	Obstructive sleep apnea	Grade 2 Mild	Not resolved	Not related
GT04	Day -1	Platelet count decreased	Grade 1 Mild	Resolved	Not related
GT04	Day 2	Fibrin D dimer increased	Grade 1 Mild	Resolved	Probably related
GT04	Day 3	Aspartate aminotransferase increased	Grade 3 Severe	Resolved	Possibly related
GT04	Day 90	Stomatitis	Grade 2 Moderate	Resolved	Not related
GT04	Day 90	Iron deficiency anemia	Grade 1 Mild	Resolved	Not related
GT04	Day 180	Stomatitis	Grade 2 Moderate	Resolved	Not related
GT04	Day 261	Seizures	Grade 3 Severe	Resolved	Not related
GT04	Day 262	Focal seizures	Grade 3 Severe	Not resolved	Not related

medRxiv preprint doi: https://doi.org/10.1101/2025.07.28.25332074; this version posted July 29, 2025. The copyright holder for this preprint (which was not certified by peer review) is the author/funder, who has granted medRxiv a license to display the preprint in perpetuity.
This article is a US Government work. It is not subject to copyright under 17 USC 105 and is also made available for use under a CC0 license.

**Subject ID**	**Onset**	**AE Term[a]**	**Severity[b]**	**AE Outcome**	**Causality to Study Treatment[c]**
GT04	Day 1081	Worsening Seizures	Grade 3 Severe	Not resolved	Not related
GT06	Day 7	Aspartate aminotransferase increased	Grade 1 Mild	Resolved	Possibly related
GT06	Day 30	Iron deficiency anemia	Grade 1 Mild	Resolved	Not related
GT06	Day 90	Stomatitis	Grade 1 Mild	Resolved	Not related
GT06	Day 358	Drug eruption	Grade 1 Mild	Resolved	Not related
GT06	Day 605	Corona virus infection	Grade 1 Mild	Resolved	Not related
GT06	Day 672	Gastrointestinal virus	Grade 1 Mild	Resolved	Not related
GT06	Day 728	Activated partial thromboplastin time prolonged	Grade 1 Mild	Resolved	Not related
GT06	Day 734	Obstructive sleep apnea	Grade 1 Mild	Resolved	Not related
GT07	Day -7	Complication associated with device	Grade 1 Mild	Resolved	Not related
GT07	Day 15	Aspartate aminotransferase increased	Grade 1 Mild	Resolved	Possibly related
GT07	Day 30	Iron deficiency anemia	Grade 1 Mild	Resolved	Not related
GT07	Day 184	Vomiting	Grade 2 Moderate	Resolved	Not related
GT07	Day 180	Low Vitamin D	Grade 1 Mild	Resolved	Not related
GT07	Day 605	Corona virus infection	Grade 1 Mild	Resolved	Not related
GT07	Day 672	Gastrointestinal virus	Grade 1 Mild	Resolved	Not related
GT07	Day 728	Activated partial thromboplastin time prolonged	Grade 1 Mild	Resolved	Not related
GT07	Day 728	Obstructive sleep apnea	Grade 3 Severe	Resolved	Not related

medRxiv preprint doi: https://doi.org/10.1101/2025.07.28.25332074; this version posted July 29, 2025. The copyright holder for this preprint (which was not certified by peer review) is the author/funder, who has granted medRxiv a license to display the preprint in perpetuity. This article is a US Government work. It is not subject to copyright under 17 USC 105 and is also made available for use under a CC0 license.

Subject ID	Onset	AE Term[a]	Severity[b]	AE Outcome	Causality to Study Treatment[c]
GT08	Day -21	Pyrexia	Grade 1 Mild	Resolved	Not related
GT08	Day 0	Tachycardia	Grade 1 Mild	Resolved	Not related
GT08	Day 0	Hypertension	Grade 3 Severe	Resolved	Not related
GT08	Day 7	Low Vitamin D	Grade 1 Mild	Resolved	Not related
GT08	Day 15	Aspartate aminotransferase increased	Grade 1 Mild	Resolved	Possibly related
GT08	Day 31	Fibrin D dimer increased	Grade 1 Mild	Resolved	Probably related
GT08	Day 53	Stomatitis	Grade 2 Moderate	Resolved	Not related
GT08	Day 75	Iron deficiency anemia	Grade 1 Mild	Resolved	Not related
GT08	Day 88	Stomatitis	Grade 2 Moderate	Resolved	Not related
GT08	Day 605	Corona virus infection	Grade 1 Mild	Resolved	Not related
GT08	Day 672	Gastrointestinal virus	Grade 1 Mild	Resolved	Not related
GT08	Day 728	Activated partial thromboplastin time prolonged	Grade 1 Mild	Resolved	Not related
GT08	Day 728	Leukocytosis	Grade 1 Mild	Resolved	Not related
GT08	Day 728	Fibrin D dimer increased	Grade 1 Mild	Resolved	Not related
GT08	Day 735	Upper respiratory infection	Grade 1 Mild	Resolved	Not related
GT08	Day 738	Obstructive sleep apnea	Grade 3 Mild	Resolved	Not related
GT10	Day -14	Blood cholesterol increased	Grade 1 Mild	Resolved	Not related
GT10	Day -14	Neutrophil count decreased	Grade 2 Moderate	Resolved	Not related
GT10	Day -14	Hypertriglyceride mia	Grade 1 Mild	Resolved	Not related
GT10	Day 1	Tachycardia	Grade 1 Mild	Resolved	Possibly related

medRxiv preprint doi: https://doi.org/10.1101/2025.07.28.25332074; this version posted July 29, 2025. The copyright holder for this preprint (which was not certified by peer review) is the author/funder, who has granted medRxiv a license to display the preprint in perpetuity. This article is a US Government work. It is not subject to copyright under 17 USC 105 and is also made available for use under a CC0 license.

**Subject ID**	**Onset**	**AE Term[a]**	**Severity[b]**	**AE Outcome**	**Causality to Study Treatment[c]**
GT10	Day 2	Decreased appetite	Grade 2 Moderate	Resolved	Probably related
GT10	Day 4	Retching	Grade 2 Moderate	Resolved	Probably related
GT10	Day 4	Vomiting	Grade 2 Moderate	Resolved	Probably related
GT10	Day 4	Alanine aminotransferase increased	Grade 1 Mild	Resolved	Probably related
GT10	Day 7	Elevated Ferritin	Grade 1 Mild	Resolved	Probably related
GT10	Day 22	Neutrophil count decreased	Grade 3 Severe	Resolved	Not related
GT10	Day 180	Vomiting	Grade 2 Moderate	Resolved	Not related
GT10	Day 180	Pyrexia	Grade 1 Mild	Resolved	Not related
GT10	Day 365	Hypertriglyceride mia	Grade 1 Mild	Resolved	Not related
GT10	Day 635	Corona virus infection	Grade 1 Mild	Resolved	Not related
GT10	Day 1095	Hypercapnia during sleep study	Grade 1 Mild	Not resolved	Not related
GT11	Day -14	Neutrophil count decreased	Grade 3 Severe	Resolved	Not related
GT11	Day 2	Vomiting	Grade 2 Moderate	Resolved	Probably related
GT11	Day 2	Decreased appetite	Grade 2 Moderate	Resolved	Probably related
GT11	Day 2	Device occlusion	Grade 2 Moderate	Resolved	Not related
GT11	Day 3	Constipation	Grade 1 Mild	Resolved	Not related
GT11	Day 3	Aspartate aminotransferase increased	Grade 1 Mild	Resolved	Probably related
GT11	Day 5	Neutrophil count decreased	Grade 3 Severe	Resolved	Unlikely related

medRxiv preprint doi: https://doi.org/10.1101/2025.07.28.25332074; this version posted July 29, 2025. The copyright holder for this preprint (which was not certified by peer review) is the author/funder, who has granted medRxiv a license to display the preprint in perpetuity. This article is a US Government work. It is not subject to copyright under 17 USC 105 and is also made available for use under a CC0 license.

Subject ID	Onset	AE Term[a]	Severity[b]	AE Outcome	Causality to Study Treatment[c]
GT11	Day 7	Catheter site related reaction	Grade 1 Mild	Resolved	Not related
GT11	Day 7	Iron deficiency anemia	Grade 1 Mild	Resolved	Not related
GT11	Day 7	Hypogammaglobu linemia	Grade 1 Mild	Resolved	Not related
GT11	Day 30	Low Vitamin D	Grade 1 Mild	Resolved	Not related
GT11	Day 51	Aspartate aminotransferase increased	Grade 1 Mild	Resolved	Probably related
GT11	Day 63	Fibrin D dimer increased	Grade 1 Mild	Resolved	Possibly related
GT11	Day 90	Iron deficiency anemia	Grade 1 Mild	Resolved	Not related
GT11	Day 90	Neutrophil count decrease	Grade 1 Mild	Resolved	Not related
GT11	Day 180	Vomiting	Grade 1 Mild	Resolved	Not related
GT11	Day 270	Vomiting	Grade 1 Mild	Resolved	Not related
GT11	Day 309	Obstructive sleep apnea	Grade 2 Moderate	Resolved	Not related
GT11	Day 605	Corona virus infection	Grade 1 Mild	Resolved	Not related
GT12	Day -28	Headache	Grade 1 - Mild	Resolved	Not related
GT12	Day -28	Vomiting	Grade 2 - Moderate	Resolved	Not related
GT12	Day -24	Headache	Grade 1 - Mild	Resolved	Not related
GT12	Day -23	Vomiting	Grade 2 - Moderate	Resolved	Not related
GT12	Day -14	Neutrophil count decreased	Grade 2 - Moderate	Resolved	Not related
GT12	Day 0	Neutrophil count decreased	Grade 2 - Moderate	Resolved	Not related
GT12	Day 2	Fibrin D dimer increased	Grade 1 - Mild	Resolved	Probably related
GT12	Day 2	Serum ferritin increased	Grade 1 - Mild	Resolved	Probably related

medRxiv preprint doi: https://doi.org/10.1101/2025.07.28.25332074; this version posted July 29, 2025. The copyright holder for this preprint (which was not certified by peer review) is the author/funder, who has granted medRxiv a license to display the preprint in perpetuity. This article is a US Government work. It is not subject to copyright under 17 USC 105 and is also made available for use under a CC0 license.

**Subject ID**	**Onset**	**AE Term[a]**	**Severity[b]**	**AE Outcome**	**Causality to Study Treatment[c]**
GT12	Day 2	Vomiting	Grade 2 - Moderate	Resolved	Possibly related
GT12	Day 7	Aspartate aminotransferase increased	Grade 1 - Mild	Resolved	Probably related
GT12	Day 14	Neutrophil count decreased	Grade 3 - Severe	Resolved	Not related
GT12	Day 255	Corona virus infection	Grade 1 Mild	Resolved	Not related
GT12	Day 545	Upper respiratory infection	Grade 1 Mild	Resolved	Not related
GT17	Day -22	Anaphylactic reaction	Grade 4 Life threatening	Resolved	Not related
GT17	Day -20	Skin abrasion	Grade 1 Mild	Resolved	Not related
GT17	Day 0	PICC line occlusion	Grade 2 Moderate	Resolved	Not related
GT17	Day 1	Premature ventricular contractions	Grade 1 Mild	Resolved	Not related
GT17	Day 2	Vomiting	Grade 2 Moderate	Resolved	Definitely related
GT17	Day 3	Vomiting	Grade 3 Severe	Resolved	Definitely related
GT17	Day 3	Aspartate aminotransferase increased	Grade 1 Mild	Resolved	Definitely related
GT17	Day 3	Fibrin D dimer increased	Grade 1 Mild	Resolved	Definitely related
GT17	Day 3	Ferritin increased	Grade 1 Mild	Resolved	Definitely related
GT17	Day 3	C-reactive protein increased	Grade 1 Mild	Resolved	Definitely related
GT17	Day 8	PICC line flush resistance	Grade 2 Moderate	Resolved	Not related
GT17	Day 10	Gastrointestinal virus	Grade 3 Severe	Resolved	Not related
GT17	Day 14	Neutrophil count decrease	Grade 3 Severe	Resolved	Not related

medRxiv preprint doi: https://doi.org/10.1101/2025.07.28.25332074; this version posted July 29, 2025. The copyright holder for this preprint (which was not certified by peer review) is the author/funder, who has granted medRxiv a license to display the preprint in perpetuity. This article is a US Government work. It is not subject to copyright under 17 USC 105 and is also made available for use under a CC0 license.

**Subject ID**	**Onset**	**AE Term**[a]	**Severity**[b]	**AE Outcome**	**Causality to Study Treatment**[c]
GT17	Day 14	Elispot positive	Grade 2 Moderate	Resolved	Definitely related
GT17	Day 21	Hypertriglyceride mia	Grade 1 Mild	Resolved	Not related
GT17	Day 86	Aspartate aminotransferase increased	Grade 1 Mild	Resolved	Possibly related
GT17	Day 86	Neutrophil count decrease	Grade 2 Moderate	Resolved	Not related
GT17	Day 97	Neutrophil count decrease	Grade 3 Severe	Resolved	Not related
GT17	Day 97	Alanine aminotransferase increased	Grade 3 Severe	Resolved	Possibly related

[a]Common Terminology Criteria for Adverse Events (CTCAE, v5.0)
[b]Scored according to Common Terminology Criteria for Adverse Events (CTCAE) version 5.0.
[c]As determined by the principal investigator.

medRxiv preprint doi: https://doi.org/10.1101/2025.07.28.25332074; this version posted July 29, 2025. The copyright holder for this preprint (which was not certified by peer review) is the author/funder, who has granted medRxiv a license to display the preprint in perpetuity.
This article is a US Government work. It is not subject to copyright under 17 USC 105 and is also made available for use under a CC0 license.

Supplement H: Clinical Laboratory Studies

Aspartate Aminotransferase (AST)

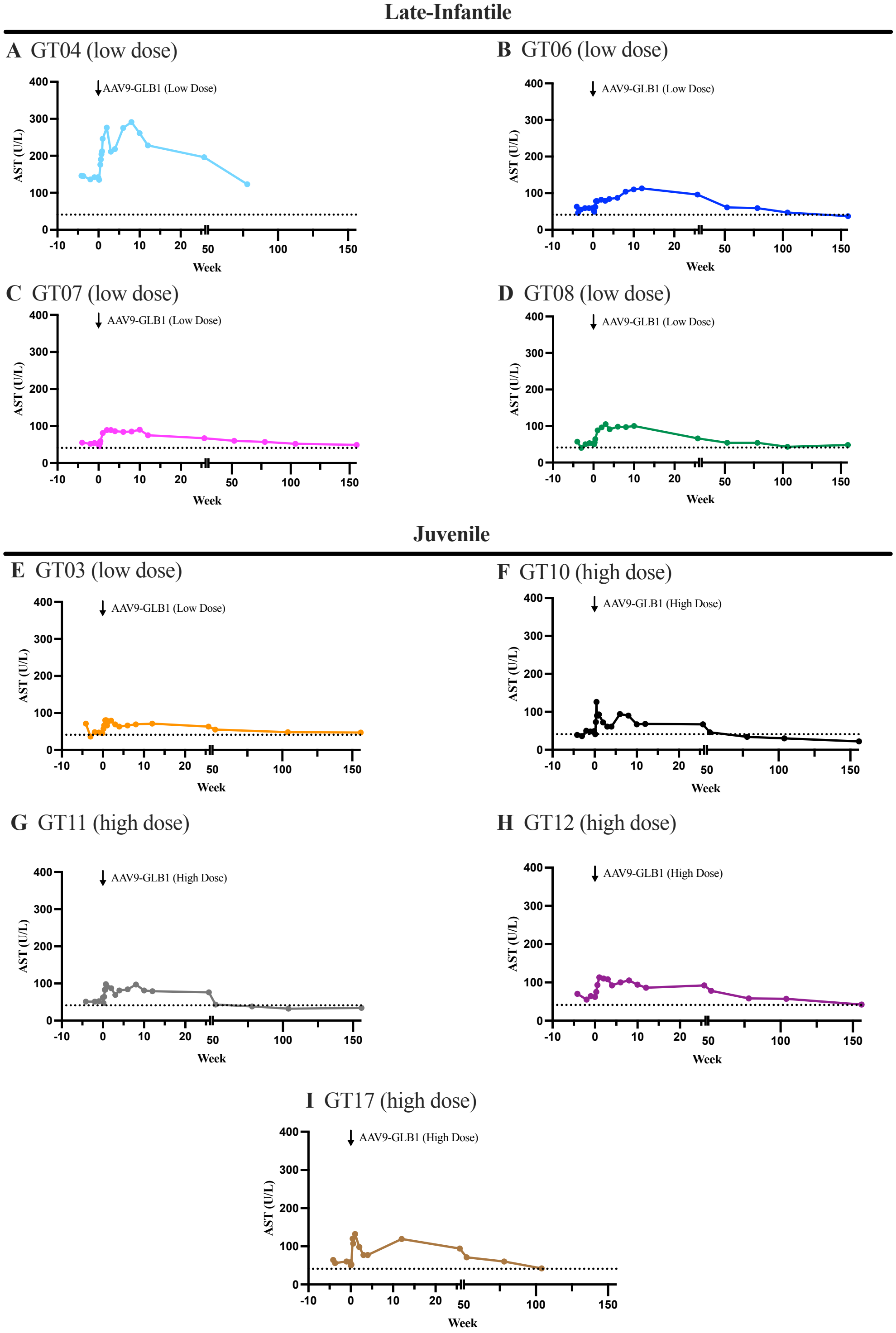

Figure H1. Aspartate aminotransferase levels (AST) in serum for each patient over the duration of the study. AAV-GLB1 gene transfer was completed on week 0 for each patient. The dotted line on each graph represents the upper limit of normal for AST (41 U/L).

medRxiv preprint doi: https://doi.org/10.1101/2025.07.28.25332074; this version posted July 29, 2025. The copyright holder for this preprint (which was not certified by peer review) is the author/funder, who has granted medRxiv a license to display the preprint in perpetuity. This article is a US Government work. It is not subject to copyright under 17 USC 105 and is also made available for use under a CC0 license.

Alanine Aminotransferase (ALT)

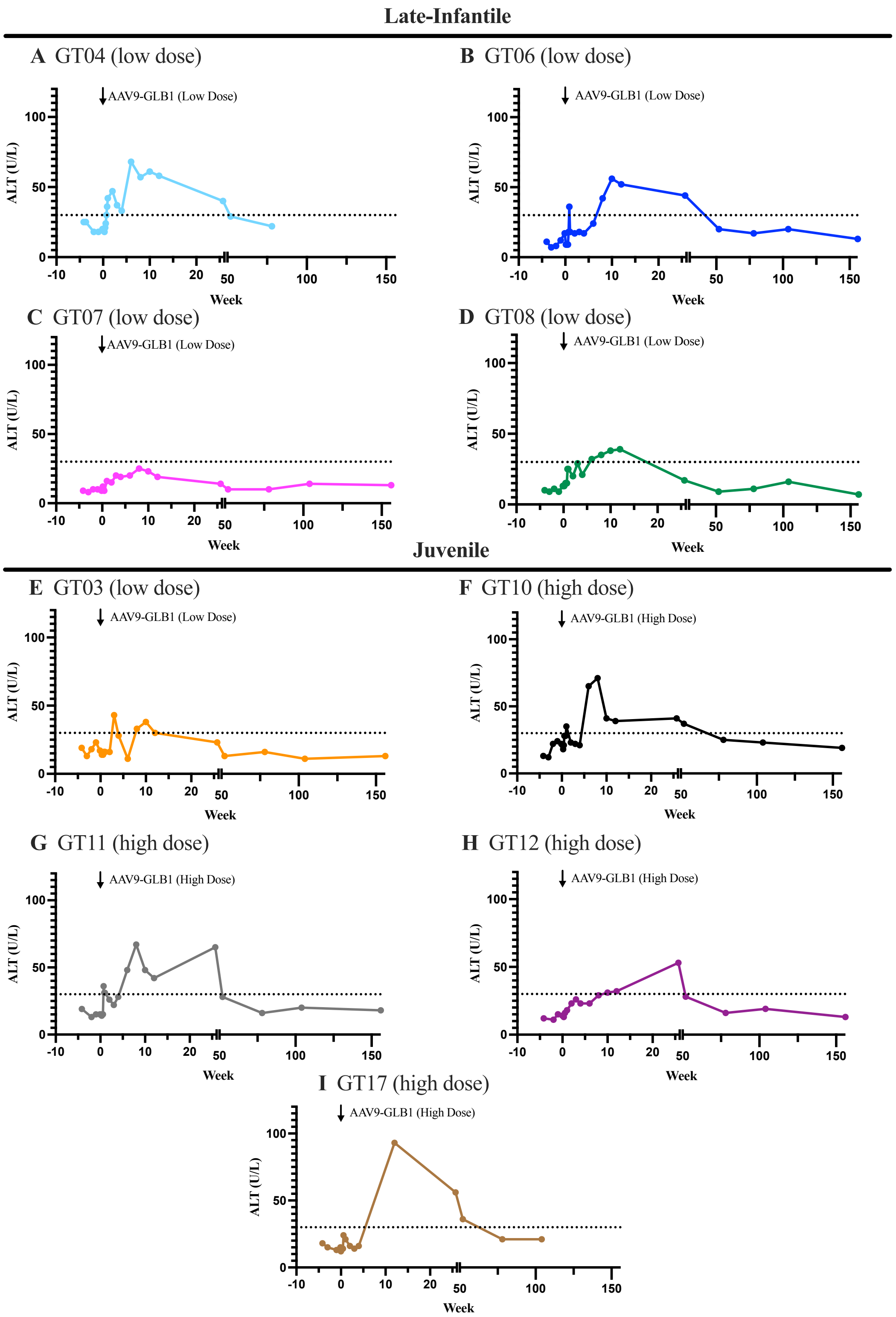

Figure H2. Alanine aminotransferase levels (ALT) in serum for each patient over the duration of the study. AAV-GLB1 gene transfer was completed on week 0 for each patient. The dotted line on each graph represents the upper limit of normal for ALT (30 U/L).

medRxiv preprint doi: https://doi.org/10.1101/2025.07.28.25332074; this version posted July 29, 2025. The copyright holder for this preprint (which was not certified by peer review) is the author/funder, who has granted medRxiv a license to display the preprint in perpetuity. This article is a US Government work. It is not subject to copyright under 17 USC 105 and is also made available for use under a CC0 license.

Gamma Glutamyl Transferase (GGT)

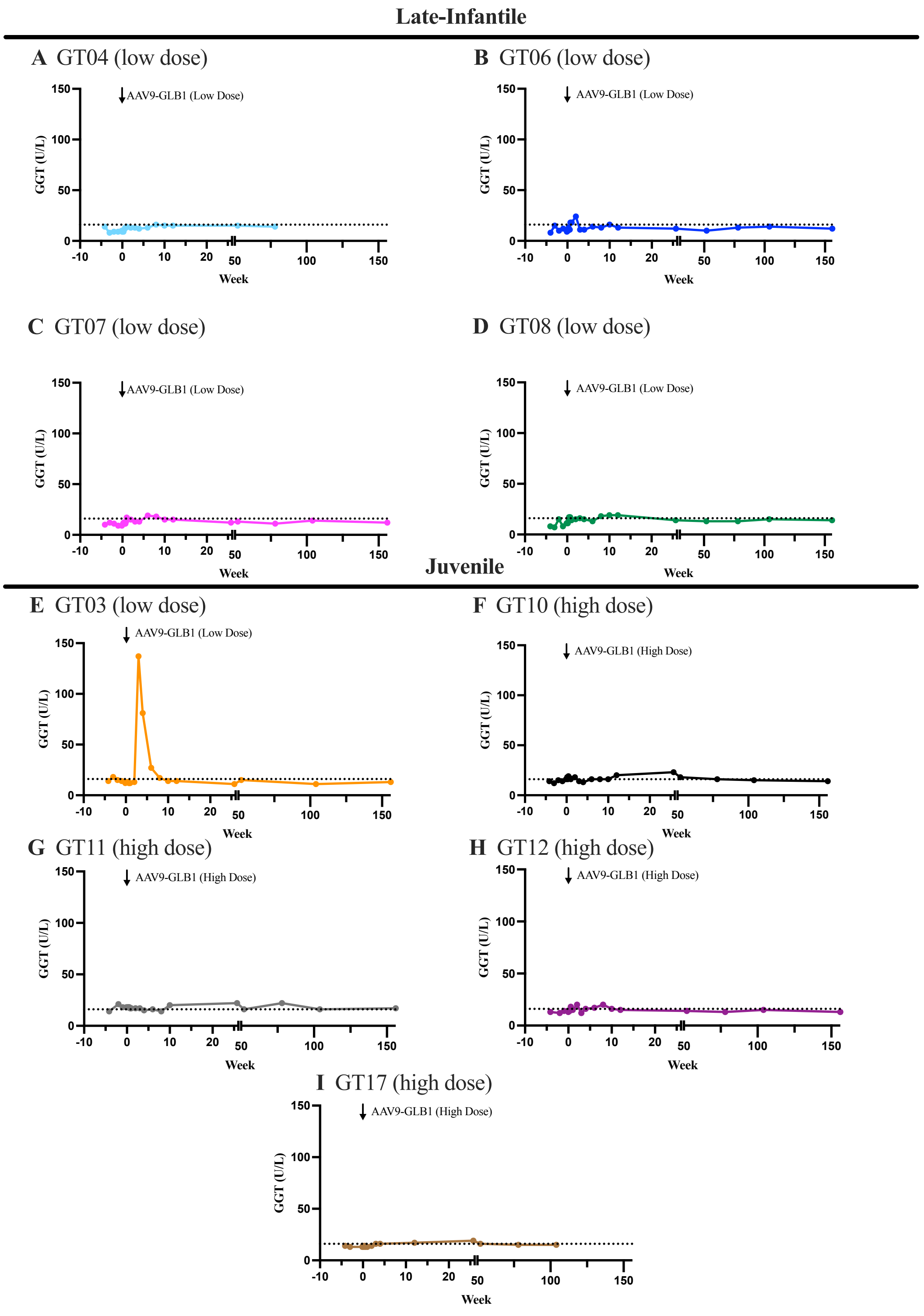

Figure H3. Gamma glutamyl transferase levels (GGT) in serum for each patient over the duration of the study. AAV-GLB1 gene transfer was completed on week 0 for each patient. The dotted line on each graph represents the upper limit of normal for GGT (16 U/L).

medRxiv preprint doi: https://doi.org/10.1101/2025.07.28.25332074; this version posted July 29, 2025. The copyright holder for this preprint (which was not certified by peer review) is the author/funder, who has granted medRxiv a license to display the preprint in perpetuity. This article is a US Government work. It is not subject to copyright under 17 USC 105 and is also made available for use under a CC0 license.

C3

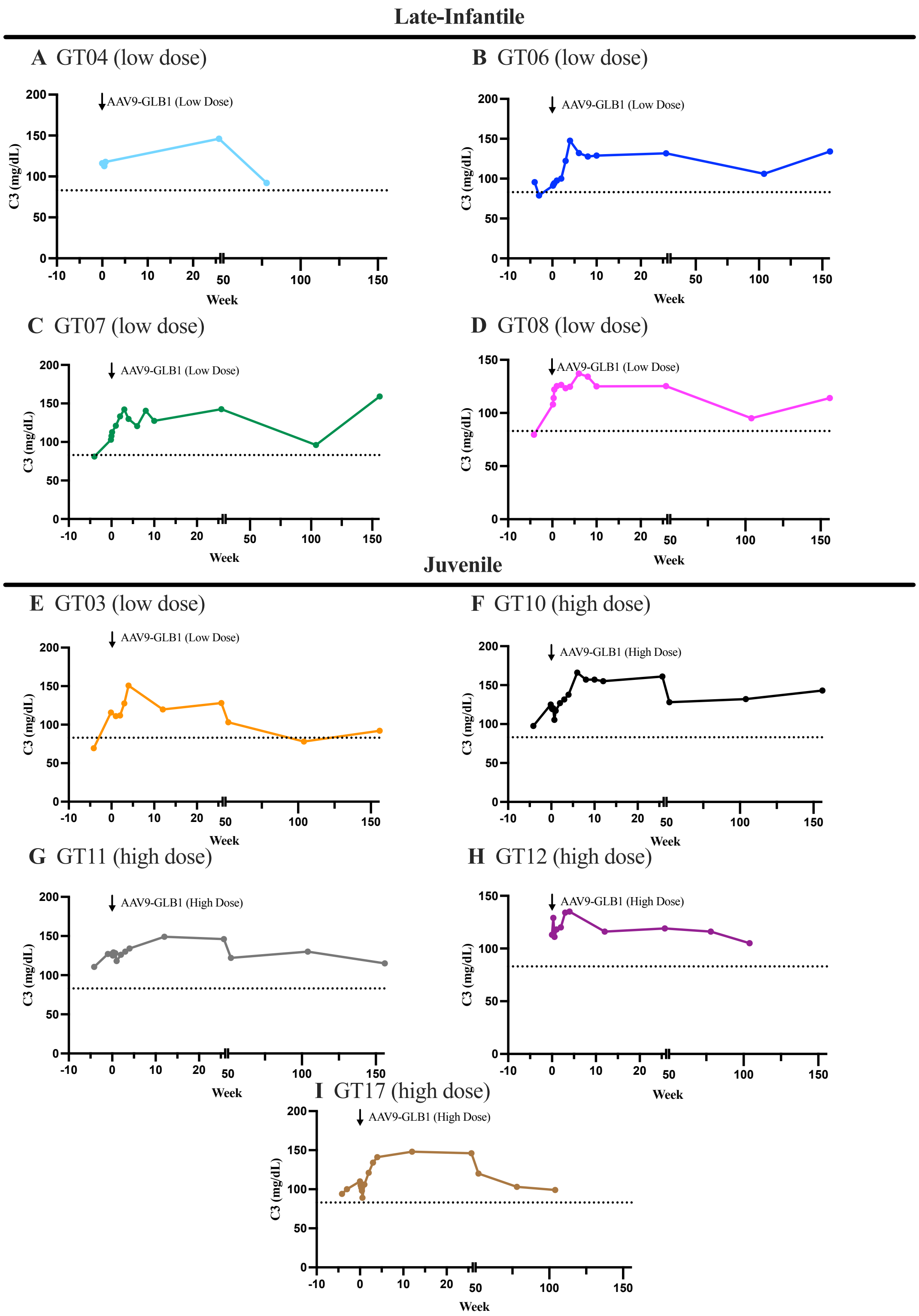

Figure H4. C3 levels in serum for each patient over the duration of the study. AAV-GLB1 gene transfer was completed on week 0 for each patient. The dotted line on each graph represents the lower limit of normal for C3 (83 mg/dL).

medRxiv preprint doi: https://doi.org/10.1101/2025.07.28.25332074; this version posted July 29, 2025. The copyright holder for this preprint (which was not certified by peer review) is the author/funder, who has granted medRxiv a license to display the preprint in perpetuity. This article is a US Government work. It is not subject to copyright under 17 USC 105 and is also made available for use under a CC0 license.

C4

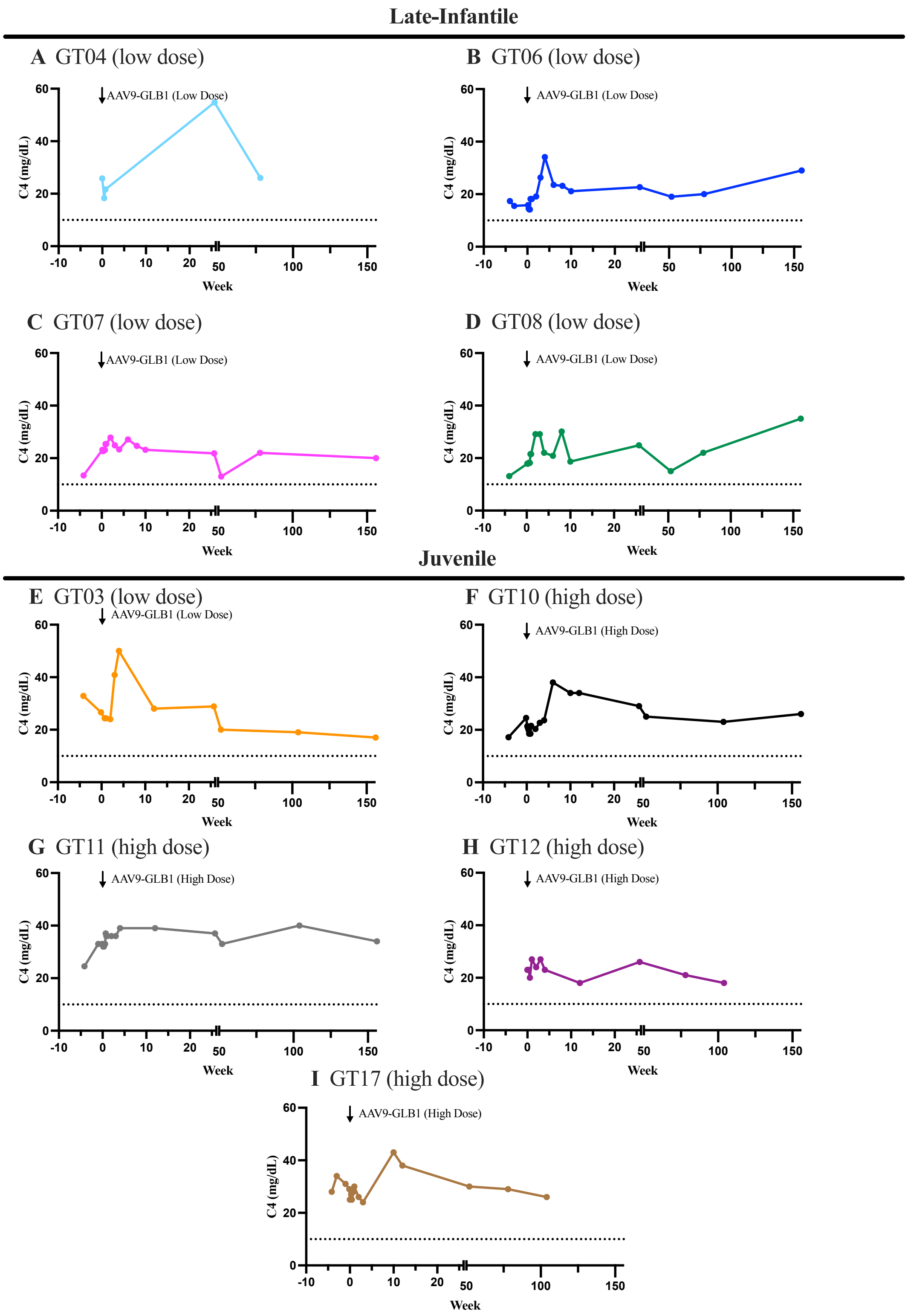

Figure H5. C4 levels in serum for each patient over the duration of the study. AAV-GLB1 gene transfer was completed on week 0 for each patient. The dotted line on each graph represents the lower limit of normal for C4 (10 mg/dL).

medRxiv preprint doi: https://doi.org/10.1101/2025.07.28.25332074; this version posted July 29, 2025. The copyright holder for this preprint (which was not certified by peer review) is the author/funder, who has granted medRxiv a license to display the preprint in perpetuity.
This article is a US Government work. It is not subject to copyright under 17 USC 105 and is also made available for use under a CC0 license.

Platelet

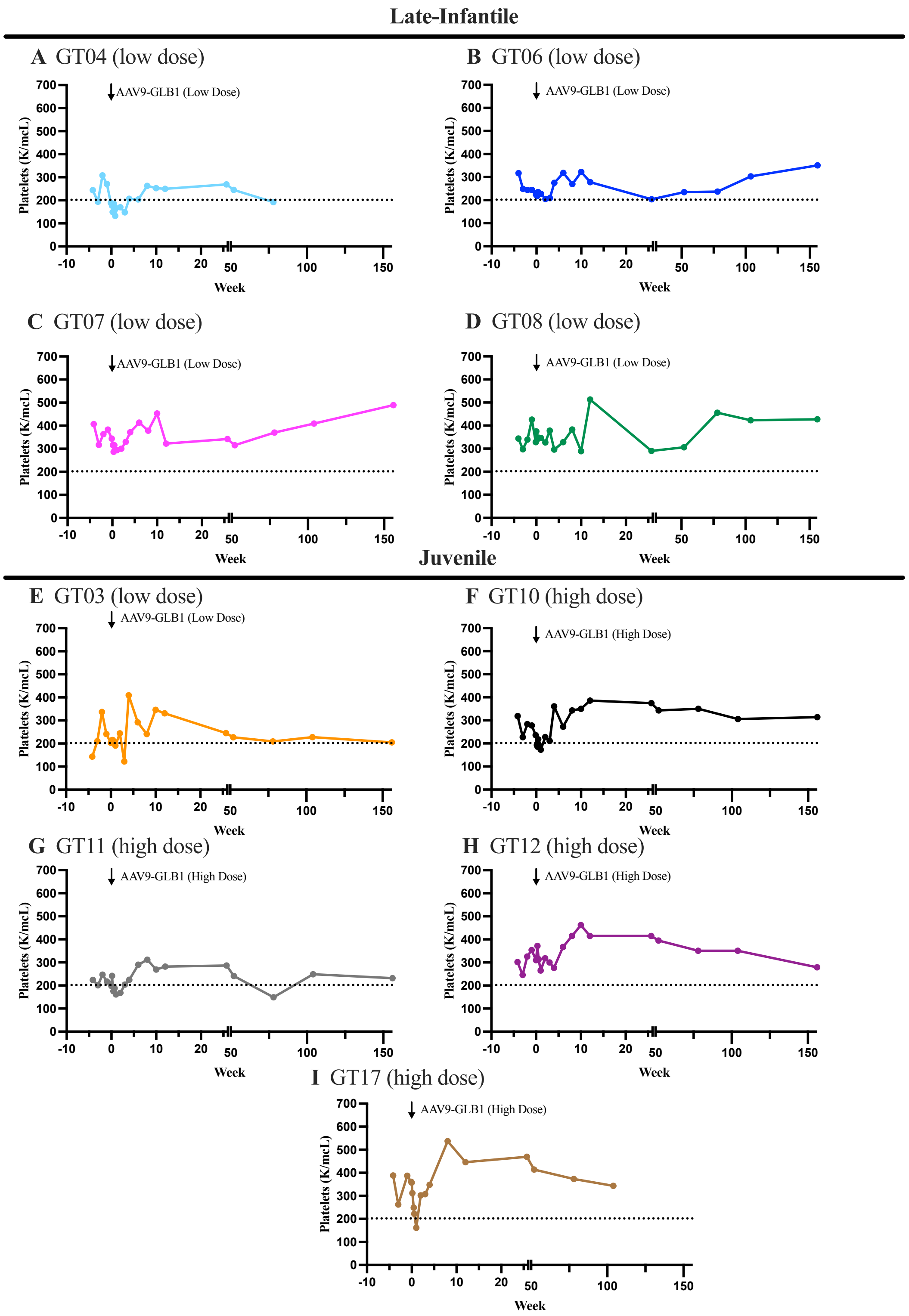

Figure H6. Platelet levels in serum for each patient over the duration of the study. AAV-GLB1 gene transfer was completed on week 0 for each patient. The dotted line on each graph represents the lower limit of normal for Platelets (202 K/mcL).

medRxiv preprint doi: https://doi.org/10.1101/2025.07.28.25332074; this version posted July 29, 2025. The copyright holder for this preprint (which was not certified by peer review) is the author/funder, who has granted medRxiv a license to display the preprint in perpetuity.
This article is a US Government work. It is not subject to copyright under 17 USC 105 and is also made available for use under a CC0 license.

D-Dimer

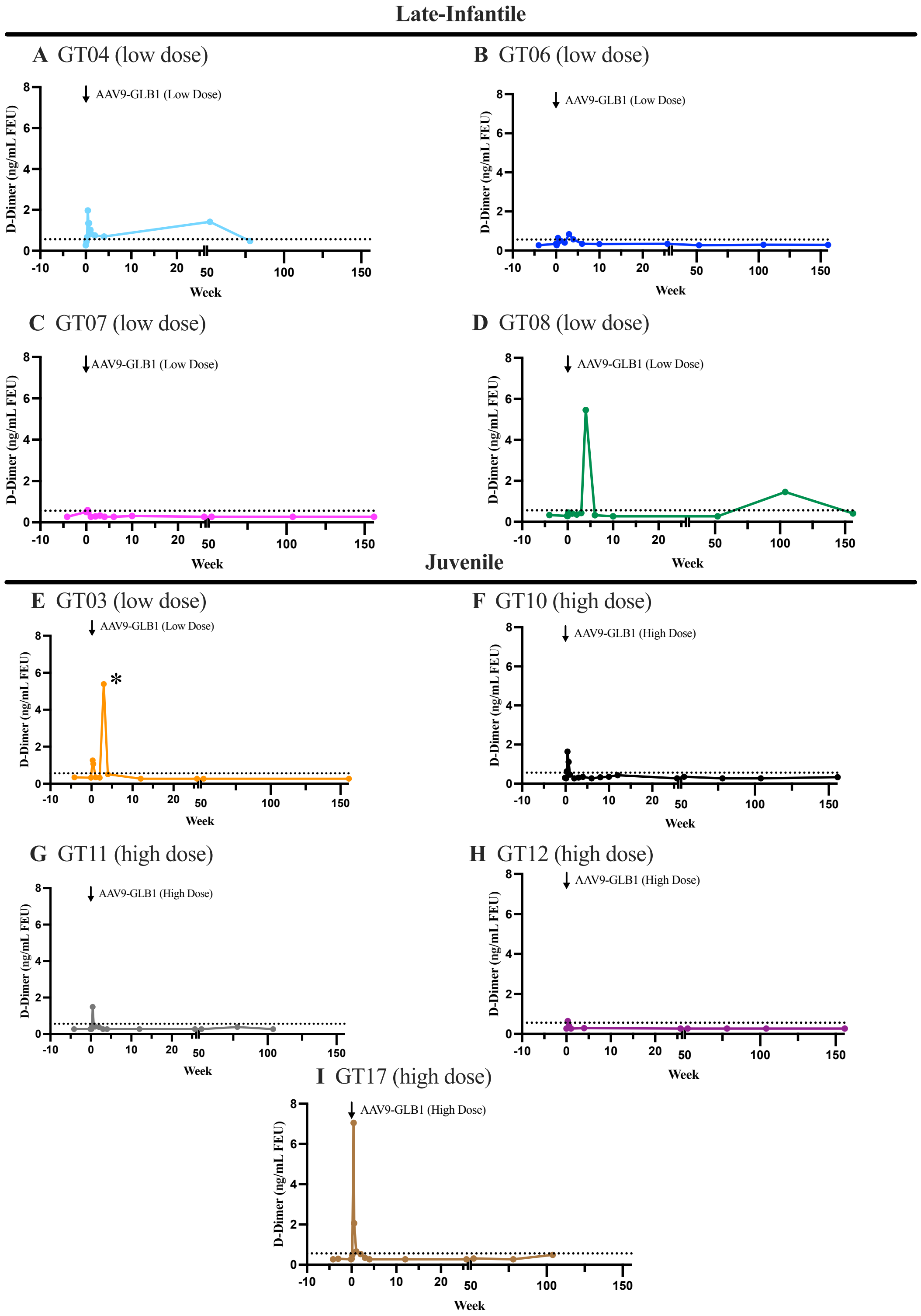

Figure H7. D-dimer levels in serum for each patient over the duration of the study. AAV-GLB1 gene transfer was completed on week 0 for each patient. The dotted line on each graph represents the upper limit of normal for D-dimer (16 U/L). The * for GT03 designates the D-dimer increase associated with central line sepsis.

medRxiv preprint doi: https://doi.org/10.1101/2025.07.28.25332074; this version posted July 29, 2025. The copyright holder for this preprint (which was not certified by peer review) is the author/funder, who has granted medRxiv a license to display the preprint in perpetuity. This article is a US Government work. It is not subject to copyright under 17 USC 105 and is also made available for use under a CC0 license.

Supplement I: Viral Shedding

The overall goal of our viral shedding studies was to determine how long affected siblings should be separated to prevent early exposure to the virus. In our studies, feces contained the highest number of vector genome copies as shown in Figure J2. Ultimately, it was found that in all three media, viral shedding was complete around day 30. This indicates that caution should be taken by both medical staff and patient families until this pointto prevent sensitizing exposure to the viral capsid.

A dose response was observed in the Urine Viral shedding analysis (Figure I1), and there was not sufficient data to visualize a dose response in Feces or Saliva (Figures I2 & I3).

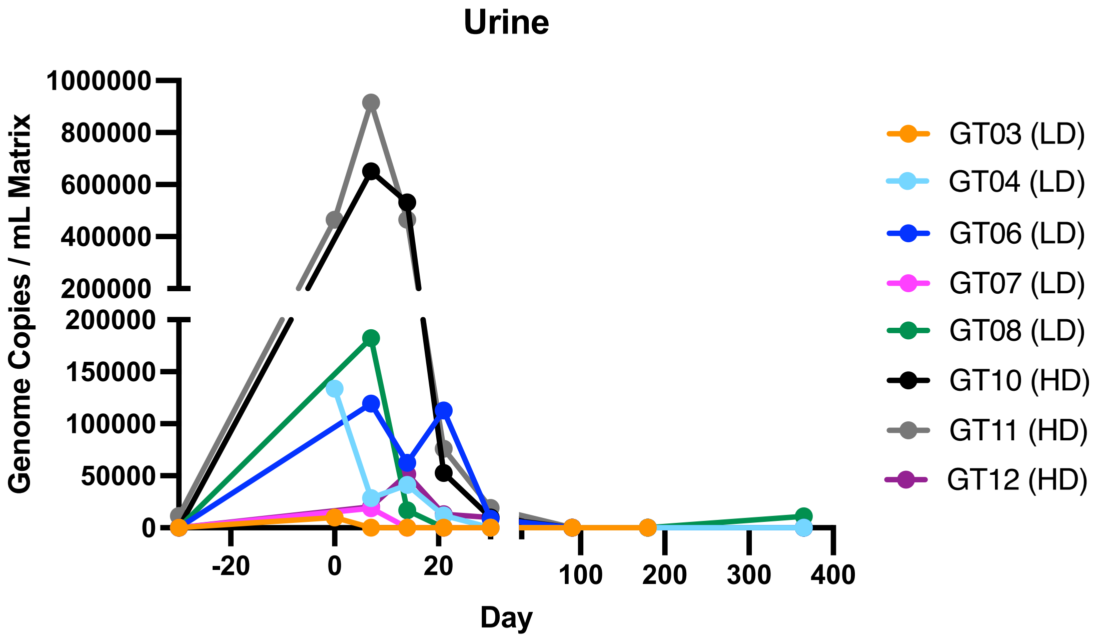

Figure I1. Viral Shedding in Urine. LD corresponds to participants who received the low dose (1.5×10^{13} vector genomes (vg) per kilogram of body weight [vg/kg]) administration of AAV9-GLB1 and HD corresponds to participants who received the high dose administration of AAV9-GLB1 (4.5×10^{13} vg/kg). Results are shown in Genome Copies per milliliter of the original matrix (Urine).

medRxiv preprint doi: https://doi.org/10.1101/2025.07.28.25332074; this version posted July 29, 2025. The copyright holder for this preprint (which was not certified by peer review) is the author/funder, who has granted medRxiv a license to display the preprint in perpetuity. This article is a US Government work. It is not subject to copyright under 17 USC 105 and is also made available for use under a CC0 license.

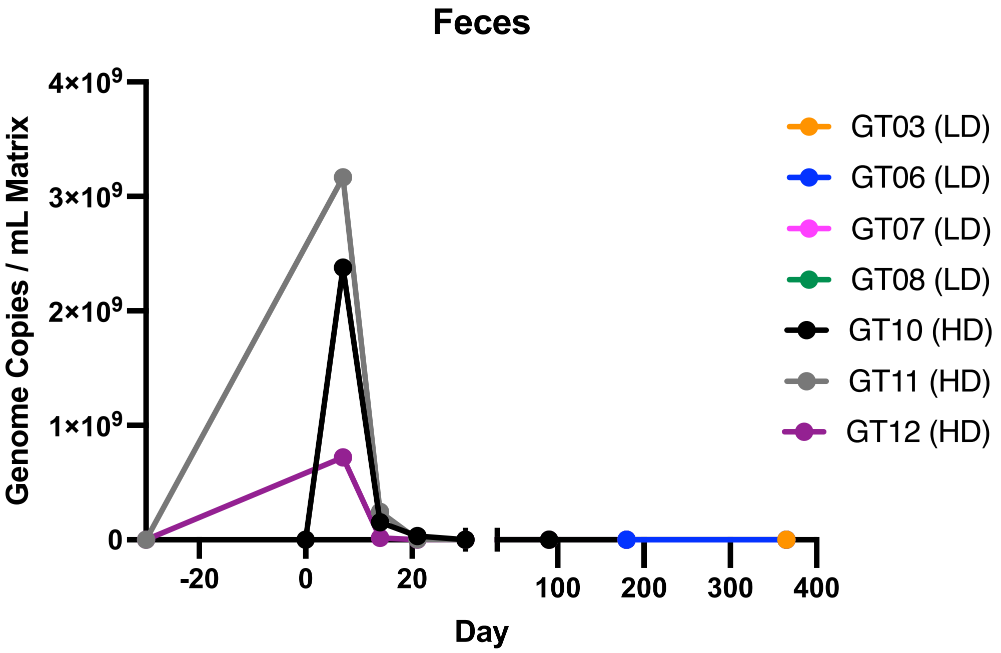

Figure I2. Viral Shedding in Feces. LD corresponds to participants who received the low dose (1.5×10^{13} vector genomes (vg) per kilogram of body weight [vg/kg]) administration of AAV9-GLB1 and HD corresponds to participants who received the high dose administration of AAV9-GLB1 (4.5×10^{13} vg/kg). Results are shown in Genome Copies per milliliter of the original matrix (Feces).

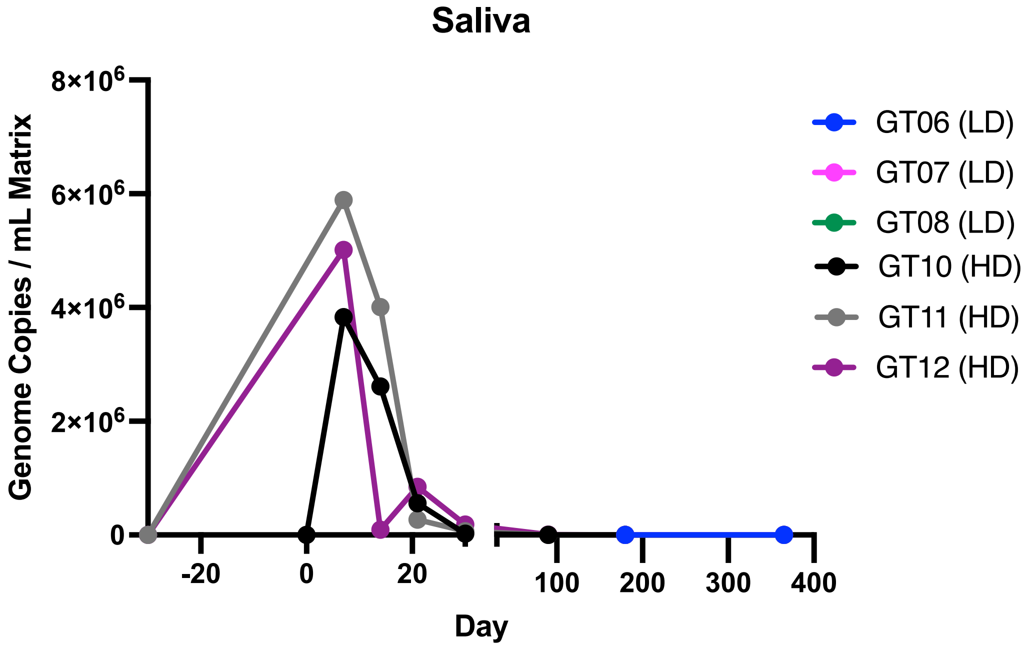

Figure I3. Viral Shedding in Saliva. LD corresponds to participants who received the low dose (1.5×10^{13} vector genomes (vg) per kilogram of body weight [vg/kg]) administration of AAV9-GLB1 and HD corresponds to participants who received the high dose administration of AAV9-

medRxiv preprint doi: https://doi.org/10.1101/2025.07.28.25332074; this version posted July 29, 2025. The copyright holder for this preprint
(which was not certified by peer review) is the author/funder, who has granted medRxiv a license to display the preprint in perpetuity.
This article is a US Government work. It is not subject to copyright under 17 USC 105 and is also made available
for use under a CC0 license.

GLB1 (4.5×10^{13} vg/kg). Results are shown in Genome Copies per milliliter of the original matrix (Saliva).

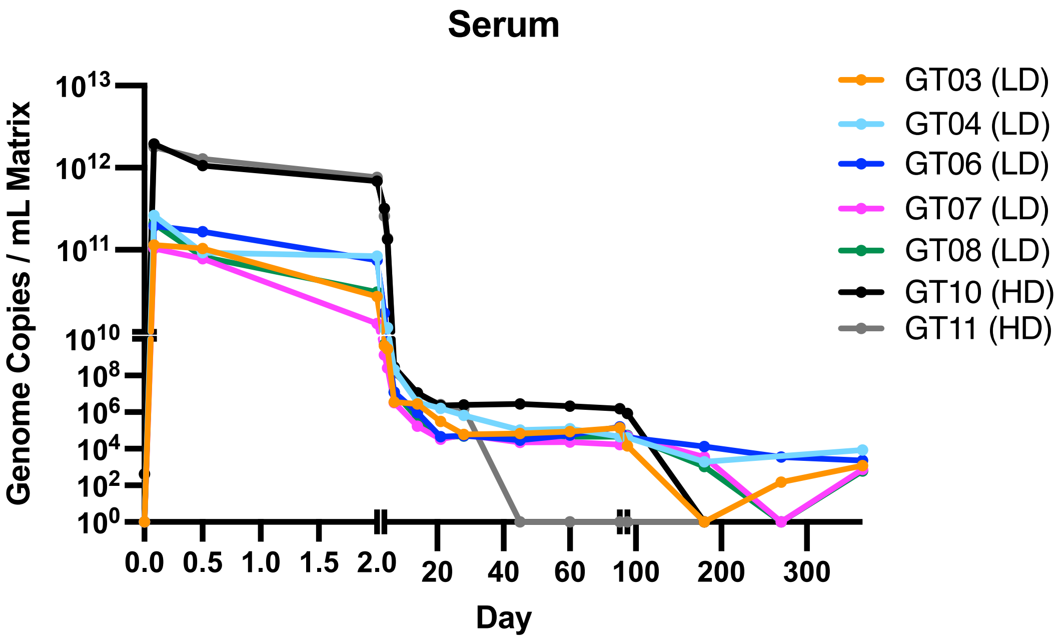

Figure I4. Viral Shedding in Serum. LD corresponds to participants who received the low dose (1.5×10^{13} vector genomes (vg) per kilogram of body weight [vg/kg]) administration of AAV9-GLB1 and HD corresponds to participants who received the high dose administration of AAV9-GLB1 (4.5×10^{13} vg/kg). Results are shown in Genome Copies per milliliter of the original matrix (Serum).

medRxiv preprint doi: https://doi.org/10.1101/2025.07.28.25332074; this version posted July 29, 2025. The copyright holder for this preprint (which was not certified by peer review) is the author/funder, who has granted medRxiv a license to display the preprint in perpetuity. This article is a US Government work. It is not subject to copyright under 17 USC 105 and is also made available for use under a CC0 license.

Supplement J: Immune Response to the Vector

Anti-AAV9 IgG

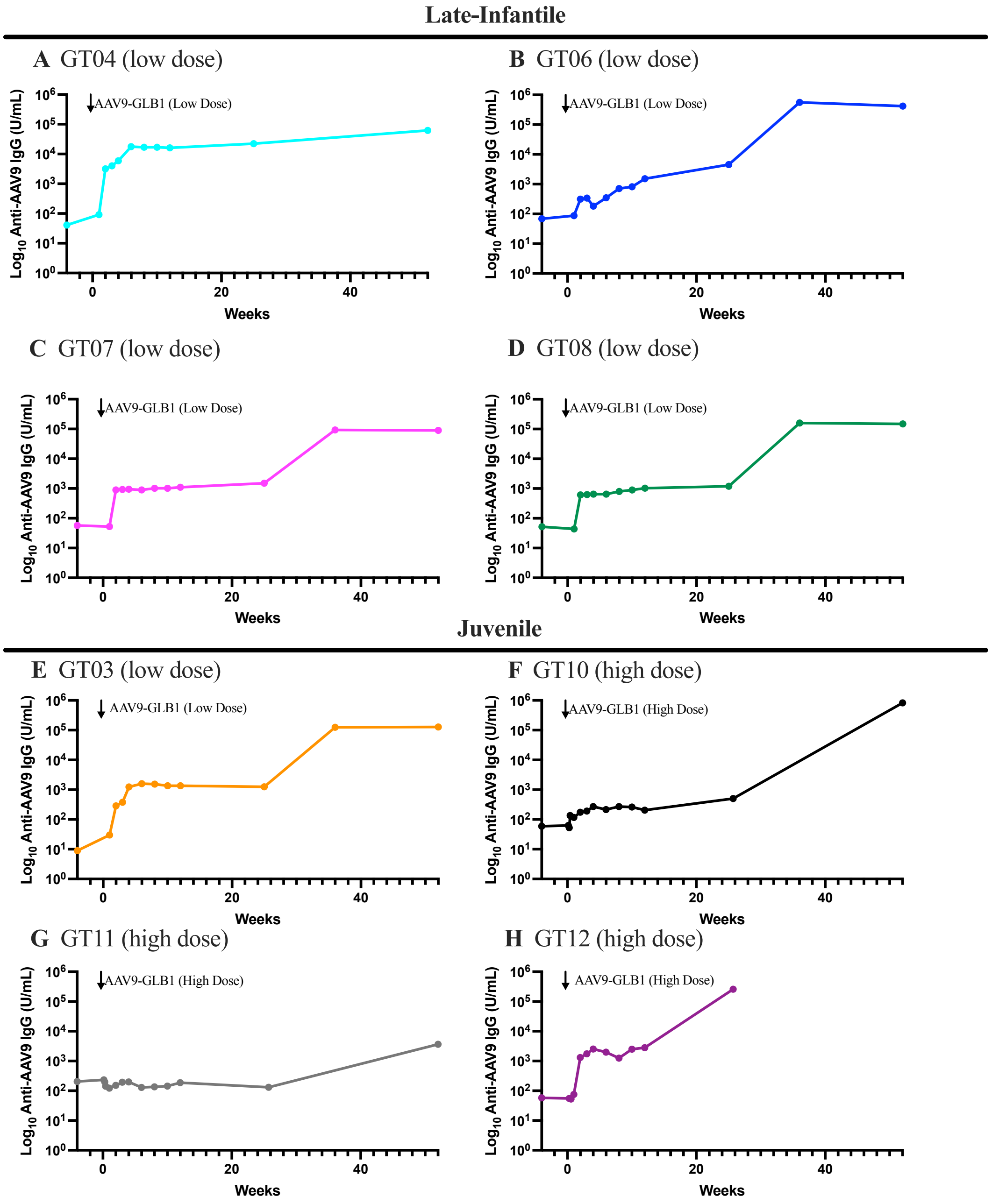

Figure J1. Anti-AAV9 IgG levels in serum.

medRxiv preprint doi: https://doi.org/10.1101/2025.07.28.25332074; this version posted July 29, 2025. The copyright holder for this preprint (which was not certified by peer review) is the author/funder, who has granted medRxiv a license to display the preprint in perpetuity.
This article is a US Government work. It is not subject to copyright under 17 USC 105 and is also made available for use under a CC0 license.

Anti-AAV9 IgM

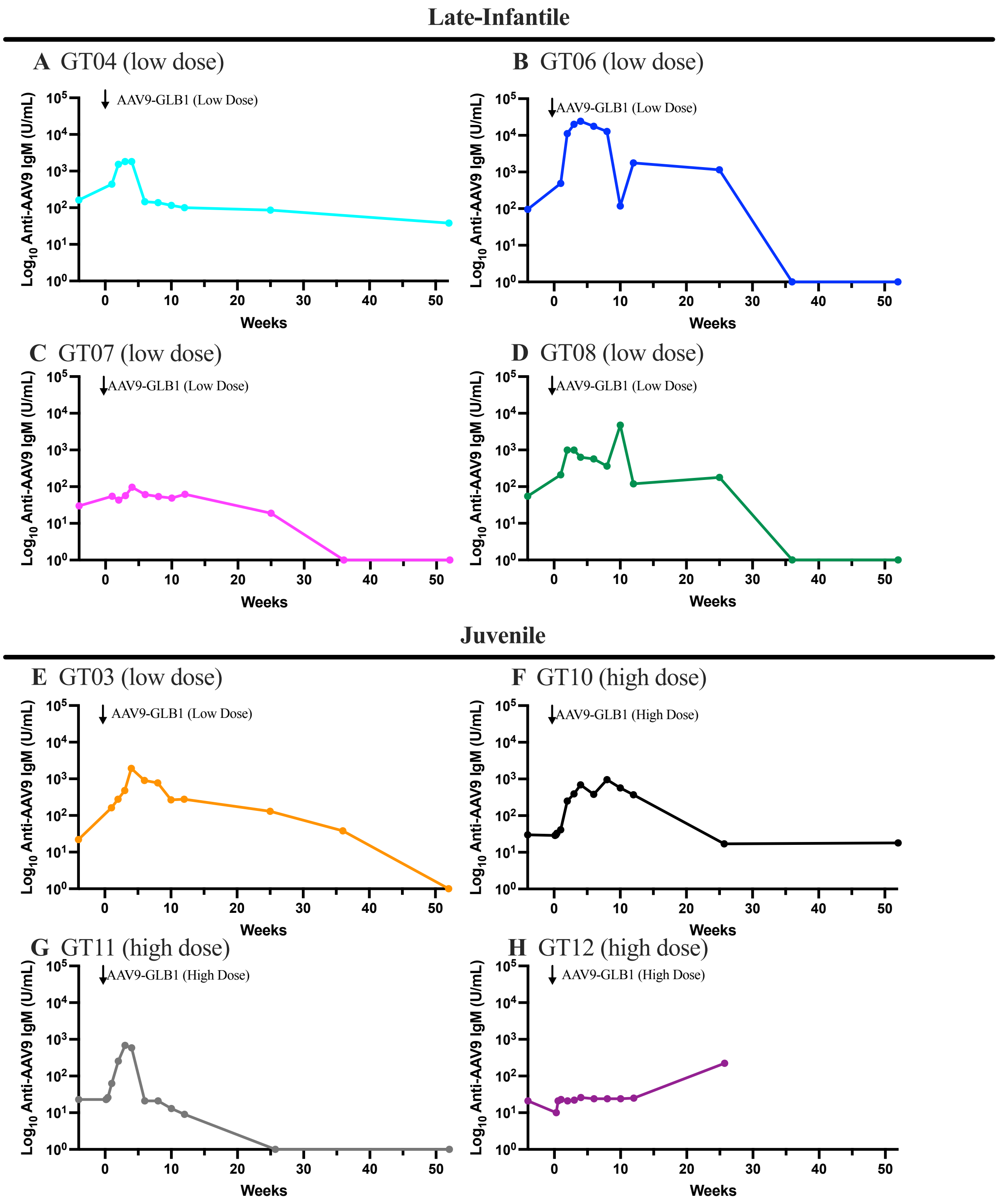

Figure J2. Anti-AAV9 IgM levels in serum.

medRxiv preprint doi: https://doi.org/10.1101/2025.07.28.25332074; this version posted July 29, 2025. The copyright holder for this preprint (which was not certified by peer review) is the author/funder, who has granted medRxiv a license to display the preprint in perpetuity.
This article is a US Government work. It is not subject to copyright under 17 USC 105 and is also made available for use under a CC0 license.

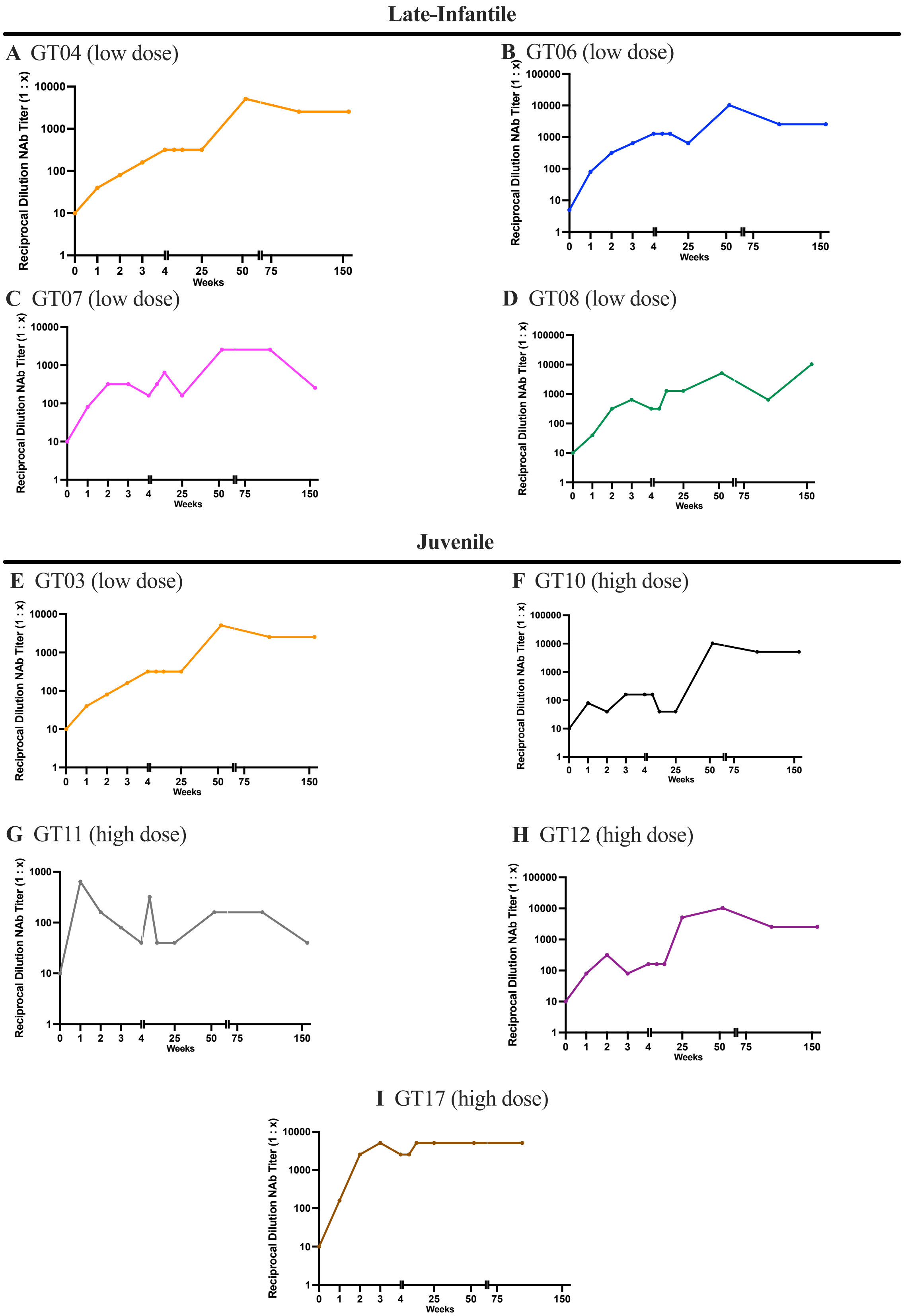

Figure J3. Neutralizing antibodies for all participants over time.

medRxiv preprint doi: https://doi.org/10.1101/2025.07.28.25332074; this version posted July 29, 2025. The copyright holder for this preprint (which was not certified by peer review) is the author/funder, who has granted medRxiv a license to display the preprint in perpetuity.
This article is a US Government work. It is not subject to copyright under 17 USC 105 and is also made available for use under a CC0 license.

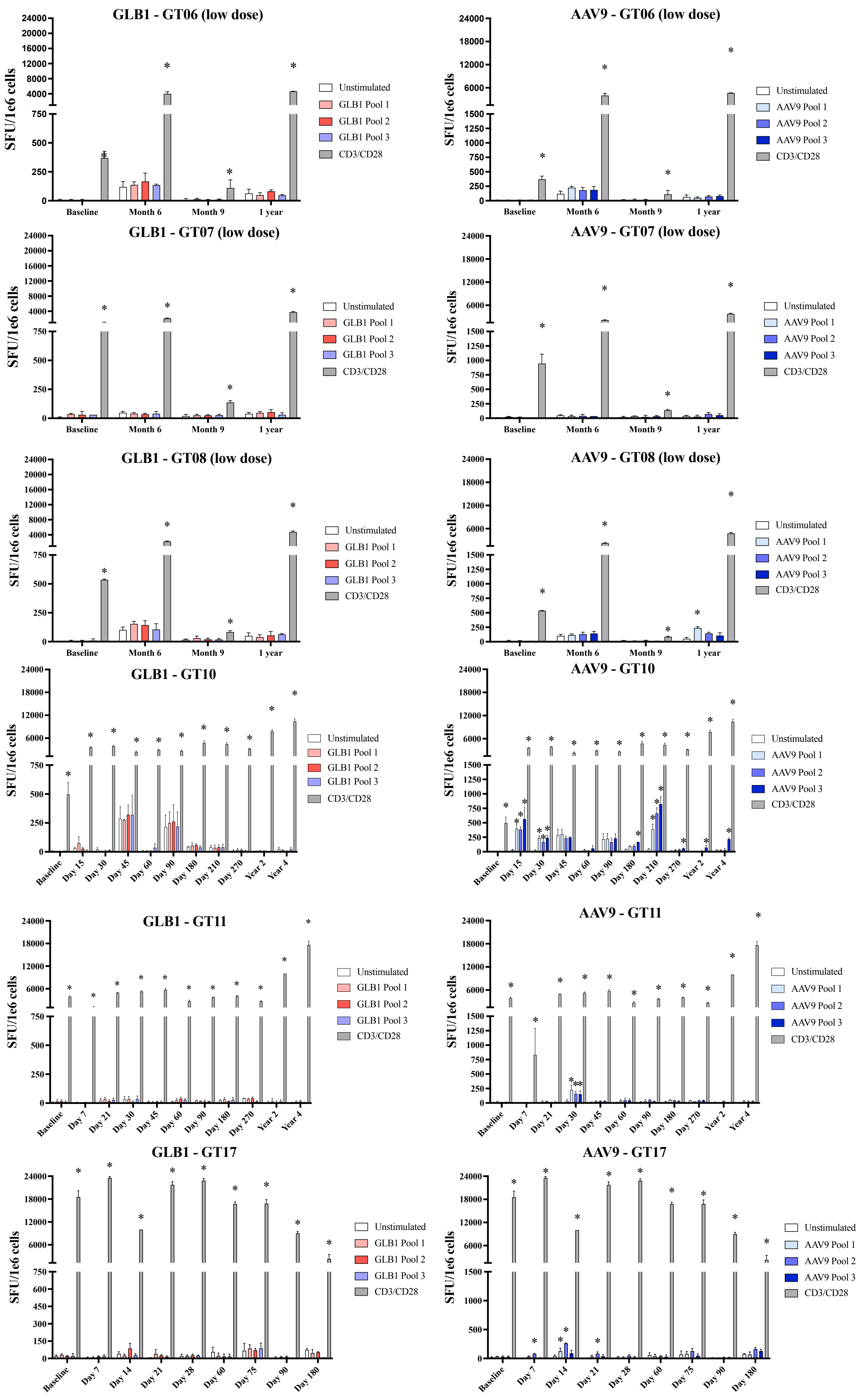

medRxiv preprint doi: https://doi.org/10.1101/2025.07.28.25332074; this version posted July 29, 2025. The copyright holder for this preprint (which was not certified by peer review) is the author/funder, who has granted medRxiv a license to display the preprint in perpetuity. This article is a US Government work. It is not subject to copyright under 17 USC 105 and is also made available for use under a CC0 license.

Figure J4. Transgene and Capsid specific ELISpots: IFN-γ ELISpot results for transgene specific (left) and capsid specific (right) immune responses. Positive responses designated by * and determined if they were ≥ 50 SFU/1e6 cells and at least 3X unstimulated negative control. CD3/CD28 stimulation was used as positive control. Positive and negative controls were run on all participants at all timepoints.

medRxiv preprint doi: https://doi.org/10.1101/2025.07.28.25332074; this version posted July 29, 2025. The copyright holder for this preprint (which was not certified by peer review) is the author/funder, who has granted medRxiv a license to display the preprint in perpetuity.
This article is a US Government work. It is not subject to copyright under 17 USC 105 and is also made available for use under a CC0 license.

Supplement K: Individualized β-galactosidase, GM1 Ganglioside, and H3N2B Levels

Late-Infantile

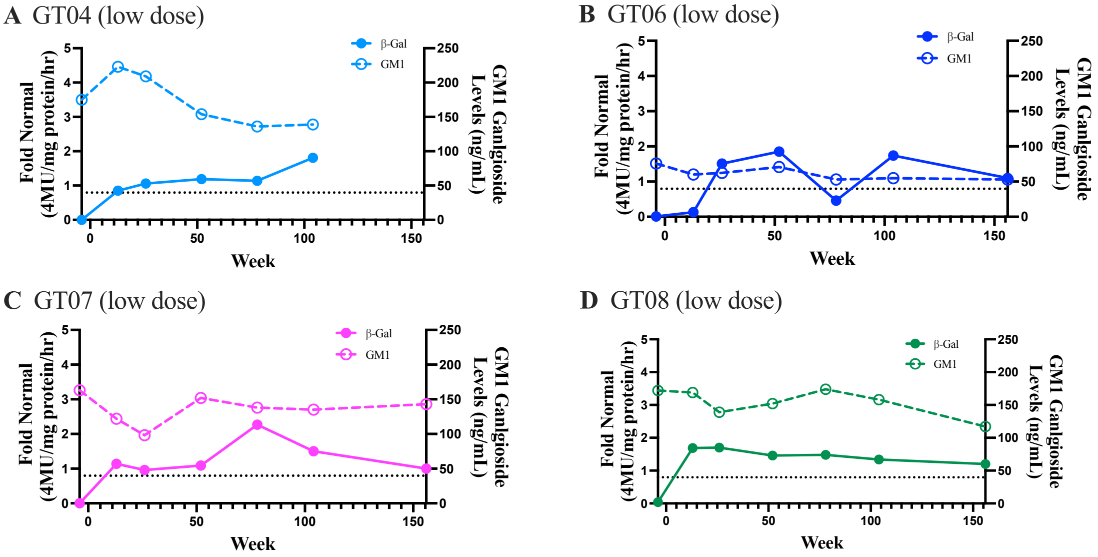

Juvenile

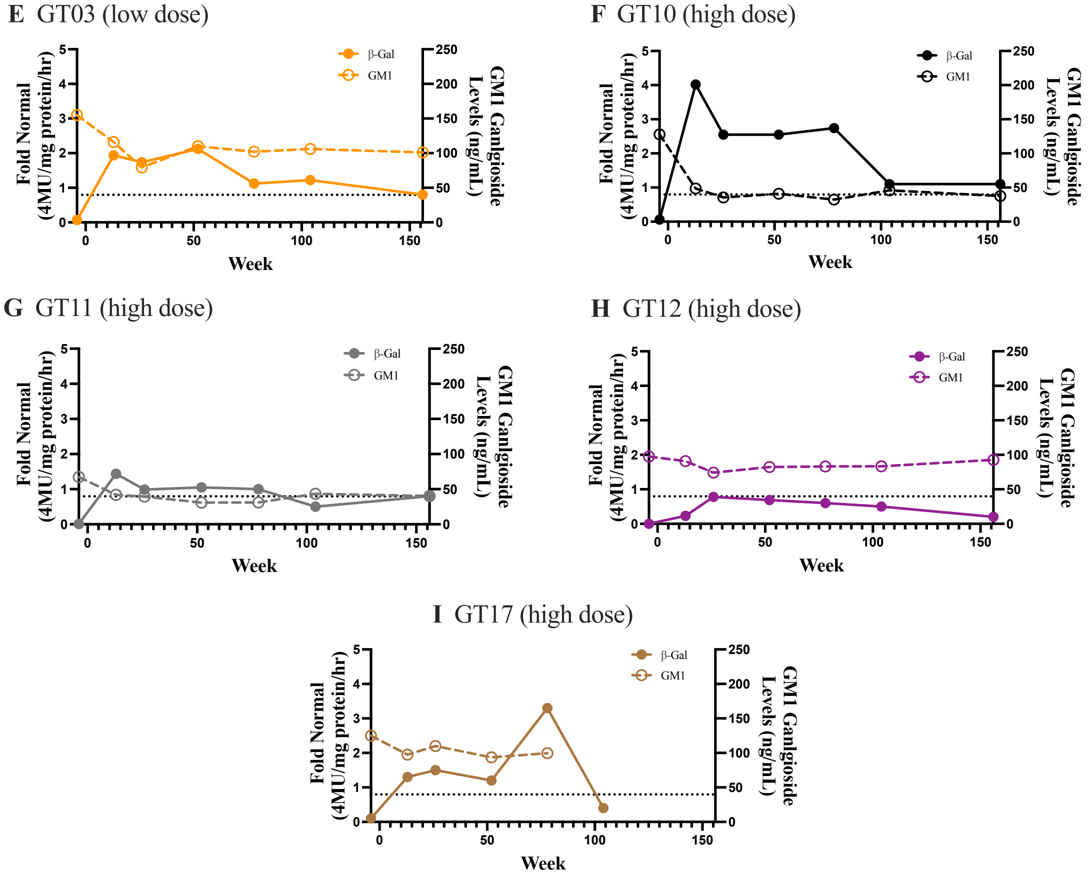

Figure K1. β-galactosidase (closed circles) and GM1 levels (open circles) in CSF. The dotted line on each graph represents the upper limit of normal for GM1 ganglioside levels in CSF (39.9 ng/mL at 1 standard deviation above normal).[37]

medRxiv preprint doi: https://doi.org/10.1101/2025.07.28.25332074; this version posted July 29, 2025. The copyright holder for this preprint (which was not certified by peer review) is the author/funder, who has granted medRxiv a license to display the preprint in perpetuity.
This article is a US Government work. It is not subject to copyright under 17 USC 105 and is also made available for use under a CC0 license.

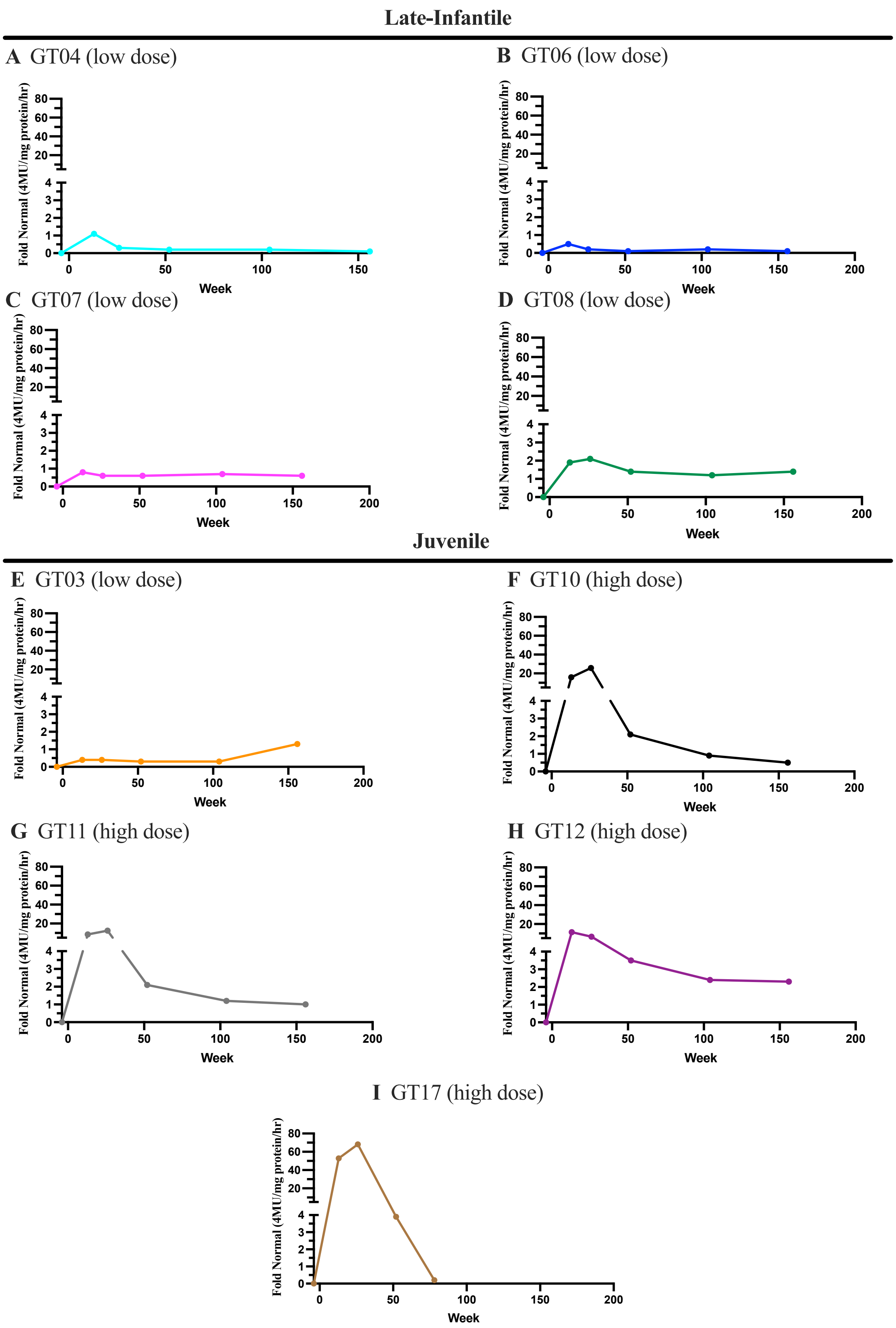

Figure K2. Serum β-galactosidase Levels for Both Dose Groups.

medRxiv preprint doi: https://doi.org/10.1101/2025.07.28.25332074; this version posted July 29, 2025. The copyright holder for this preprint (which was not certified by peer review) is the author/funder, who has granted medRxiv a license to display the preprint in perpetuity.
This article is a US Government work. It is not subject to copyright under 17 USC 105 and is also made available for use under a CC0 license.

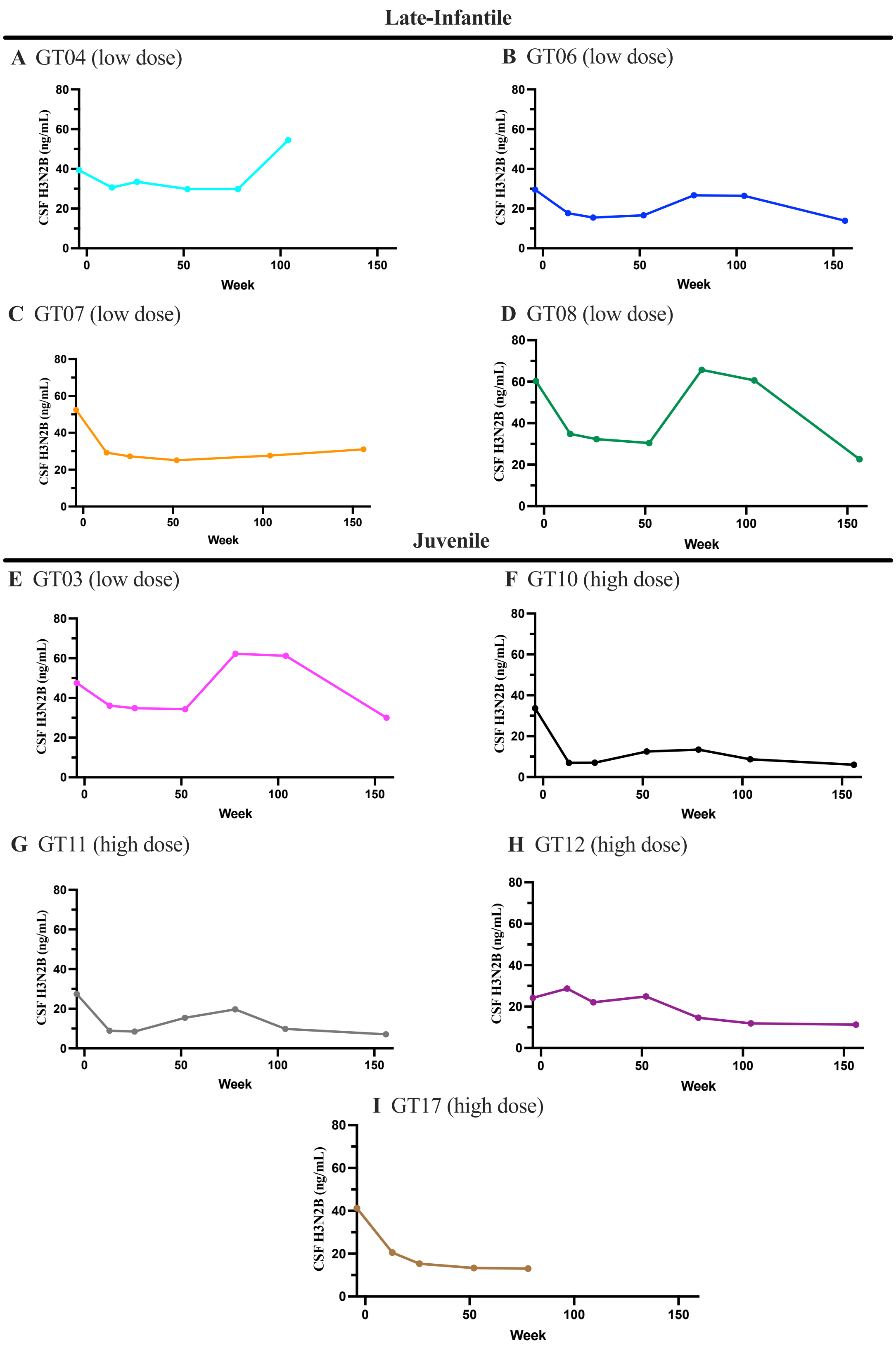

Figure K3. H3N2b Levels in CSF (ng/mL).

medRxiv preprint doi: https://doi.org/10.1101/2025.07.28.25332074; this version posted July 29, 2025. The copyright holder for this preprint (which was not certified by peer review) is the author/funder, who has granted medRxiv a license to display the preprint in perpetuity.
This article is a US Government work. It is not subject to copyright under 17 USC 105 and is also made available for use under a CC0 license.

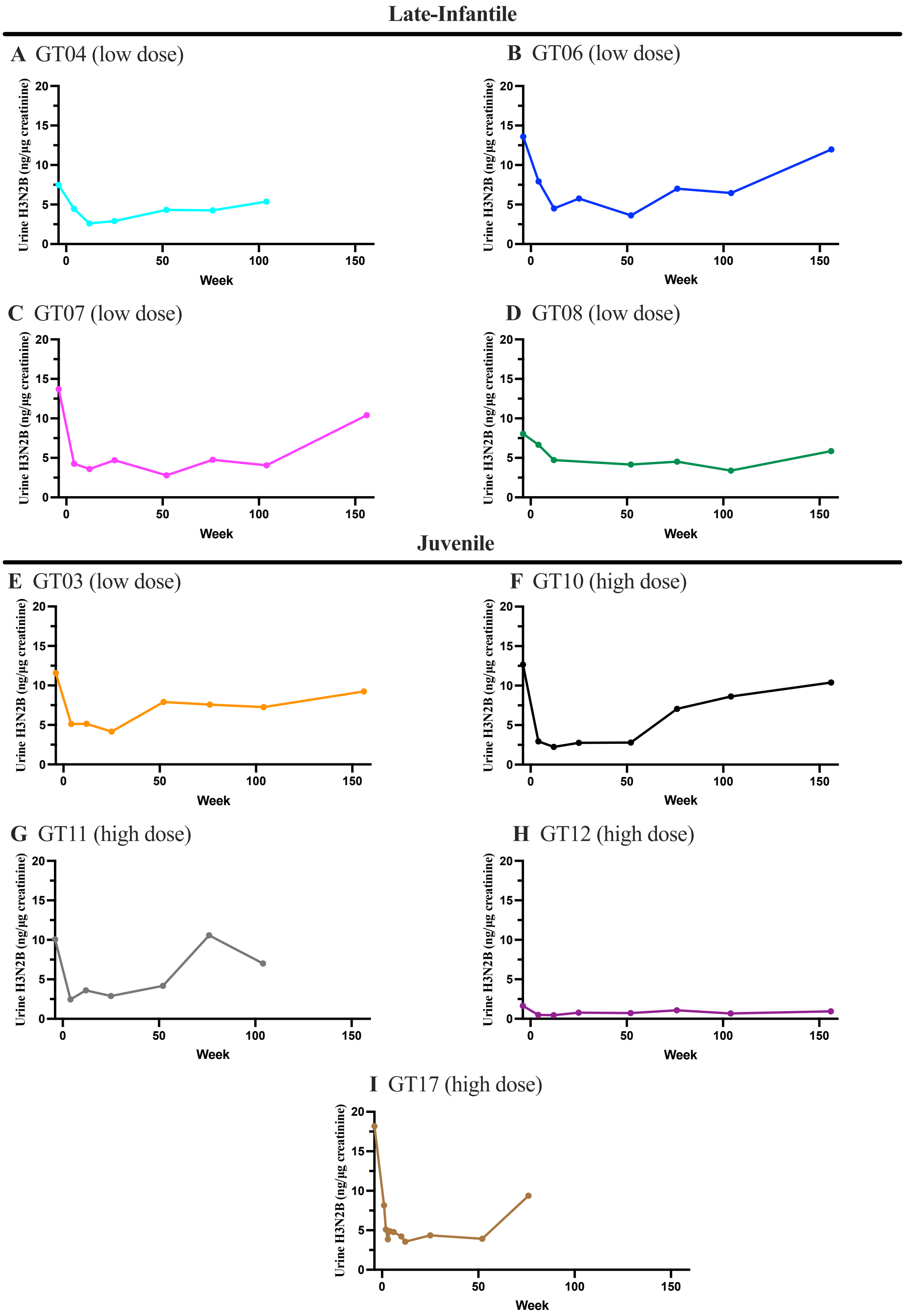

Figure K4. H3N2b Levels in Urine. H3N2b levels were normalized to human creatinine levels (ng/mL creatinine).

medRxiv preprint doi: https://doi.org/10.1101/2025.07.28.25332074; this version posted July 29, 2025. The copyright holder for this preprint (which was not certified by peer review) is the author/funder, who has granted medRxiv a license to display the preprint in perpetuity.
This article is a US Government work. It is not subject to copyright under 17 USC 105 and is also made available for use under a CC0 license.

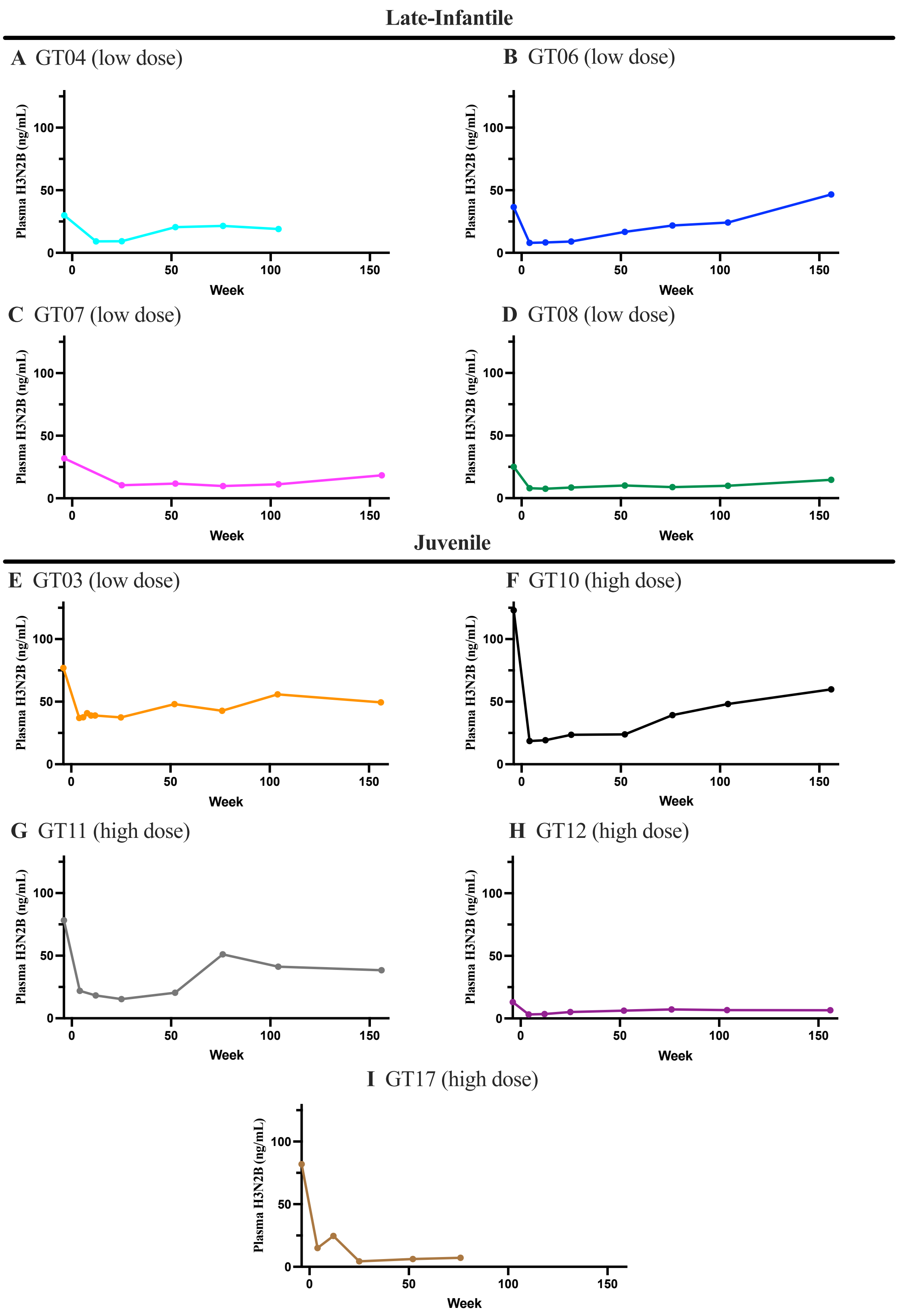

Figure K5. H3N2b Levels in Plasma (ng/mL).

medRxiv preprint doi: https://doi.org/10.1101/2025.07.28.25332074; this version posted July 29, 2025. The copyright holder for this preprint (which was not certified by peer review) is the author/funder, who has granted medRxiv a license to display the preprint in perpetuity. This article is a US Government work. It is not subject to copyright under 17 USC 105 and is also made available for use under a CC0 license.

Supplement L: Clinical Outcome Assessments

Vineland Adaptive Behavior Growth Scale Values (GSV)

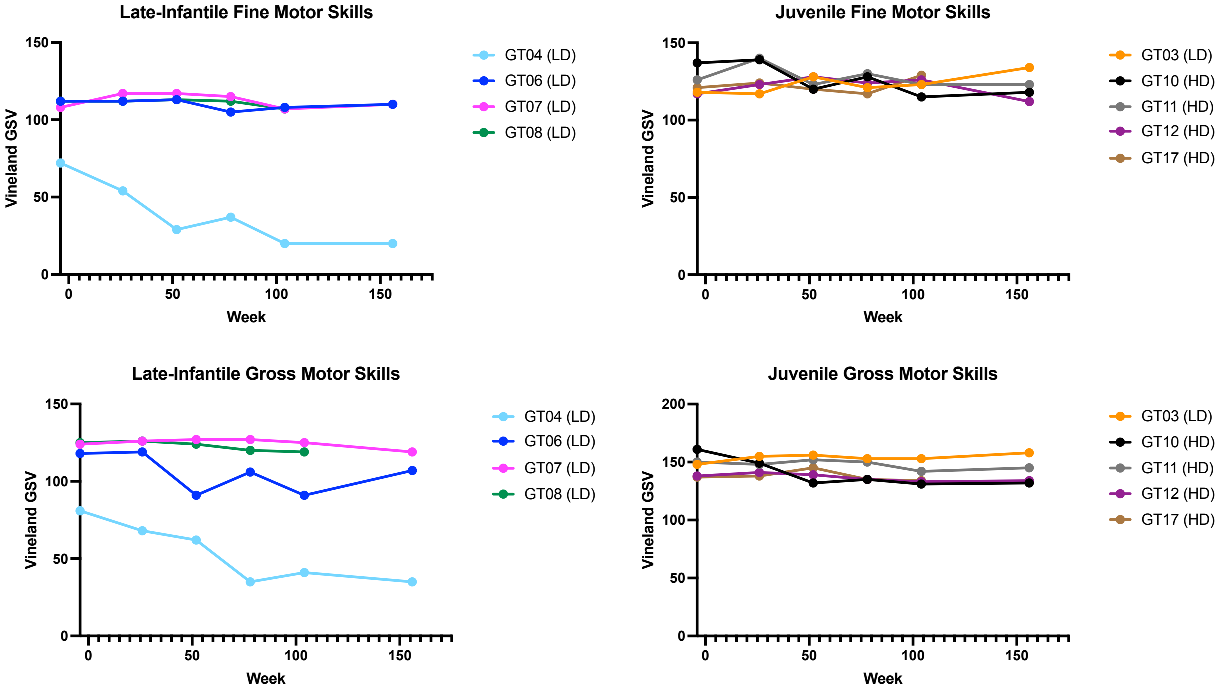

Figure L1. Vineland Adaptive Behavioral Growth Scale Values for fine and gross motor skills. Late-infantile GM1 participants are shown on the left, and juvenile participants are shown on the right with LD corresponding to participants who received low dose, and HD corresponding to high dose administration of AAV9-GLB1. In the late-infantile cohort, GT06, GT07, and GT08 demonstrated stability in both fine and gross motor skills, where GT04 showed a slight decline in both language domains. Juvenile participants showed stability in both fine and gross motor skills.

medRxiv preprint doi: https://doi.org/10.1101/2025.07.28.25332074; this version posted July 29, 2025. The copyright holder for this preprint
(which was not certified by peer review) is the author/funder, who has granted medRxiv a license to display the preprint in perpetuity.
This article is a US Government work. It is not subject to copyright under 17 USC 105 and is also made available
for use under a CC0 license.

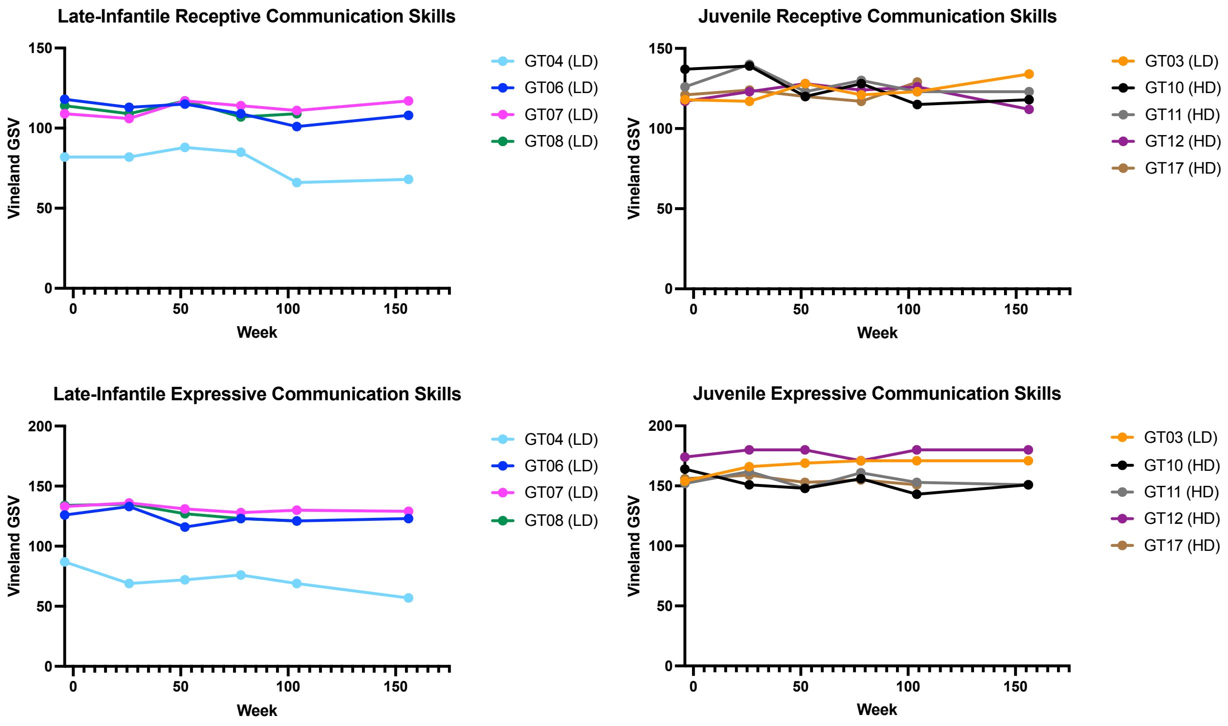

Figure L2. Vineland Adaptive Behavioral Growth Scale Values for Receptive and Expressive Communication skills. Late-infantile GM1 participants are shown on the left, and juvenile participants are shown on the right with LD corresponding to participants who received low dose, and HD corresponding to high dose administration of AAV9-GLB1. In the late-infantile cohort, GT06, GT07, and GT08 demonstrated stability in both expressive and receptive communication, where GT04 showed a slight decline in both communication domains. Juvenile participants showed stability in both expressive and receptive communication skills.

medRxiv preprint doi: https://doi.org/10.1101/2025.07.28.25332074; this version posted July 29, 2025. The copyright holder for this preprint (which was not certified by peer review) is the author/funder, who has granted medRxiv a license to display the preprint in perpetuity. This article is a US Government work. It is not subject to copyright under 17 USC 105 and is also made available for use under a CC0 license.

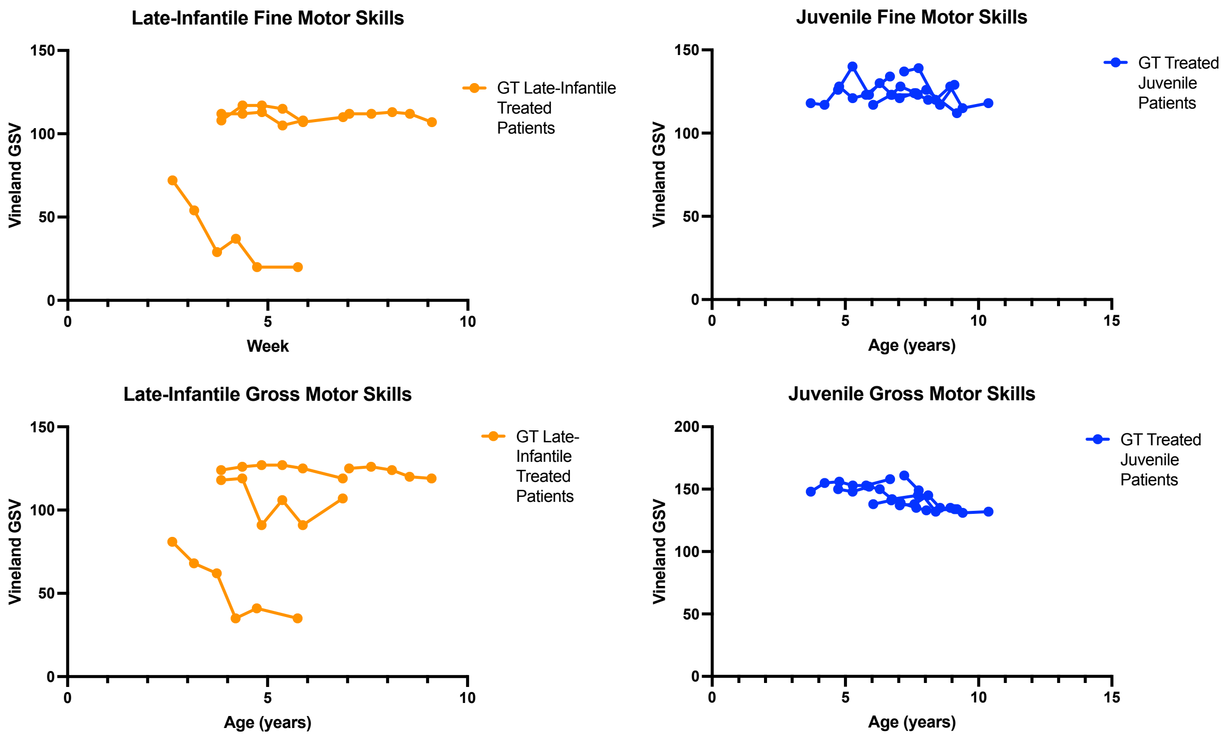

Figure L3. Vineland Adaptive Behavioral Growth Scale Values for fine and gross motor skills shown by patient age. Late-infantile GM1 participants are shown on the left, and juvenile participants are shown on the right with LD corresponding to participants who received low dose, and HD corresponding to high dose administration of AAV9-GLB1. Participant specific designations were redacted per MedArXiv requirements.

medRxiv preprint doi: https://doi.org/10.1101/2025.07.28.25332074; this version posted July 29, 2025. The copyright holder for this preprint
(which was not certified by peer review) is the author/funder, who has granted medRxiv a license to display the preprint in perpetuity.
This article is a US Government work. It is not subject to copyright under 17 USC 105 and is also made available
for use under a CC0 license.

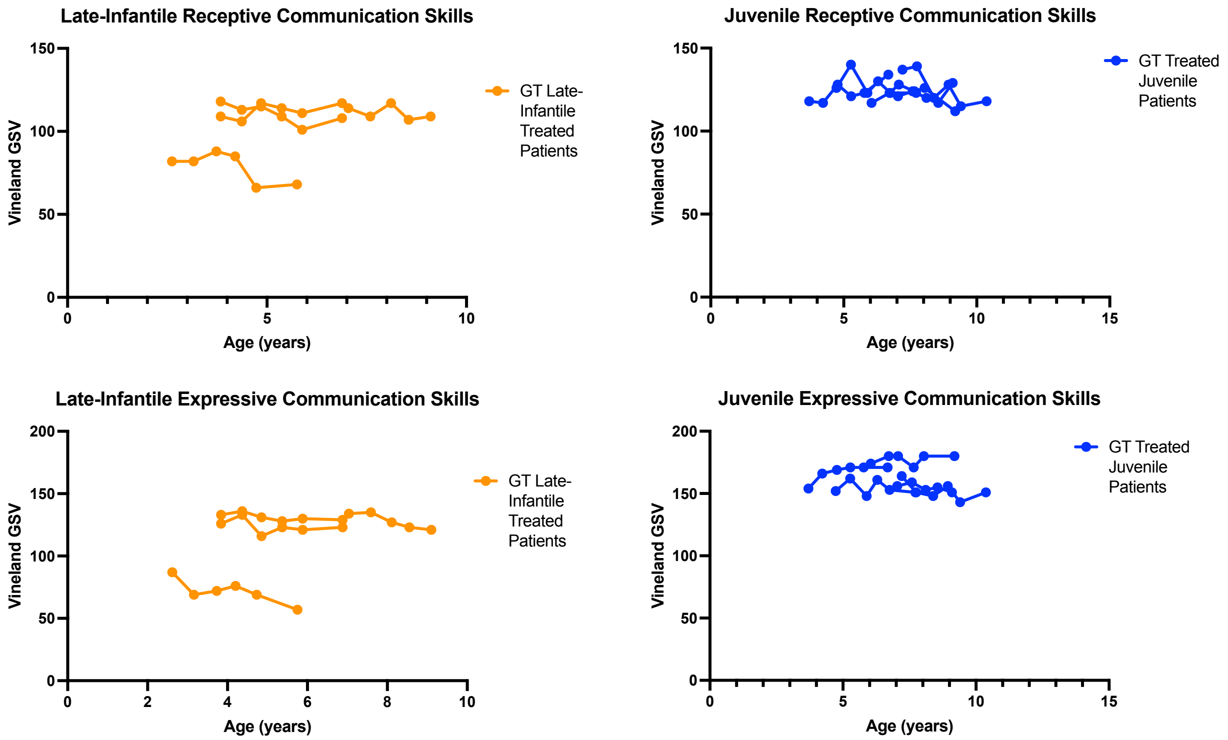

Figure L4. Vineland Adaptive Behavioral Growth Scale Values for Receptive and Expressive Communication skills shown by patient age. Late-infantile GM1 participants are shown on the left, and juvenile participants are shown on the right with LD corresponding to participants who received low dose, and HD corresponding to high dose administration of AAV9-GLB1. Participant specific designations were redacted per MedArXiv requirements.

medRxiv preprint doi: https://doi.org/10.1101/2025.07.28.25332074; this version posted July 29, 2025. The copyright holder for this preprint (which was not certified by peer review) is the author/funder, who has granted medRxiv a license to display the preprint in perpetuity.
This article is a US Government work. It is not subject to copyright under 17 USC 105 and is also made available for use under a CC0 license.

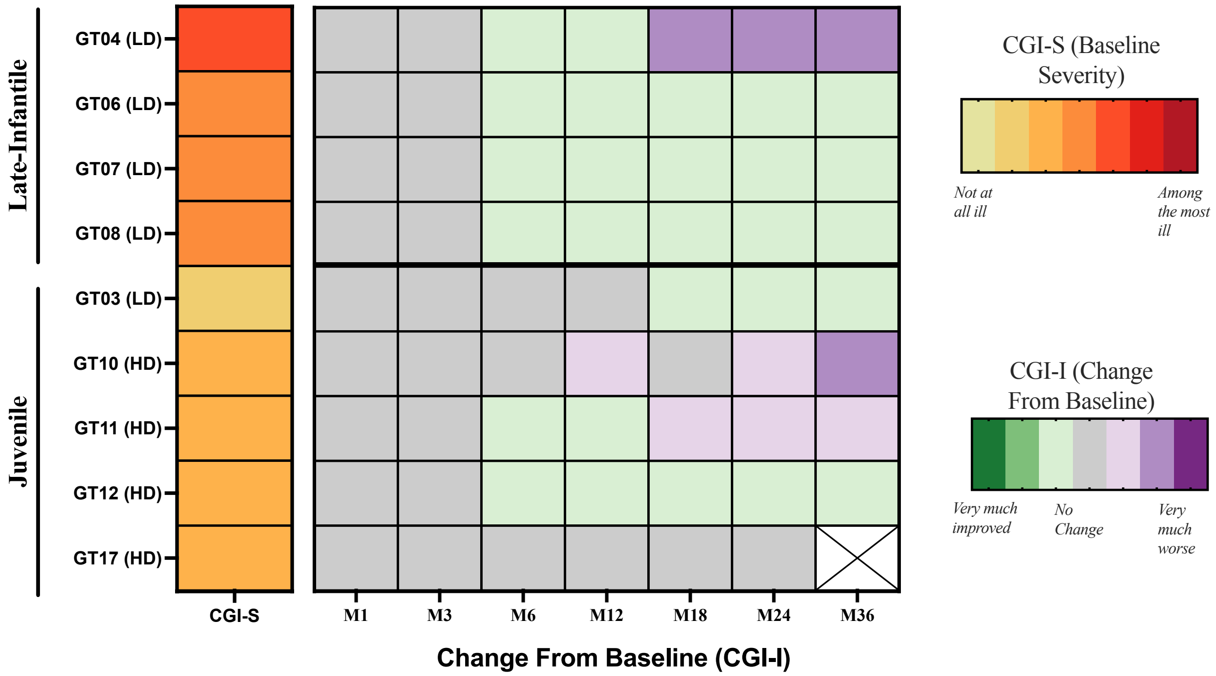

Figure L5. Baseline Clinical Global Impression scales of severity (CGI-S) and Clinical Global Impression of Improvement (CGI-I).

medRxiv preprint doi: https://doi.org/10.1101/2025.07.28.25332074; this version posted July 29, 2025. The copyright holder for this preprint (which was not certified by peer review) is the author/funder, who has granted medRxiv a license to display the preprint in perpetuity. This article is a US Government work. It is not subject to copyright under 17 USC 105 and is also made available for use under a CC0 license.

Supplement M: Neuroimaging Results

T1-Weighted Results – Brain Volume

Late-Infantile

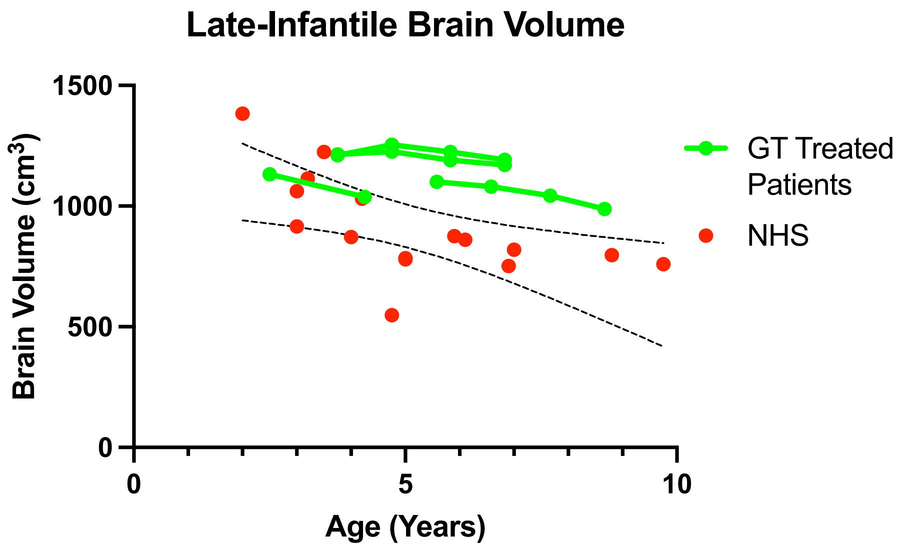

Juvenile

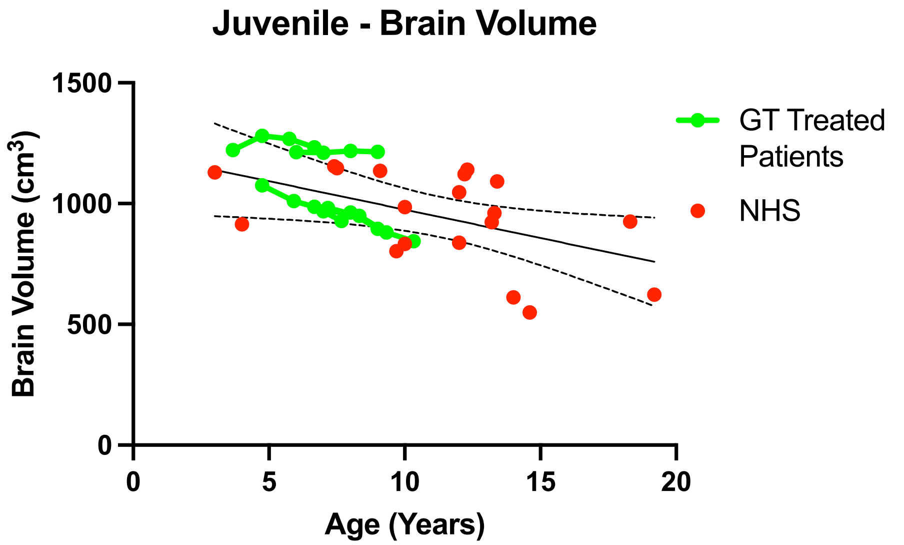

Figure M1. Quantification of cerebral atrophy in treated GM1 participants compared to untreated late-GM1 participants from the NHGRI natural history study (red). A simple linear regression was performed on the untreated natural history data to define the normal trajectory of both the late-infantile (n = 11) and juvenile (n = 18) diseases (with a 95% CI shown as the dotted line). Late-infantile participants GT07 and GT08 showed improvement in the rate of cerebral atrophy compared to the average change in untreated late-infantile participants. Juvenile participants GT03 and GT12 showed improvement in the rate of cerebral atrophy compared to the average change in untreated juvenile participants. Individual participant graphs were redacted per MedArXiv requirements.

medRxiv preprint doi: https://doi.org/10.1101/2025.07.28.25332074; this version posted July 29, 2025. The copyright holder for this preprint (which was not certified by peer review) is the author/funder, who has granted medRxiv a license to display the preprint in perpetuity. This article is a US Government work. It is not subject to copyright under 17 USC 105 and is also made available for use under a CC0 license.

T1-Weighted Results – Ventricle Volume

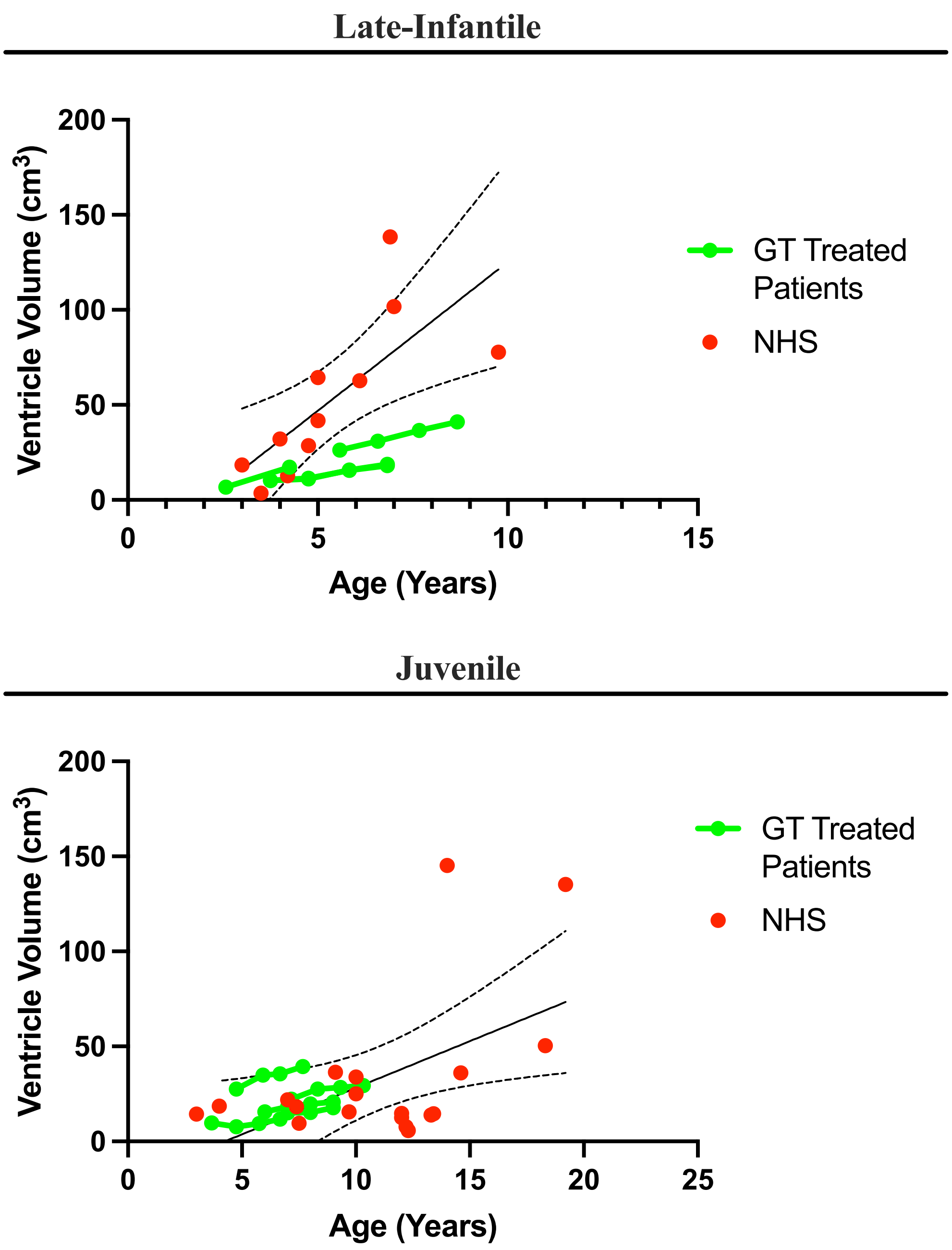

Figure M2. Quantification of ventricle volume in treated GM1 participants compared to untreated GM1 participants from the NHGRI natural history study (red). A simple linear regression was performed on the untreated natural history data to define the normal trajectory of both the late-infantile (n = 11) and juvenile (n = 18) diseases (with a 95% CI shown as the dotted line). Late-infantile participants GT04, GT06, GT07, and GT08 all showed improvement in the rate of ventricular enlargement compared to the average change in untreated late-infantile participants. Juvenile participants GT03, GT10, GT12, and GT17 all showed improvement in the rate of ventricular enlargement compared to the average change in untreated juvenile participants. Individual participant graphs were redacted per MedArXiv requirements.

medRxiv preprint doi: https://doi.org/10.1101/2025.07.28.25332074; this version posted July 29, 2025. The copyright holder for this preprint (which was not certified by peer review) is the author/funder, who has granted medRxiv a license to display the preprint in perpetuity.
This article is a US Government work. It is not subject to copyright under 17 USC 105 and is also made available for use under a CC0 license.

T1-Weighted Results – Thalamic Volume

Late-Infantile

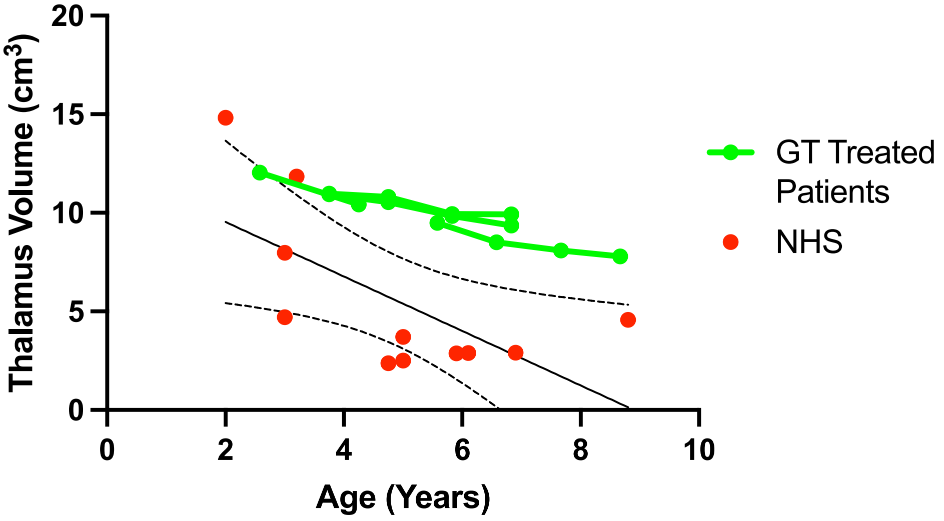

Juvenile

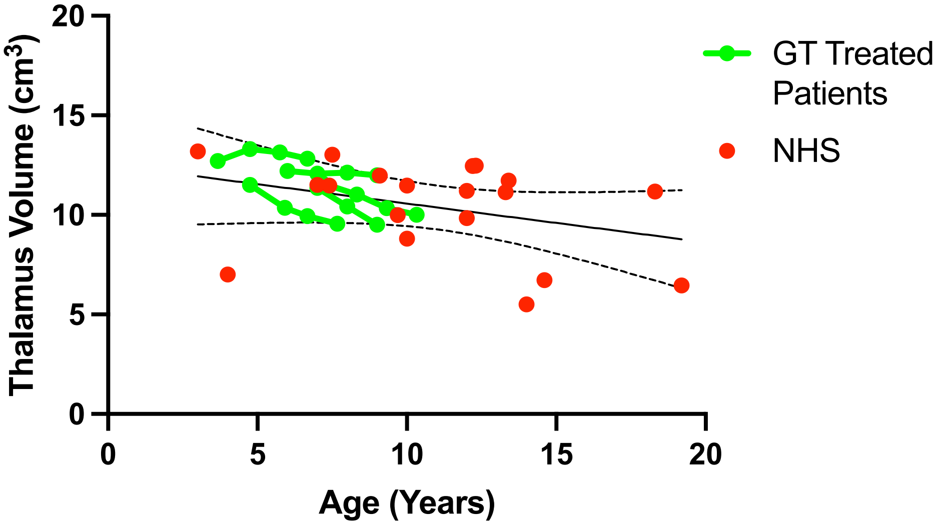

Figure M3. Quantification of thalamic volume in treated GM1 participants compared to untreated GM1 participants from the NHGRI natural history study (red). A simple linear regression was performed on the untreated natural history data to define the normal trajectory of both the late-infantile (n = 11) and juvenile (n = 18) diseases (with a 95% CI shown as the dotted line). Late-infantile participants GT04, GT06, GT07, and GT08 all showed improvement in the rate of thalamic atrophy compared to the average change in untreated late-infantile participants. Juvenile participants GT03 and GT12 showed improvement in the rate of thalamic atrophy compared to the average change in untreated juvenile participants. Individual participant graphs were redacted per MedArXiv requirements.

medRxiv preprint doi: https://doi.org/10.1101/2025.07.28.25332074; this version posted July 29, 2025. The copyright holder for this preprint (which was not certified by peer review) is the author/funder, who has granted medRxiv a license to display the preprint in perpetuity. This article is a US Government work. It is not subject to copyright under 17 USC 105 and is also made available for use under a CC0 license.

DT Results – Net Fiber Tract Metrics

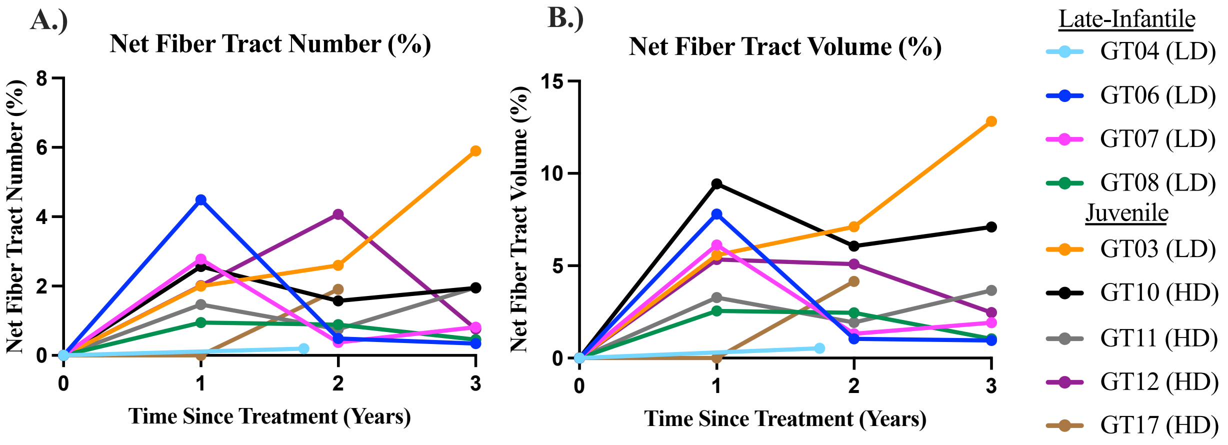

Figure M4. Quantification of Differential Tractography. A.) Net fiber number changes as a percentage relative to baseline tractography, individualized by patient. B.) Net fiber volume changes as a percentage relative to baseline tractography, individualized by patient.

medRxiv preprint doi: https://doi.org/10.1101/2025.07.28.25332074; this version posted July 29, 2025. The copyright holder for this preprint (which was not certified by peer review) is the author/funder, who has granted medRxiv a license to display the preprint in perpetuity.
This article is a US Government work. It is not subject to copyright under 17 USC 105 and is also made available for use under a CC0 license.

<u>MRS – LCSO – *N*-Acetylaspartete + *N*-Acetylaspartyl glutamate (NAA)</u>

Late-Infantile

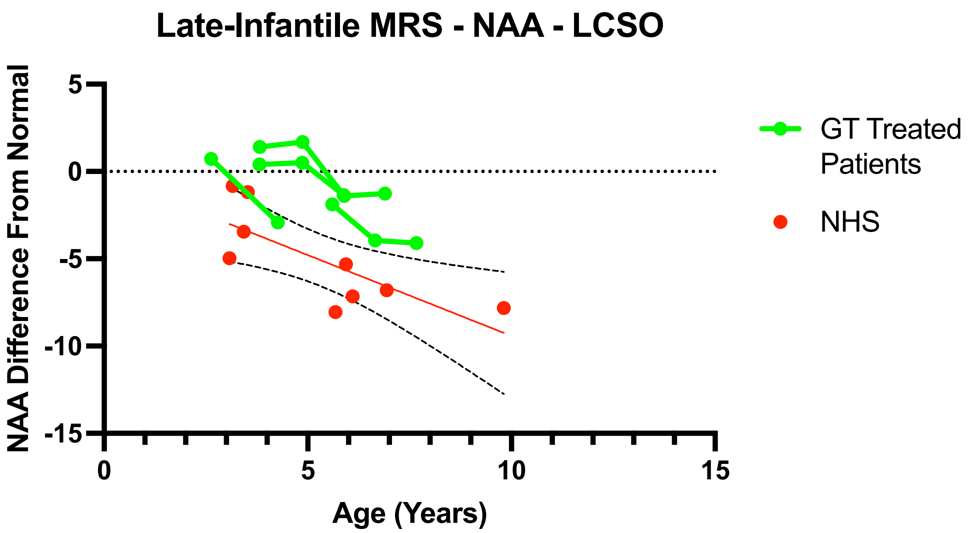

Juvenile

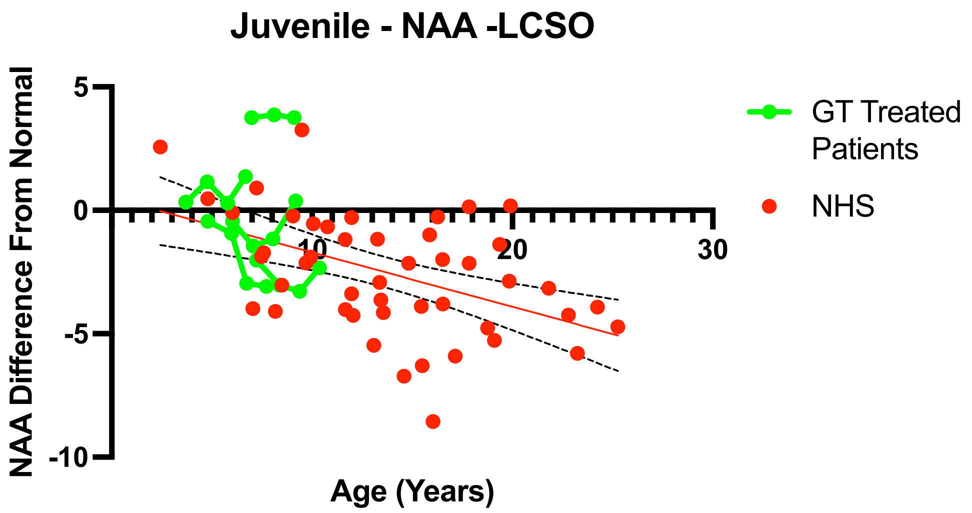

Figure M5. Quantification of *N*-Acetylaspartate + *N*-Acetylaspartyl glutamate (NAA) in the left centrum semiovale (LCSO) in treated GM1 participants compared to untreated GM1 participants from the NHGRI natural history study (red). A simple linear regression was performed on the untreated natural history data to define the normal trajectory of both the late-infantile (n = 9) and juvenile (n = 17) diseases (with a 95% CI shown as the dotted line). Late-infantile participants GT07 and GT08 showed stabilization of the NAA decreases associated with late-infantile GM1 disease progression in the year following gene transfer. GT08 showed a diminished rate of NAA compared to the untreated GM1 participants in the second year following gene transfer. Juvenile participants GT03 GT10, and GT12 all showed stabilization of the NAA decreases associated with juvenile GM1 disease progression. Individual participant graphs were redacted per MedArXiv requirements.

medRxiv preprint doi: https://doi.org/10.1101/2025.07.28.25332074; this version posted July 29, 2025. The copyright holder for this preprint (which was not certified by peer review) is the author/funder, who has granted medRxiv a license to display the preprint in perpetuity. This article is a US Government work. It is not subject to copyright under 17 USC 105 and is also made available for use under a CC0 license.

Supplementary References

1. D'Souza P, Farmer C, Johnston JM, et al. GM1 gangliosidosis type II: Results of a 10-year prospective study. Genet Med. 2024;26(7):101144. doi:10.1016/j.gim.2024.101144.

2. Patterson, Marc C. "Gangliosidoses." Handbook of clinical neurology 113 (2013): 1707-1708.

3. Regier, Debra S., Cynthia J. Tifft, and Caroline E. Rothermel. "GLB1-related disorders." (2021).

4. Flotte TR, Cataltepe O, Puri A, et al. AAV gene therapy for Tay-Sachs disease. Nat Med. 2022;28(2):251-259. doi:10.1038/s41591-021-01664-4.

5. Kell P, Sidhu R, Qian M, et al. A pentasaccharide for monitoring pharmacodynamic response to gene therapy in GM1 gangliosidosis. EBioMedicine. 2023;92:104627. doi:10.1016/j.ebiom.2023.104627.

6. Leonard M, Dunn J, Smith G. A clinical biomarker assay for the quantification of d3-creatinine and creatinine using LC-MS/MS. Bioanalysis. 2014;6(6):745-759. doi:10.4155/bio.13.323.

7. Leon-Astudillo C, Coleman K, Salabarria SM, et al. Quantification and comparison of anti-AAV9 and anti-AAVrh74 antibodies in plasma and human milk: Implications for AAV-based gene therapy candidacy. J Neuromuscul Dis. Published online April 10, 2025. doi:10.1177/22143602251324857

8. Sparrow SS, Cicchetti DV, Saulnier CA. Vineland-3: Vineland adaptive behavior scales. PsychCorp; 2016.

9. Farmer C, Ludwig, NN, Thurm A. A tutorial on person ability scores for the intellectual and developmental disabilities clinician. International Journal of Developmental Disabilities, (2025)1–10. https://doi.org/10.1080/20473869.2025.2486428.

10. Farmer C, Thurm A, Troy JD, Kaat AJ. Comparing ability and norm-referenced scores as clinical trial outcomes for neurodevelopmental disabilities: a simulation study. J Neurodev Disord. 2023;15(1):4. Published 2023 Jan 17. doi:10.1186/s11689-022-09474-6.

11. Bates D, Mächler M, Bolker B, Walker S. Fitting linear mixed-effects models using lme4. *arXiv preprint arXiv:14065823*. 2014.

12. Kuznetsova A, Brockhoff PB, Christensen RH. lmerTest package: tests in linear mixed effects models. *Journal of statistical software*. 2017;82:1-26. in R version 4.4.2.

13. Guy W. ECDEU assessment manual for psychopharmacology. US Department of Health, Education, and Welfare, Public Health Service, 1976.

14. Busner J, Targum SD. The clinical global impressions scale: applying a research tool in clinical practice. Psychiatry (Edgmont). 2007;4(7):28-37.

15. Lewis CJ, Johnston JM, Zaragoza Domingo S, et al. Retrospective assessment of clinical global impression of severity and change in GM1 gangliosidosis: a tool to score natural history data in rare disease cohorts. Orphanet J Rare Dis. 2025;20(1):125. Published 2025 Mar 14. doi:10.1186/s13023-025-03614-6.

16. National Human Genome Research Institute. Natural History of Glycosphingolipid Storage Disorders and Glycoprotein Disorders. ClinicalTrials.gov identifier: NCT00029965. https://clinicaltrials.gov/study/NCT00029965.

17. National Human Genome Research Institute. A Phase 1/2 Study of Intravenous Gene Transfer with an AAV9 Vector Expressing Human Beta-galactosidase in Type I and

medRxiv preprint doi: https://doi.org/10.1101/2025.07.28.25332074; this version posted July 29, 2025. The copyright holder for this preprint (which was not certified by peer review) is the author/funder, who has granted medRxiv a license to display the preprint in perpetuity. This article is a US Government work. It is not subject to copyright under 17 USC 105 and is also made available for use under a CC0 license.

Type II GM1 Gangliosidosis. ClinicalTrials.gov identifier: NCT03952637. https://clinicaltrials.gov/study/NCT03952637.

18. Kolstad J, Zoppo C, Johnston JM, et al. Natural history progression of MRI brain volumetrics in type II late-infantile and juvenile GM1 gangliosidosis patients. Mol Genet Metab. 2025;144(3):109025. doi:10.1016/j.ymgme.2025.109025.

19. Lewis CJ, Vardar Z, Kühn AL, et al. Differential tractography: an imaging marker for tissue degeneration in neurodegenerative diseases. Brain Communications, 2025, fcaf198, https://doi.org/10.1093/braincomms/fcaf198.

20. Li X, Morgan PS, Ashburner J, Smith J, Rorden C. The first step for neuroimaging data analysis: DICOM to NIfTI conversion. J Neurosci Methods. 2016;264:47-56. doi:10.1016/j.jneumeth.2016.03.001.

21. Manjón JV, Romero JE, Vivo-Hernando R, et al. vol2Brain: A New Online Pipeline for Whole Brain MRI Analysis. Front Neuroinform. 2022;16:862805. Published 2022 May 24. doi:10.3389/fninf.2022.862805.

22. Lewis CJ, Johnston JM, D'Souza P, et al. A Case for Automated Segmentation of MRI Data in Neurodegenerative Diseases: Type II GM1 Gangliosidosis. NeuroSci. 2025;6(2):31. Published 2025 Apr 3. doi:10.3390/neurosci6020031.

23. Yeh FC, Zaydan IM, Suski VR, et al. Differential tractography as a track-based biomarker for neuronal injury. Neuroimage. 2019;202:116131. doi:10.1016/j.neuroimage.2019.116131.

24. Tournier JD, Smith R, Raffelt D, et al. MRtrix3: A fast, flexible and open software framework for medical image processing and visualisation. Neuroimage. 2019;202:116137. doi:10.1016/j.neuroimage.2019.116137.

25. Andersson JLR, Sotiropoulos SN. An integrated approach to correction for off-resonance effects and subject movement in diffusion MR imaging. Neuroimage. 2016;125:1063-1078. doi:10.1016/j.neuroimage.2015.10.019.

26. Smith SM, Jenkinson M, Woolrich MW, et al. Advances in functional and structural MR image analysis and implementation as FSL. Neuroimage. 2004;23 Suppl 1:S208-S219. doi:10.1016/j.neuroimage.2004.07.051.

27. Dhollander T, Raffelt D, Connelly A. Unsupervised 3-tissue response function estimation from single-shell or multi-shell diffusion MR data without a co-registered T1 image. ISMRM Workshop on Breaking the Barriers of Diffusion MRI, 2016, 5.

28. Jenkinson M, Beckmann CF, Behrens TE, Woolrich MW, Smith SM. FSL. Neuroimage. 2012;62(2):782-790. doi:10.1016/j.neuroimage.2011.09.015.

29. Woolrich MW, Jbabdi S, Patenaude B, et al. Bayesian analysis of neuroimaging data in FSL. Neuroimage. 2009;45(1 Suppl):S173-S186. doi:10.1016/j.neuroimage.2008.10.055.

30. Yeh FC, Wedeen VJ, Tseng WY. Generalized q-sampling imaging. IEEE Trans Med Imaging. 2010;29(9):1626-1635. doi:10.1109/TMI.2010.2045126.

31. Provencher SW. Estimation of metabolite concentrations from localized in vivo proton NMR spectra. *Magnetic resonance in medicine*. 1993;30(6):672-679.

32. Ernst T, Kreis R, Ross B. Absolute quantitation of water and metabolites in the human brain. I. Compartments and water. *Journal of magnetic resonance, Series B*. 1993;102(1):1-8.

medRxiv preprint doi: https://doi.org/10.1101/2025.07.28.25332074; this version posted July 29, 2025. The copyright holder for this preprint (which was not certified by peer review) is the author/funder, who has granted medRxiv a license to display the preprint in perpetuity. This article is a US Government work. It is not subject to copyright under 17 USC 105 and is also made available for use under a CC0 license.

33. Srinivasan R, Sailasuta N, Hurd R, Nelson S, Pelletier D. Evidence of elevated glutamate in multiple sclerosis using magnetic resonance spectroscopy at 3 T. *Brain*. 2005;128(5):1016-1025.

34. Baker EH, Levin SW, Zhang Z, Mukherjee AB. Evaluation of disease progression in INCL by MR spectroscopy. *Annals of clinical and translational neurology*. 2015;2(8):797-809.

35. Baker EH, Basso G, Barker PB, Smith MA, Bonekamp D, Horská A. Regional apparent metabolite concentrations in young adult brain measured by 1H MR spectroscopy at 3 Tesla. *Journal of Magnetic Resonance Imaging: An Official Journal of the International Society for Magnetic Resonance in Medicine*. 2008;27(3):489-499.

36. Horska A, Calhoun V, Bradshaw D, Barker P. Rapid method for correction of CSF partial volume in quantitative proton MR spectroscopic imaging. *Magnetic Resonance in Medicine: An Official Journal of the International Society for Magnetic Resonance in Medicine*. 2002;48(3):555-558.

37. Izumi T, Ogawa T, Koizumi H, Fukuyama Y. Normal developmental profiles of CSF gangliotetraose-series gangliosides from neonatal period to adolescence. Pediatr Neurol. 1993;9(4):297-300. doi:10.1016/0887-8994(93)90067-m.
